# Assessment of a Parachor Model for the Surface Tension of Binary Mixtures

**DOI:** 10.1007/s10765-023-03216-z

**Published:** 2023-06-07

**Authors:** Alexandra Metallinou Log, Vladimir Diky, Marcia L. Huber

**Affiliations:** 1 Department of Energy and Process Engineering, Faculty of Engineering, Norwegian University of Science and Technology, 7491 Trondheim, Norway; 2 Applied Chemicals and Materials Division, National Institute of Standards and Technology, 325 Broadway, Boulder, CO 80305 USA

**Keywords:** Binary mixtures, Parachor, Surface tension

## Abstract

We compiled an experimental database for the surface tension of binary mixtures containing a wide variety of fluids, from the chemical classes (water, alcohols, amines, ketones, linear and branched alkanes, naphthenes, aromatics, refrigerants, and cryogens). The resulting data set includes 65 pure fluids and 154 binary pairs with a total of 8205 points. We used this database to test the performance of a parachor model for the surface tension of binary mixtures. The model uses published correlations to determine the parachors of the pure fluids. The model has a single, constant binary interaction parameter for each pair that was found by fitting experimental mixture data. It can be also used in a predictive mode when the interaction parameters are set to zero. We present detailed comparisons on the performance of the model for both cases. In general, the parachor model in a predictive mode without fitted interaction parameters can predict the surface tension of binary mixtures of non-polar mixtures such as linear and branched alkanes, linear and branched alkanes with naphthenes, aromatics with aromatics, aromatics with naphthenes, and mixtures of linear alkanes of similar sizes with an average absolute percentage deviation of about 3 % or less. Polar mixtures of halocarbons with other halocarbons and also polar/nonpolar mixtures of alkanes with halocarbons could be modeled with an average absolute deviation of less than 0.35 mN·m^−1^ with the use of a binary interaction parameter. The parachor model even with a fitted binary interaction parameter performs poorly for mixtures of water and organic compounds and is not recommended.

**Supplementary Information:**

The online version contains supplementary material available at 10.1007/s10765-023-03216-z.

## Introduction

Surface tension is an important physical property that has long had significance in the oil and gas industry, and is also of interest in applications as varied as pharmaceuticals [1, 2], heat transfer in low-global warming potential (GWP) refrigerants [3], ink-jet printing [4, 5] and diesel engine design [6]. Specific examples in the pharmaceutical industry include [2] the importance of controlling the surface tension of coating solutions of tablets to improve product appearance and control the rate of drug release, the effect of surface tension on the size of droplets in a nebulizer, and control of the size of eye drops. In addition, fluorocarbon based fluids with low surface tension are being investigated as blood substitutes for oxygen delivery [1]. In the refrigeration industry, new low-GWP refrigerant blends are being proposed. In order to evaluate the performance of heat exchangers, accurate knowledge of the surface tension is needed to model the bubble behavior in pool boiling [3]. In ink-jet printing, From [5] analyzed the fluid flow behavior of impulsively driven laminar jet flow in terms of dimensionless parameters involving the surface tension, density, viscosity, and a characteristic dimension, and made recommendations for when the fluid has stable drop formation. In order to optimize engine performance to reduce soot emissions, there is a need for surface tension data at high pressures and high temperatures [6]. Accurate property values for surface tension are necessary for successful analysis of all these processes.

In 1923, Macleod proposed a simple empirical relationship between surface tension *σ* and the density of the liquid and vapor phases *ρ*_L_ and *ρ*_V_

1 $${\text{P}} = \frac{{\sigma^{1/4} }}{{\rho_{{\text{L}}} - \rho_{{\text{V}}} }},$$

where P is a temperature-independent parameter called the parachor by Sugden [7]. Other practical engineering methods for predicting surface tension can be found in handbooks such as Ref. [8]. In addition, there are numerous theoretically based approaches to predicting the surface tension such as density gradient theory [9, 10], density functional theory [11], hard-sphere fluid scaled particle theory [12], perturbation theory [13] and friction theory [14].

The parachor approach can also be applied to mixtures, as was demonstrated by Weinaug and Katz [15] and Hugill *et al.* [16]. Although the parachor method has been used for many years in the petrochemical industry, is in active use now [17], is the recommended approach in the API Technical Databook [18], and is discussed in reference books for engineers [8] there has not been a comprehensive evaluation of the performance of this type of model with respect to mixtures using a large database of binary data in the open literature. It is the goal of this work to provide an evaluation of the parachor model to a wide variety of binary mixtures, including not only common hydrocarbons involved in the petrochemical industry, but also recent low-GWP fluids of interest to the refrigeration industry and to indicate expected performance and limitations of this model for a wide variety of mixtures.

## The Parachor Model

The parachor model that we will apply to mixtures was originally presented by Weinaug and Katz [15] and later modified by Hugill *et al.* [16] to allow for the use of binary interaction parameters. For a mixture,

2 $$\sigma_{{{\text{mix}}}} = \left( {{\text{P}}_{{\text{L}}} \rho_{{\text{L}}} - {\text{P}}_{{\text{V}}} \rho_{{\text{V}}} } \right)^{m}$$

with mixing and combining rules

3 $${\text{P}}_{{\text{L}}} = \sum\limits_{{i = 1}}^{n} {\sum\limits_{{j = 1}}^{n} {x_{i} x_{j} {\text{P}}_{{ij}} \quad {\text{and}}\quad {\text{P}}_{{\text{V}}} = \sum\limits_{{i = 1}}^{n} {\sum\limits_{{j = 1}}^{n} {y_{i} y_{j} {\text{P}}_{{ij}} } } } }$$

4 $${\text{P}}_{ij} = (1 - \delta_{ij} )\frac{{{\text{P}}_{i} + {\text{P}}_{j} }}{2},$$

where *δ*_*ij*_ is an optional binary interaction parameter, and *x*_*i*_ and *y*_*i*_ are the molar compositions of the liquid and gas phases, respectively. Historically [15, 16, 19] the exponent *m* has been set to 4, but here we use *m* = 3.87 based on theoretical considerations as presented by Garrabos *et al.* [20]. In addition, it also is common to use a fixed value of the parachor obtained from compilations such as that of Quayle [21]. Zhelezny *et al.* [22] has studied the temperature dependence of the parachor. Mulero and coworkers [23–31] developed an extensive body of work on correlations for the surface tension of many important industrial fluids that can be used to compute the pure fluid parachors P_*i*_ as a function of temperature. These correlations are very accurate and can represent the data to within experimental uncertainty. We primarily use these correlations as implemented in the computer program REFPROP v10 [32] for pure fluid surface tension *σ*_*i.*_ The parachors are evaluated at the temperature of interest for the binary mixture, however for temperatures greater than or equal to 0.9*T*_c,i_, where *T*_c,i_ is the pure fluid critical temperature, the parachor is calculated at 0.9*T*_c,i_. It also is necessary to have the saturation densities and compositions *x*_*i*_ and *y*_*i*_ of the liquid and vapor phases. If the compositions and densities from the VLE calculations are inaccurate this will increase the uncertainty in the surface tension calculations, so care should be used in the selection of the VLE model. We obtain these compositions and densities from the default equations of state and models implemented in REFPROP v10 [32]; a description of these can be found in [33]. A few changes were made in the models of REFPROP v10 that enabled calculation for some mixtures not permitted in the original version, as well as some changes in mixture parameters that are summarized in the Supplementary Information in Appendix A.

## Experimental Data

We extracted experimental data for the surface tension of binary mixtures for liquid–gas interfaces from the NIST TDE database [34] for which the pure fluid components are available, the composition of the liquid is explicitly specified, and also for which there are reliable models for the vapor–liquid equilibrium and thermodynamic properties in the REFPROP database [32]. We excluded HCl/water and benzene/water due to the lack of a good mixture model for thermodynamic properties in REFPROP. The resulting data set includes 65 pure fluids and 154 binary pairs with a total of 8205 points. Table 1 provides a list of the pure components along with information for compound identification, along with a reference for the pure fluid surface tension correlation implemented in REFPROP v10 [32] used to evaluate pure fluid surface tension in this work. A summary of the binary mixture data is given in Table 2 including a reference code (starting with the publication year), the experimental method, an uncertainty estimate, the fluids in the binary mixture, the number of data points, temperature range, and composition in terms of the mole fraction of the first component. The full data set is available in the supplementary information in the file InputData.txt. A discussion of experimental methods for obtaining surface tension can be found in Ref. [35]. The estimated uncertainties (at a *k* = 2 level) are those as assessed by the NIST TDE database and may not be the same as those stated by the original authors. As part of the data capture process, software [36] is used that assesses the uncertainty of the data taking into account factors such as the experimental method, the sample purity, property precision, precision of independent variables. However, the reader should consult the original data reference for complete details of the measurement technique and uncertainty analysis for assessment of the quality of an individual data set.

**Table 1:** List of fluids

Name	Full name	Formula	Family	CAS no.	Standard InChI key	References
Acetone	Propanone	C_3_H_6_O	Ketone	67-64-1	CSCPPACGZOOCGX-UHFFFAOYSA-N	[26]
Argon	Argon	Ar	Cryogen	7440-37-1	XKRFYHLGVUSROY-UHFFFAOYSA-N	[26]
Benzene	Benzene	C_6_H_6_	Aromatic	71-43-2	UHOVQNZJYSORNB-UHFFFAOYSA-N	[26]
Butane	*n*-Butane	C_4_H_10_	*n*-Alkane	106-97-8	IJDNQMDRQITEOD-UHFFFAOYSA-N	[26]
Carbon dioxide	Carbon dioxide	CO_2_	Other	124-38-9	CURLTUGMZLYLDI-UHFFFAOYSA-N	[26]
Carbon monoxide	Carbon monoxide	CO	Cryogen	630-08-0	UGFAIRIUMAVXCW-UHFFFAOYSA-N	[26]
Chlorobenzene	Chlorobenzene	C_6_H_5_Cl	Halocb	108-90-7	MVPPADPHJFYWMZ-UHFFFAOYSA-N	[37]
Cyclohexane	Cyclohexane	C_6_H_12_	Naphthene	110-82-7	XDTMQSROBMDMFD-UHFFFAOYSA-N	[26]
Cyclopentane	Cyclopentane	C_5_H_10_	Naphthene	287-92-3	RGSFGYAAUTVSQA-UHFFFAOYSA-N	[24]
D4	Octamethylcyclotetrasiloxane	C_8_H_24_O_4_Si_4_	Siloxane	556-67-2	HMMGMWAXVFQUOA-UHFFFAOYSA-N	[24]
D5	Decamethylcyclopentasiloxane	C_10_H_30_O_5_Si_5_	Siloxane	541-02-6	XMSXQFUHVRWGNA-UHFFFAOYSA-N	[24]
DEA	Diethanolamine	C_4_H_11_NO_2_	Amine	111-42-2	ZBCBWPMODOFKDW-UHFFFAOYSA-N	[37]
Decane	*n*-Decane	C_10_H_22_	*n*-Alkane	124-18-5	DIOQZVSQGTUSAI-UHFFFAOYSA-N	[26]
Deuterium	Deuterium	D_2_	Cryogen	7782-39-0	UFHFLCQGNIYNRP-VVKOMZTBSA-N	[26]
Dichloroethane	1,2-Dichloroethane	C_2_H_4_Cl_2_	Halocb	107-06-2	WSLDOOZREJYCGB-UHFFFAOYSA-N	[37]
Diethyl ether	Diethyl ether	C_4_H_10_O	Ether	60-29-7	RTZKZFJDLAIYFH-UHFFFAOYSA-N	[24]
Dimethyl carbonate (DMC)	Dimethyl ester carbonic acid	C_3_H_6_O_3_	Other	616-38-6	IEJIGPNLZYLLBP-UHFFFAOYSA-N	[24]
Dimethyl ether	Methoxymethane	C_2_H_6_O	Ether	115-10-6	LCGLNKUTAGEVQW-UHFFFAOYSA-N	[26]
Docosane	*n*-Docosane	C_22_H_46_	*n*-Alkane	629-97-0	HOWGUJZVBDQJKV-UHFFFAOYSA-N	[37]
Dodecane	*n*-Dodecane	C_12_H_26_	*n*-Alkane	112-40-3	SNRUBQQJIBEYMU-UHFFFAOYSA-N	[26]
Ethane	Ethane	C_2_H_6_	*n*-Alkane	74-84-0	OTMSDBZUPAUEDD-UHFFFAOYSA-N	[26]
Ethanol	Ethyl alcohol	C_2_H_6_O	Alcohol	64-17-5	LFQSCWFLJHTTHZ-UHFFFAOYSA-N	[27]
Ethylene glycol	1,2-Ethandiol	C_2_H_6_O_2_	Glycol	107-21-1	LYCAIKOWRPUZTN-UHFFFAOYSA-N	[37]
Ethylbenzene	Phenylethane	C_8_H_10_	Aromatic	100-41-4	YNQLUTRBYVCPMQ-UHFFFAOYSA-N	[24]
Heavy water	Deuterium oxide	D_2_O	Water	7789-20-0	XLYOFNOQVPJJNP-ZSJDYOACSA-N	[38]
Helium	Helium-4	He	Cryogen	7440-59-7	SWQJXJOGLNCZEY-UHFFFAOYSA-N	[26]
Heptane	*n*-Heptane	C_7_H_16_	*n*-Alkane	142-82-5	IMNFDUFMRHMDMM-UHFFFAOYSA-N	[26]
Hexadecane	*n*-Hexadecane	C_16_H_34_	*n*-Alkane	544-76-3	DCAYPVUWAIABOU-UHFFFAOYSA-N	[37]
Hexane	*n*-Hexane	C_6_H_14_	*n*-Alkane	110-54-3	VLKZOEOYAKHREP-UHFFFAOYSA-N	[26]
Hydrogen (normal)	Hydrogen (normal)	H_2_	Cryogen	1333-74-0	UFHFLCQGNIYNRP-UHFFFAOYSA-N	[26]
Isooctane	2,2,4-Trimethylpentane	C_8_H_18_	*br*-Alkane	540-84-1	NHTMVDHEPJAVLT-UHFFFAOYSA-N	[24]
Krypton	Krypton	Kr	Cryogen	7439-90-9	DNNSSWSSYDEUBZ-UHFFFAOYSA-N	[26]
MD2M	Decamethyltetrasiloxane	C_10_H_30_Si_4_O_3_	Siloxane	141-62-8	YFCGDEUVHLPRCZ-UHFFFAOYSA-N	[24]
MD3M	Dodecamethylpentasiloxane	C_12_H_36_Si_5_O_4_	Siloxane	141-63-9	FBZANXDWQAVSTQ-UHFFFAOYSA-N	[24]
MD4M	Tetradecamethylhexasiloxane	C_14_H_42_O_5_Si_6_	Siloxane	107-52-8	ADANNTOYRVPQLJ-UHFFFAOYSA-N	[24]
Monoethanolamine (MEA)	Ethanolamine	C_2_H_7_NO	Amine	141-43-5	HZAXFHJVJLSVMW-UHFFFAOYSA-N	[37]
Methane	Methane	CH_4_	*n*-Alkane	74-82-8	VNWKTOKETHGBQD-UHFFFAOYSA-N	[26]
Methanol	Methanol	CH_4_O	Alcohol	67-56-1	OKKJLVBELUTLKV-UHFFFAOYSA-N	[26]
Methyl palmitate	Methyl hexadecanoate	C_17_H_34_O_2_	FAME	112-39-0	FLIACVVOZYBSBS-UHFFFAOYSA-N	[24]
Methylcyclohexane	Methylcyclohexane	C_7_H_14_	Naphthene	108-87-2	UAEPNZWRGJTJPN-UHFFFAOYSA-N	[24]
m-Xylene	1,3-Dimethylbenzene	C_8_H_10_	Aromatic	108-38-3	IVSZLXZYQVIEFR-UHFFFAOYSA-N	[24]
Neon	Neon	Ne	Cryogen	7440-01-9	GKAOGPIIYCISHV-UHFFFAOYSA-N	[26]
Nitrogen	Nitrogen	N_2_	Cryogen	7727-37-9	IJGRMHOSHXDMSA-UHFFFAOYSA-N	[26]
Nonane	*n*-Nonane	C_9_H_20_	*n*-Alkane	111-84-2	BKIMMITUMNQMOS-UHFFFAOYSA-N	[26]
Octane	*n*-Octane	C_8_H_18_	*n*-Alkane	111-65-9	TVMXDCGIABBOFY-UHFFFAOYSA-N	[26]
Oxygen	Oxygen	O_2_	Cryogen	7782-44-7	MYMOFIZGZYHOMD-UHFFFAOYSA-N	[26]
o-Xylene	1,2-Dimethylbenzene	C_8_H_10_	Aromatic	95-47-6	CTQNGGLPUBDAKN-UHFFFAOYSA-N	[24]
Pentane	*n*-Pentane	C_5_H_12_	*n*-Alkane	109-66-0	OFBQJSOFQDEBGM-UHFFFAOYSA-N	[26]
Propane	Propane	C_3_H_8_	*n*-Alkane	74-98-6	ATUOYWHBWRKTHZ-UHFFFAOYSA-N	[26]
Propylene	Propene	C_3_H_6_	*n*-Alkene	115-07-1	QQONPFPTGQHPMA-UHFFFAOYSA-N	[26]
p-Xylene	1,4-Dimethylbenzene	C_8_H_10_	Aromatic	106-42-3	URLKBWYHVLBVBO-UHFFFAOYSA-N	[24]
R1123	Trifluoroethylene	C_2_HF_3_	Halocb	359-11-5	MIZLGWKEZAPEFJ-UHFFFAOYSA-N	[37]
R115	Chloropentafluoroethane	C_2_ClF_5_	Halocb	76-15-3	RFCAUADVODFSLZ-UHFFFAOYSA-N	[26]
R1234yf	2,3,3,3-Tetrafluoroprop-1-ene	C_3_F_4_H_2_	Halocb	754-12-1	FXRLMCRCYDHQFW-UHFFFAOYSA-N	[26]
R1234ze(E)	*trans*-1,3,3,3-Tetrafluoropropene	C_3_F_4_H_2_	Halocb	29,118-24-9	CDOOAUSHHFGWSA-OWOJBTEDSA-N	[24]
R125	Pentafluoroethane	C_2_HF_5_	Halocb	354-33-6	GTLACDSXYULKMZ-UHFFFAOYSA-N	[26]
R134a	1,1,1,2-Tetrafluoroethane	C_2_H_2_F_4_	Halocb	811-97-2	LVGUZGTVOIAKKC-UHFFFAOYSA-N	[26]
R143a	1,1,1-Trifluoroethane	C_2_H_3_F_3_	Halocb	420-46-2	UJPMYEOUBPIPHQ-UHFFFAOYSA-N	[24]
R152a	1,1-Difluoroethane	C_2_H_4_F_2_	Halocb	75-37-6	NPNPZTNLOVBDOC-UHFFFAOYSA-N	[26]
R22	Chlorodifluoromethane	CHClF_2_	Halocb	75-45-6	VOPWNXZWBYDODV-UHFFFAOYSA-N	[26]
R227ea	1,1,1,2,3,3,3-Heptafluoropropane	C_3_HF_7_	Halocb	431-89-0	YFMFNYKEUDLDTL-UHFFFAOYSA-N	[26]
R32	Difluoromethane	CH_2_F_2_	Halocb	75-10-5	RWRIWBAIICGTTQ-UHFFFAOYSA-N	[26]
RC318	Octafluorocyclobutane	C_4_F_8_	Halocb	115-25-3	BCCOBQSFUDVTJQ-UHFFFAOYSA-N	[26]
Toluene	Methylbenzene	C_7_H_8_	Aromatic	108-88-3	YXFVVABEGXRONW-UHFFFAOYSA-N	[26]
Water	Water	H_2_O	Water	7732-18-5	XLYOFNOQVPJJNP-UHFFFAOYSA-N	[39]

**Table 2:** Summary of surface tension binary mixture data

Reference code	Method		Unc. (mN·m^−1^)	Fluid 1	Fluid 2	Npts	*T* range (K)	*x*_1_ range
1974 jai sin 0 [40]	DROPW		0.6–0.7	Ethylbenzene	Cyclohexane	28	298–308	0.0–1.0
2014 pra cow 0 [41]	DROPSH		0.4	Ethylbenzene	Hexadecane	9	294	0.0–1.0
1978 dhi mah 0 [42]	CAPRISE		0.7–0.8	*p*-Xylene	Chlorobenzene	18	293–303	0.1–0.9
1972 mah cho 0 [43]	CAPRISE		0.1–0.2	*p*-Xylene	Pentane	7	288	0.14–0.82
2010 dom ril 0 [44]	DROPV		0.1–0.2	*p*-Xylene	Hexane	16	298	0.12–0.95
1974 jai sin 0 [40]	DROPW		0.6–0.7	*p*-Xylene	Cyclohexane	28	298–308	0.0–1.0
2013 gay cas 0 [45]	DROPV		0.2–0.3	*p*-Xylene	Octane	12	308	0.05–0.95
2009 mos cas 0 [46]	DROPV		0.3	*p*-Xylene	Decane	11	298	0.10–0.95
2013 gay cas 0 [45]	DROPV		0.3	*p*-Xylene	Decane	11	308	0.10–0.95
2004 ouy lu 3 [47]	DROPSH		0.3	*p*-Xylene	Ethanol	11	298	0.0–1.0
1992 wan nar 1 [48]	CAPRISE		0.6–0.7	*p*-Xylene	Methanol	44	293–318	0.0–1.0
2004 ouy yan 0 [49]	DROPSH		0.3	*p*-Xylene	Acetone	9	298	0.10–0.90
2013 gay cas 0 [45]	DROPV		0.3	*p*-Xylene	DMC	10	308	0.06–0.95
1978 cal mcl 0 [50]	CAPRISE		0.1	Butane	RC318	24	234–254	0.0–1.0
1985 hsu nag 0 [51]	DROPSH		0.1–0.2	Butane	Carbon dioxide	42	319–378	0.15–0.91
2005 goz dan 0 [52]	OTHER		0.02	Butane	methane	1	311	0.49
1914 wor & 1 [53]	UNKN		0.5–0.6	Dichloroethane	Benzene	13	286–343	0.0–1.0
1978 dhi mah 0 [42]	CAPRISE		0.7–0.8	*m*-Xylene	Chlorobenzene	18	293–303	0.1–0.9
1972 mah cho 0 [43]	CAPRISE		0.1–0.2	*m*-Xylene	Pentane	9	288	0.12–0.89
2017 tah & 0 [54]	DROPSH		0.3–0.4	*m*-Xylene	Pentane	11	293	0.0–1.0
2006 dom seg 0 [55]	DROPV		0.1–0.2	*m*-Xylene	Hexane	18	298	0.04–0.95
2017 tah & 0 [54]	DROPSH		0.3–0.4	*m*-Xylene	Hexane	11	293	0.0–1.0
1974 jai sin 0 [40]	DROPW		0.6–0.7	*m*-Xylene	Cyclohexane	28	298–308	0.0–1.0
2017 tah & 0 [54]	DROPSH		0.3–0.4	*m*-Xylene	Octane	11	293	0.0–1.0
2017 tah & 0 [54]	DROPSH		0.3–0.4	*m*-Xylene	Heptane	11	293	0.0–1.0
2004 ouy lu 3 [47]	DROPSH		0.3	*m*-Xylene	Ethanol	11	298	0.0–1.0
2004 ouy yan 0 [49]	DROPSH		0.3	*M*-Xylene	Acetone	9	298	0.10–0.90
1929 ham and 0 [56]	CAPRISE		0.2	*m*-Xylene	Benzene	5	298	0.40–1.0
1917 mor gri 0 [57]	DROPW		0.2	Toluene	Chlorobenzene	6	283–313	0.26–0.81
1972 mah cho 0 [43]	CAPRISE		0.1	Toluene	Pentane	8	288	0.18–0.79
1970 lam ben 0 [58]	BUBBLEP		0.7	Toluene	Cyclohexane	11	298	0.11–0.90
1958 lin van 1 [59]	OTHER		8–10	Toluene	Octane	17	303–398	0.0–1.0
2021 vak alw 0 [60]	RINGTE		0.5	Toluene	Nonane	44	298–313	0.0–1.0
2003 kah wad 0 [61]	DROPSH		0.2–0.3	Toluene	Heptane	34	288–328	0.0–1.0
1970 lam ben 0 [58]	BUBBLEP		0.6–0.7	Toluene	Cyclopentane	10	298	0.10–0.89
2014 pra cow 0 [41]	DROPSH		0.4	Toluene	Hexadecane	8	294	0.0–1.0
2021 vak alw 0 [60]	RINGTE		0.5	Toluene	Hexadecane	44	298–313	0.0–1.0
1974 mye cle 0 [62]	BUBBLEP		0.3	Toluene	Ethanol	10	303	0.0–1.0
1993 sha muk 0 [63]	DROPW		0.8–1.1	Toluene	Ethanol	5	298	0.1–0.9
1982 sin lar 0 [64]	CAPRISE		0.1	Toluene	Methanol	11	308	0.0–1.0
1992 wan nar 1 [48]	CAPRISE		0.5–0.7	Toluene	Methanol	44	293–318	0.0–1.0
2003 kah wad 1 [65]	DROPSH		0.2–0.3	Toluene	Acetone	55	288–328	0.0–1.0
2007 end kah 0 [66]	DROPSH		0.5–0.6	Toluene	Acetone	55	288–328	0.0–1.0
1917 mor gri 0 [57]	DROPW		0.3–0.6	Toluene	Benzene	6	284–313	0.22–0.72
1970 kon lya 1 [67]	BUBBLEP		0.3–0.4	Toluene	Benzene	21	293–333	0.0–1.0
1972 mah cho 0 [43]	CAPRISE		0.1–0.2	Chlorobenzene	Pentane	7	288	0.18–0.84
1978 dhi mah 0 [42]	CAPRISE		0.6–0.8	Chlorobenzene	Cyclohexane	18	293–303	0.1–0.9
1917 mor gri 0 [57]	DROPW		0.2	Chlorobenzene	Acetone	1	288	0.34
1917 mor gri 0 [57]	DROPW		0.4–0.6	Chlorobenzene	Benzene	6	283–313	0.21–0.62
1978 dhi mah 0 [42]	CAPRISE		0.7–0.8	Chlorobenzene	Benzene	17	293–303	0.1–0.9
1978 dhi mah 0 [42]	CAPRISE		0.7–0.8	Chlorobenzene	*o*-Xylene	18	293–303	0.1–0.9
1972 mah cho 0 [43]	CAPRISE		0.1–0.2	Pentane	Cyclohexane	7	288	0.15–0.81
1992 abd ada 0 [68]	CAPRISE		0.1–1.2	Pentane	Heptane	61	303–538	0.0–1.0
2010 moh ras 0 [69]	BUBBLEP		0.2	Pentane	Heptane	38	293–323	0.17–0.97
2011 moh & 0 [70]	CAPRISE		0.1–0.2	Pentane	Hexadecane	35	293–323	0.2–0.9
1972 mah cho 0 [43]	CAPRISE		0.1–0.2	Pentane	Benzene	7	288	0.09–0.81
2018 sat coo 0 [71]	DROPSH		0.1–0.2	Pentane	Methane	7	313	0.50–0.95
1963 cle cha 0 [72]	BUBBLEP		0.2–0.3	Hexane	Cyclohexane	19	298–308	0.0–1.0
1967 rid but 0 [73]	RINGTE		0.6–0.7	Hexane	Cyclohexane	8	293	0.0–1.0
1968 sch cle 1 [74]	BUBBLEP		0.5–0.7	Hexane	Dodecane	21	298–313	0.0–1.0
2019 kol yan 0 [75]	SLS		0.1–0.2	Hexane	Carbon dioxide	5	303	0.25–1.0
1994 pap pan 1 [76]	CAPRISE		0.1	Hexane	Ethanol	20	298	0.0–1.0
2000 jim cas 0 [77]	DROPV		0.1	Hexane	Ethanol	17	298	0.04–0.93
2007 gin vil 1 [78]	DROPV		0.3	Hexane	Ethanol	77	283–313	0.07–0.91
1935 tri & 0 [79]	UNKN		0.1	Hexane	Methanol	4	295	0.0–1.0
1970 ram pat 0 [80]	UNKN		0.5–0.7	Hexane	Methanol	22	303–318	0.0–1.0
1966 sch ran 0 [81]	BUBBLEP		0.5–0.6	Hexane	Benzene	23	298–313	0.0–1.0
1967 rid but 0 [73]	RINGTE		0.6–0.8	Hexane	Benzene	8	293	0.0–1.0
2002 gom mej 0 [82]	DROPW		0.6	Cyclohexane	Decane	6	298	0.0–1.0
2003 kah wad 1 [65]	DROPSH		0.2–0.3	Cyclohexane	Heptane	55	288–328	0.0–1.0
2001 gom mej 0 [83]	RINGTE		0.4–0.5	Cyclohexane	Isooctane	126	298–323	0.0–1.0
1935 tri & 0 [79]	UNKN		0.1	Cyclohexane	Ethanol	6	295	0.0–1.0
1974 mye cle 0 [62]	BUBBLEP		0.3	Cyclohexane	Ethanol	11	303	0.0–1.0
2003 kah wad 1 [65]	DROPSH		0.2–0.3	Cyclohexane	Acetone	44	288–318	0.0–1.0
2008 mej seg 1 [84]	BUBBLEP		0.3	Cyclohexane	Acetone	10	303	0.06–0.95
1929 ham and 0 [56]	CAPRISE		0.2	Cyclohexane	Benzene	5	298	0.30–0.62
1967 rid but 0 [73]	RINGTE		0.7–0.8	Cyclohexane	Benzene	9	293	0.0–1.0
1968 sur ram 0 [85]	CAPRISE		0.1	Cyclohexane	Benzene	15	293–303	0.09–0.87
1970 kon lya 1 [67]	BUBBLEP		0.3–0.4	Cyclohexane	Benzene	39	293–333	0.0–1.0
1970 lam ben 0 [58]	BUBBLEP		0.6–0.8	Cyclohexane	Benzene	28	293–303	0.10–0.89
1974 jai sin 0 [40]	DROPW		0.6–0.8	Cyclohexane	*o*-Xylene	28	298–308	0.0–1.0
2019 abr bag 0 [86]	RINGTE		0.5–0.7	DEA	Ethanol	13	313	0.0–1.0
2019 abr bag 0 [86]	RINGTE		0.5–0.7	DEA	Methanol	14	313	0.0–1.0
1994 rin oel 0 [87]	RINGTE		1.5–1.7	DEA	Water	12	293–353	0.02–0.07
1996 vaz alv 0 [88]	OTHER		0.3–0.5	DEA	Water	66	298–323	0.0–1.0
1998 alv ren 0 [89]	DROPW		0.8	DEA	Water	6	298–323	0.15
2001 agu tre 0 [90]	DROPSH		1.3–1.5	DEA	Water	21	293–363	0.02–0.07
2003 alv can 0 [91]	DROPSH		0.5	DEA	Water	6	298–323	0.15
2014 fu du 0 [92]	OTHER		2.0	DEA	Water	12	293–323	0.04–0.07
2018 dey das 0 [93]	DROPSH		0.8	DEA	Water	9	313–333	0.02–0.07
2018 fu xie 0 [94]	OTHER		0.6	DEA	Water	15	303–323	0.0–1.0
2018 sho & 0 [95]	RINGTE		0.6–0.9	DEA	Water	65	298–348	0.0–1.0
2015 lop igl 0 [96]	DROPV		0.1	Octane	Isooctane	55	293–313	0.0–1.0
2003 seg del 0 [97]	DROPV		0.2	Octane	Ethanol	17	298	0.07–0.92
2011 mej car 0 [98]	BUBBLEP		0.3	Octane	Ethanol	31	298–318	0.04–0.90
2016 and mar 0 [99]	DROPSH		0.3	Octane	*o*-Xylene	11	298	0.05–0.95
2013 gay cas 0 [45]	DROPV		0.2–0.3	Octane	DMC	12	308	0.05–0.95
2021 vak alw 0 [60]	RINGTE		0.4–0.5	Nonane	Benzene	44	298–313	0.0–1.0
2016 and mar 0 [99]	DROPSH		0.3–0.4	Nonane	*o*-Xylene	11	298	0.05–0.95
2020 ond sar 0 [100]	DROPV		0.3	Methyl palmitate	Ethanol	1	298	0.04
1964 eva cle 0 [101]	BUBBLEP		0.2–0.3	Dodecane	Isooctane	9	303	0.0–1.0
2022 yan wu 0 [102]	DROPSH		0.2–0.4	Dodecane	Hexadecane	36	298–573	0.31–0.80
2011 mej car 0 [98]	BUBBLEP		0.3	Dodecane	Ethanol	22	298–303	0.05–0.95
1966 sch ran 0 [81]	BUBBLEP		0.5–0.6	Dodecane	Benzene	23	298–313	0.0–1.0
2018 pra mun 0 [103]	DROPSH		0.3	Dodecane	Methylcyclohexane	9	293	0.1–0.9
2010 bi li 0 [104]	CAPRISE		0.4	Dimethyl ether	Propane	114	243–333	0.29–0.69
1986 nag rob 0 [105]	DROPW		0.1–0.7	Decane	Carbon dioxide	41	344–378	0.10–0.51
2001 sha rob 0 [106]	DROPSH		0.1–0.7	Decane	Carbon dioxide	23	344	0.10–0.89
2002 rol cac 0 [107]	RINGTE		0.3–0.4	Decane	Heptane	25	293–333	0.0–1.0
2002 gom mej 0 [82]	DROPW		0.6	Decane	Isooctane	6	298	0.0–1.0
2002 rol cac 0 [107]	RINGTE		0.4	Decane	Hexadecane	25	293–333	0.0–1.0
2005 que cac 0 [108]	OTHER		0.3–0.4	Decane	Docosane	19	313–343	0.2–0.8
2011 mej car 0 [98]	BUBBLEP		0.3	Decane	Ethanol	32	303–318	0.02–0.97
2016 and mar 0 [99]	DROPSH		0.4	Decane	*o*-Xylene	11	298	0.05–0.95
2013 gay cas 0 [45]	DROPV		0.3	Decane	DMC	11	308	0.05–0.95
1964 gri rud 0 [109]	CAPRISE		0.1	Hydrogen	Deuterium	67	16–20	0.30–0.96
1967 bla kro 0 [110]	CAPRISE		0.1	Hydrogen	Argon	21	87–140	0.0–0.05
2019 abr bag 0 [86]	RINGTE		0.5–0.7	MEA	Ethanol	14	313	0.0–1.0
2020 abr bag 0 [111]	RINGTE		0.5–0.7	MEA	Ethanol	12	303	0.27–0.98
2019 abr bag 0 [86]	RINGTE		0.5–0.7	MEA	Methanol	12	313	0.0–1.0
2020 abr bag 0 [111]	RINGTE		0.5–0.7	MEA	Methanol	10	303	0.28–0.99
1981 ano & 5 [112]	CAPRISE		0.9–1.1	MEA	Water	20	303–393	0.03–0.05
1997 vaz alv 0 [113]	OTHER		0.4–0.6	MEA	Water	83	298–323	0.0–1.0
1998 alv ren 0 [89]	DROPW		1.5–1.6	MEA	Water	5	298–323	0.23
2012 han jin 0 [114]	DROPSH		0.6–0.8	MEA	Water	44	303–333	0.0–1.0
2013 jay jay 0 [115]	DROPV		1.8	MEA	Water	4	313–343	0.54
2013 jay wee 0 [116]	DROPV		2.5–2.9	MEA	Water	24	303–333	0.07–0.41
2014 fu du 0 [92]	OTHER		2.0	MEA	Water	12	293–323	0.07–0.11
2018 fu xie 0 [94]	OTHER		0.6	MEA	Water	15	303–323	0.0–1.0
2018 sho & 0 [95]	RINGTE		0.6–0.9	MEA	Water	66	298–348	0.0–1.0
2014 lun cow 0 [117]	DROPSH		0.3–0.4	Heptane	Isooctane	4	294	0.0–1.0
2015 lop igl 0 [96]	DROPV		0.1	Heptane	Isooctane	55	293–313	0.0–1.0
1958 koe vil 0 [118]	CAPRISE		0.1–0.7	Heptane	Hexadecane	6	293–303	0.0–1.0
2002 rol cac 0 [107]	RINGTE		0.3–0.4	Heptane	Hexadecane	25	293–333	0.0–1.0
2011 moh & 0 [70]	CAPRISE		0.2	Heptane	Hexadecane	35	293–323	0.2–0.9
2003 que sil 0 [119]	RINGTE		0.6–0.8	Heptane	Docosane	12	313–343	0.25–0.75
1994 pap pan 1 [76]	CAPRISE		0.1	Heptane	Ethanol	22	298	0.0–1.0
2016 yue liu 0 [120]	OTHER		0.2	Heptane	Ethanol	66	293–318	0.0–1.0
1970 kon lya 1 [67]	BUBBLEP		0.3–0.4	Heptane	Benzene	27	293–333	0.0–1.0
1993 zho zhu 0 [121]	BUBBLEP		0.5–0.7	Heptane	Benzene	20	293–303	0.08–0.90
1970 lam ben 0 [58]	BUBBLEP		0.6–0.7	Cyclopentane	Benzene	9	298	0.11–0.90
1996 hei sch 0 [122]	CAPRISE		0.-0.2	R125	R143a	21	223–333	0.28–0.79
1999 oka shi 0 [123]	CAPRISE		0.2	R125	R143a	7	273–303	0.41
2001 fro wil 1 [124]	OTHER		0.2	R125	R143a	10	243–333	0.41
1996 hei sch 0 [122]	CAPRISE		0–0.2	R125	R32	8	223–333	0.27–0.77
1999 oka shi 0 [123]	CAPRISE		0.2	R125	R32	18	273–313	0.31–0.35
2003 dua lin 0 [125]	CAPRISE		0.2	R125	R32	236	253–333	0.18–0.58
1996 hei sch 0 [122]	CAPRISE		0.1–0.2	R125	R152a	21	223–333	0.16–0.69
2009 bi zha 1 [126]	CAPRISE		0.4	R125	R152a	54	243–328	0.06–0.19
1996 hei sch 0 [122]	CAPRISE		0–0.2	R125	R134a	21	223–333	0.24–0.75
1996 hei sch 0 [122]	CAPRISE		0.1–0.2	R143a	R134a	21	223–333	0.23–0.72
2004 lin dua 0 [127]	CAPRISE		0.2	R143a	R134a	105	257–329	0.29–0.79
2003 lin dua 2 [128]	CAPRISE		0.1	R143a	R227ea	241	253–333	0.39–0.85
2016 yue liu 0 [120]	OTHER		0.2	Isooctane	Ethanol	44	288–318	0.0–1.0
1964 eva cle 0 [101]	BUBBLEP		0.2–0.3	Isooctane	Benzene	9	303	0.0–1.0
2015 zha li 3 [129]	DROPV		0.2	Isooctane	Methylcyclohexane	44	293–308	0.0–1.0
2021 vak alw 0 [60]	RINGTE		0.5	Hexadecane	Benzene	44	298–313	0.0–1.0
2018 pra mun 0 [103]	DROPSH		0.3	Hexadecane	Methylcyclohexane	9	293	0.1–0.90
1969 mye cle 0 [130]	BUBBLEP		0.2–0.3	Hexadecane	D4	9	303	0.0–1.0
1929 ham and 0 [56]	CAPRISE		0.1–0.2	Diethyl ether	Benzene	4	298	0.24–1.0
1965 spr pra 1 [131]	CAPRISE		0.2	Carbon monoxide	Nitrogen	10	84	0.0–1.0
1970 kon lya 1 [67]	BUBBLEP		0.3	Ethanol	Methanol	39	293–333	0.0–1.0
1929 ham and 0 [56]	CAPRISE		0.1	Ethanol	Acetone	5	298	0.0–1.0
1902 ram ast 0 [132]	CAPRISE		0.2–0.8	Ethanol	Benzene	39	283–351	0.0–1.0
1907 rit & 0 [133]	UNKN		0.1	Ethanol	Benzene	5	298	0.0–1.0
1917 mor sca 0 [134]	UNKN		0.1–0.2	Ethanol	Benzene	13	298–318	0.0–1.0
1929 ham and 0 [56]	CAPRISE		0.1–0.2	Ethanol	Benzene	4	298	0.42–1.0
1935 tri & 0 [79]	UNKN		0.1	Ethanol	Benzene	8	295	0.0–1.0
1974 mye cle 0 [62]	BUBBLEP		0.3	Ethanol	Benzene	7	303	0.0–1.0
1885 tra & 0 [135]	UNKN		0.1	Ethanol	Water	7	288	0.01–1.0
1903 des & 0 [136]	UNKN		0.2–0.7	Ethanol	Water	11	288	0.0–1.0
1913 mor nei 0 [137]	DROPW		0.1–0.5	Ethanol	Water	36	273–303	0.0–1.0
1922 bir & 0 [138]	DROPW		0.3–0.6	Ethanol	Water	15	298	0.0–1.0
1936 ern wat 0 [139]	CAPRISE		0.1–0.2	Ethanol	Water	9	298	0.04–0.78
1937 val hoh 0 [140]	UNKN		0.1–0.5	Ethanol	Water	44	293–323	0.0–0.88
1940 bon bym [141]	CAPRISE		0.2–0.5	Ethanol	Water	42	293–362	0.01–0.83
1950 sta guy 0 [142]	RINGTE		1	Ethanol	Water	11	298	0.0–0.85
1951 tei gor 0 [143]	BUBBLEP		0.2–0.8	Ethanol	Water	200	263–333	0.0–1.0
1968 efr & 1 [144]	BUBBLEP		0.5–1.4	Ethanol	Water	54	283–333	0.02–1.0
1986 wan jey 0 [145]	RINGTE		0.5–1.2	Ethanol	Water	5	303	0.0–1.0
1988 kal bid 0 [146]	BUBBLEP		0.2–0.4	Ethanol	Water	12	351–369	0.01–0.90
1995 vaz alv 0 [147]	RINGTE		0.3–0.9	Ethanol	Water	98	293–323	0.0–1.0
2005 bel her 0 [148]	DROPW		0.2–0.3	Ethanol	Water	11	298	0.0–1.0
2009 max & 0 [149]	DROPV		0.2–0.3	Ethanol	Water	13	298	0.0–1.0
2016 lud kus 0 [150]	BUBBLEP		0.3	Ethanol	Water	1	293	0.04
2018 gon pal 0 [151]	RINGTE		2	Ethanol	Water	6	298	0.27–0.60
2019 raz hal 0 [152]	RINGTE		0.5–0.9	Ethanol	Water	10	298–313	0.0–0.09
2020 gon pan 0 [153]	OTHER		0.4	Ethanol	Water	4	298	0.28–0.61
2020 kho rah 0 [154]	DROPSH		0.8	Ethanol	Water	10	298	0.0–0.002
2021 gom nav [155]	OTHER		0.3	Ethanol	Water	16	293–323	0.04–0.16
2012 bag ami 0 [156]	RINGTE		0.5–1.0	Ethanol	Heavy water	84	288–318	0.001–0.86
2004 ouy lu 3 [47]	DROPSH		0.3	Ethanol	*o*-Xylene	11	298	0.0–1.0
1974 mye cle 0 [62]	BUBBLEP		0.3	Ethanol	Methylcyclohexane	10	303	0.0–1.0
2003 azi hem 0 [157]	RINGTE		0.3–0.7	Ethanol	Ethylene glycol	56	293–323	0.0–1.0
1973 cam kar 0 [158]	UNKN		0.7	Methanol	Acetone	12	298	0.0–1.0
1982 sin lar 0 [64]	CAPRISE		0.1	Methanol	Acetone	8	308	0.0–1.0
1917 mor sca 0 [134]	UNKN		0.2	Methanol	Benzene	14	273–303	0.0–1.0
1933 sha muk 0 [63]	DROPW		0.9–1.1	Methanol	Benzene	5	298	0.1–0.9
1885 tra & 0 [135]	UNKN		0.1	Methanol	Water	6	288	0.01–1.0
1913 mor nei 0 [137]	DROPW		0.5–0.6	Methanol	Water	26	273–303	0.0–1.0
1937 val hoh 0 [140]	UNKN		0.1–0.4	Methanol	Water	31	291–323	0.05–1.0
1951 tei gor 0 [143]	BUBBLEP		0.2–0.7	Methanol	Water	110	263–323	0.0–1.0
1958 uch mat 0 [159]		OTHER	0.5–1.8	Methanol	Water	76	303–363	0.0–1.0
1968 efr & 1 [144]	CAPRISE		0.5–1.0	Methanol	Water	30	283–333	0.17–1.0
1988 kal bid 0 [146]	BUBBLEP		0.3–0.5	Methanol	Water	11	339–356	0.17–0.95
1995 vaz alv 0 [147]	RINGTE		0.3–0.9	Methanol	Water	98	293–323	0.0–1.0
2009 max & 0 [149]	DROPV		0.2–0.3	Methanol	Water	13	298	0.0–1.0
2012 bag ami 0 [156]	RINGTE		0.5–0.9	Methanol	Heavy water	64	288–318	0.00–0.82
1929 ham and 0 [56]	CAPRISE		0.1–0.2	Acetone	Benzene	5	298	0.30–1.0
1970 shi & 1 [160]	CAPRISE		4.7–5.7	Acetone	Benzene	10	298	0.0–1.0
1988 ron lu 0 [161]	BUBBLEP		0.6–0.7	Acetone	Benzene	18	303	0.0–1.0
1917 mor sca 0 [134]	UNKN		0.2–0.3	Acetone	Water	78	273–318	0.0–1.0
1932 ern lit 0 [162]	CAPRISE		0.2	Acetone	Water	9	298	0.03–0.74
1951 tei gan 0 [163]	BUBBLEP		0.2–0.6	Acetone	Water	81	273–313	0.0–1.0
1957 how mca [164]	CAPRISE		0.1–0.4	Acetone	Water	81	288–343	0.0–1.0
1970 kon lya 1 [67]	BUBBLEP		0.3–0.9	Acetone	Water	17	293	0.0–1.0
1976 tor pog 0 [165]	BUBBLEP		0.2–0.5	Acetone	Water	48	298–343	0.0–1.0
1988 ron lu 0 [161]	BUBBLEP		0.6–1.9	Acetone	Water	19	303	0.0–1.0
2007 end kah 0 [66]	DROPSH		0.3–0.7	Acetone	Water	70	288–328	0.0–1.0
2004 ouy yan 0 [49]	DROPSH		0.3	Acetone	*o*-Xylene	8	298	0.10–0.80
1970 lam ben 0 [58]	BUBBLEP		0.8	Benzene	*o*-Xylene	8	298	0.10–0.79
2013 bai kav 0 [166]	CAPRISE		0.1–1.7	Methane	Ethane	70	93–283	0.0–1.0
2017 sen hug 0 [167]	CAPRISE		0.7	Methane	Propane	27	272–303	0.0–0.55
1960 bla & 0 [168]	CAPRISE		0.3–0.5	Methane	Nitrogen	33	76–90	0.29–0.91
1965 spr pra 1 [131]	CAPRISE		0.2–0.5	Methane	Nitrogen	12	91	0.0–1.0
1966 fuk bel 0 [169]	CAPRISE		0.1–0.6	Methane	Krypton	35	110–118	0.18–0.67
1960 bla & 0 [168]	CAPRISE		0.4–0.6	Methane	Argon	28	84–111	0.29–1.0
2009 tan hig 2 [170]	CAPRISE		0.4	Propane	R32	99	280–300	0.00–1.00
2010 zha bi 0 [171]	CAPRISE		0.2	Propane	R152a	51	248–328	0.27–0.59
1996 hei sch 0 [122]	CAPRISE		0.1–0.2	R32	R134a	17	223–333	0.23–0.72
2003 yua hon 0 [172]	CAPRISE		0.2	R32	R134a	300	254–334	0.35–0.86
2021 liu kon 0 [173]	CAPRISE		0.2–0.3	R32	R1123	37	266–307	0.48–0.87
2005 lin dua 0 [174]	CAPRISE		0.3	R32	R227ea	412	252–334	0.54–0.90
2016 cui bi 0 [175]	SLS		0.1	R32	R1234yf	24	293–348	0.52–1.0
2021 liu kon 0 [173]	CAPRISE		0.1–0.2	R32	R1234yf	36	267–333	0.27–0.89
2013 tan hig 0 [176]	CAPRISE		0.6	R32	R1234ze(E)	26	273–323	0.69
2016 cui bi 0 [175]	SLS		0.1	R32	R1234ze(E)	26	293–348	0.30–1.0
1996 hei sch 0 [122]	CAPRISE		0.1–0.2	R152a	R134a	21	223–333	0.24–0.71
1969 ano & 2 [177]	OTHER		1.0	R22	R115	1	298	0.63
1959 bla rud 0 [178]	CAPRISE		0.3–0.6	Nitrogen	Oxygen	34	61–88	0.1–0.9
1994 ost ost 1 [179]	CAPRISE		0.3–0.5	Nitrogen	Oxygen	88	55–78	0.0–1.0
2008 bai kav 0 [180]	CAPRISE		0.1–0.2	Nitrogen	Oxygen	61	80–132	0.0–1.0
2006 kav and 0 [181]	CAPRISE		0.05–1.3	Helium	Argon	33	108–140	0.0–0.01
2006 kav and 0 [181]	CAPRISE		0.05–0.2	Neon	Argon	27	111–140	0.0–0.04
2004 bai kav 0 [182]	CAPRISE		0.1–0.3	Nitrogen	Helium	38	90–118	0.97–1.0
1960 bla & 0 [168]	CAPRISE		0.3–0.4	Nitrogen	Argon	21	69–86	0.02–0.7
1965 spr pra 1 [131]	CAPRISE		0.2–0.3	Nitrogen	Argon	19	84	0.0–1.0
1946 cle & 0 [183]	RINGTE		1	Water	Ethylene glycol	2	298	0.0–1.0
1971 nak mat 0 [184]	CAPRISE		0.3	Water	Ethylene glycol	18	303	0.0–1.0
1981 won chu [185]	RINGTE		1–1.2	Water	Ethylene glycol	4	298	0.8–1.0
1991 hok che 0 [186]	BUBBLEP		0.4–0.6	Water	Ethylene glycol	174	295–471	0.0–0.95
1996 hor fuk 0 [187]	CAPRISE		0.5–0.8	Water	Ethylene glycol	44	253–298	0.0–1.0
1998 tsi mol 0 [188]	RINGTE		1.6–2.4	Water	Ethylene glycol	64	283–323	0.0–1.0
2004 hab hov 0 [189]	RINGTE		1.4–2.0	Water	Ethylene glycol	15	298	0.80–0.99
2008 zha zha 2 [190]	DROPV		1.1–1.4	Water	Ethylene glycol	48	308–323	0.0–1.0
2011 raf bag 0 [191]	RINGTE		0.6–0.9	Water	Ethylene glycol	54	283–308	0.10–0.99
2014 tiw son 0 [192]	OTHER		0.4	Water	Ethylene glycol	4	298	0.88–1.0
1959 bla rud 0 [178]	BUBBLEP		0.4–1.0	Oxygen	Argon	28	69–88	0.19–0.9
1965 saj oku 0 [193]	RINGTE		0.3	Oxygen	Argon	36	79–88	0.0–1.0
2016 bi cui 0 [194]	SLS		0.1	R134a	R1234yf	23	293–363	0.32–0.81
2016 bi cui 0 [194]	SLS		0.1	R134a	R1234ze(E)	9	293–369	0.44
1958 wat van 0 [195]	RINGTE		0.5	D4	MD4M	2	293	0.39–0.78
1958 wat van 0 [195]	RINGTE		0.5	D4	MD2M	3	293	0.3–0.7
1987 nad & 0 [196]	UNKN		0–0.4	Krypton	Argon	60	120–200	0.0–1.0
1994 sul bai 0 [197]	CAPRISE		0.1–0.8	Krypton	Argon	40	120–193	0.0–1.0
1958 wat van 0 [195]	RINGTE		0.5	MD4M	D5	3	293	0.26–0.66
1958 wat van 0 [195]	RINGTE		0.5	MD3M	D5	1	293	0.5
2021 liu kon 0 [173]	CAPRISE		0.2	R1123	R1234yf	39	234–312	0.11–0.73

*BUBBLEP* maximum bubble pressure; *CAPRISE* capillary rise; *DROPSH* pendant drop shape; *DROPV* drop volume; *DROPW* drop weight; *OTHER* other; *RINGTE* ring tensiometer; *SLS* surface light scattering; *UNKN* unknown

## Results

Evaluations were first made with the interaction parameter in Eq. 4 set to zero (*δ*_*ij*_ = 0) for all the mixtures. All properties such as the pure fluid surface tensions and the mixture densities and compositions required in Eqs. 1–4 were obtained using the REFPROP v10 [32] computer program, with additional changes that are detailed in the supplementary information, Appendix A. A second set of evaluations was made after fitting the binary interaction parameter *δ*_*ij*_ to the experimental data with a trust-region reflective least squares algorithm in Python, scipy.optimize.curve_fit [198].^1^ A single binary interaction parameter was fit for each fluid mixture pair, including all data sets for any given pair. For discussion of the results, we define AAPD as the average absolute percentage deviation, where PCTDEV = 100(*σ*_calc_ − *σ*_exp_)/*σ*_exp_, and AAPD = (∑│PCTDEV│)/*n*, and the summation is over all *n* points. AAD is the average absolute deviation, AAD = (∑│*σ*_calc_ − *σ*_exp_ │)/*n*, expressed in mN·m^−1^, and AADMAX is the maximum value of the AAD. We do not include in the statistics any points where the REFPROP program had convergence errors. Since the surface tension is zero at the critical point, some points near the critical region may have unusually large percentage deviations and it is more informative to examine the absolute deviation instead. Detailed results for each data set listed in Table 2 are presented in the supplemental information in Appendix B, Table B1. The data are also provided in the supplemental information. Here we will discuss the results in terms of chemical families.

### Mixtures with *n*-Alkanes

**Fig. 1:**
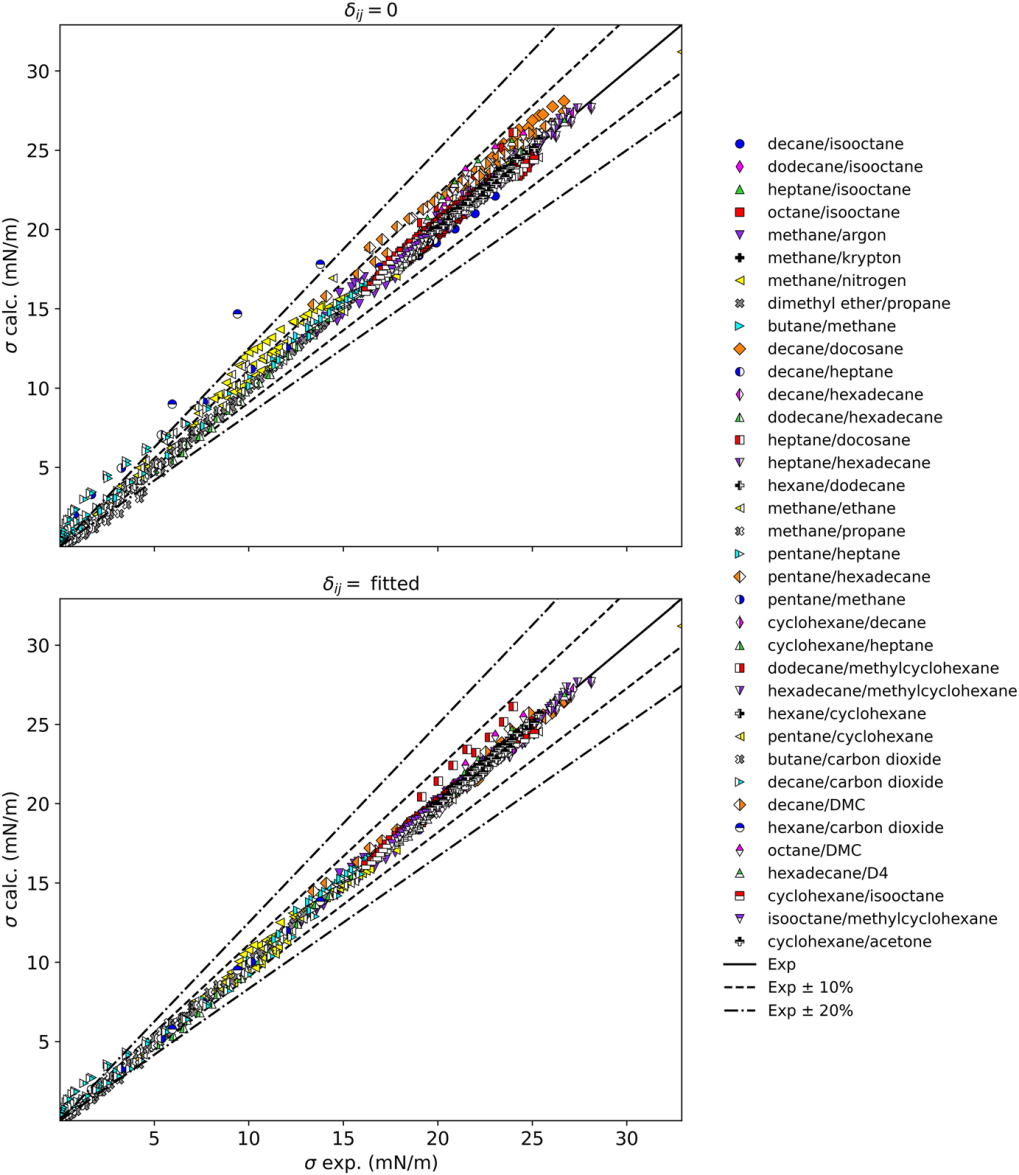
Summary of results for mixtures with alkanes

Table 3 summarizes the results for mixtures with *n*-alkanes, presenting results both for binary interaction parameters set to zero and for fitted binary interaction parameters. Figure 1 displays these results graphically. The mixtures considered in this section contain *n*-alkanes mixed only with nonpolar fluids (branched alkanes, naphthenes, cryogens, and CO_2_) except for four mixtures with polar aprotic fluids dimethyl ether, acetone, dimethyl carbonate, and octamethylcyclotetrasiloxane (D4). Excluded from these results are mixtures of *n*-alkanes with hydrogen bonding fluids, aromatics, or halocarbons; these mixtures are treated separately in later sections. The results in Table 3 are arranged by mixture classes.

**Table 3:** Summary of results for alkane mixtures

Mixture class	Fluids	Npts	*δ*_ij_ = 0			Fitted results			
			AAPD (%)	AAD (mN·m^−1^)	max AD (mN·m^−1^)	AAPD (%)	AAD (mN·m^−1^)	max AD (mN·m^−1^)	*δ*_ij_
*n*-Alkane/*br*-alkane	Decane/isooctane	6	3.68	0.79	0.96	1.37	0.29	0.65	− 0.026
*n*-Alkane/*br*-alkane	Dodecane/isooctane	9	1.22	0.26	0.47	0.16	0.03	0.08	− 0.010
*n*-Alkane/*br*-alkane	Heptane/isooctane	59	1.32	0.24	0.41	0.53	0.10	0.33	− 0.010
*n*-Alkane/*br*-alkane	Octane/isooctane	55	1.41	0.27	0.38	0.46	0.09	0.33	− 0.010
*n*-Alkane/cryogen	Methane/argon	28	2.72	0.42	1.22	2.34	0.36	0.85	0.017
*n*-Alkane/cryogen	Methane/krypton	35	1.30	0.19	0.31	0.35	0.05	0.18	0.008
*n*-Alkane/cryogen	Methane/nitrogen	45	9.71	1.08	2.24	3.68	0.42	0.88	0.067
*n*-Alkane/ether	Propane/dimethyl ether	114	1.37	0.11	0.36	1.38	0.11	0.37	0.001
*n*-Alkane/*n*-alkane	Butane/methane	1	18.18	0.15	0.15	0.00	0.00	0.00	− 0.089
*n*-Alkane/*n*-alkane	Decane/docosane	19	6.23	1.51	2.00	0.68	0.17	0.46	0.036
*n*-Alkane/*n*-alkane	Decane/heptane	25	1.86	0.39	0.61	0.90	0.19	0.61	− 0.012
*n*-Alkane/*n*-alkane	Decane/hexadecane	25	1.10	0.27	0.61	0.86	0.21	0.61	0.005
*n*-Alkane/*n*-alkane	Dodecane/hexadecane	36	3.04	0.40	0.92	2.86	0.28	0.74	0.011
*n*-Alkane/*n*-alkane	Heptane/docosane	12	5.72	1.27	2.13	2.03	0.49	1.70	0.030
*n*-Alkane/*n*-alkane	Heptane/hexadecane	66	2.22	0.50	0.91	0.98	0.22	0.91	0.015
*n*-Alkane/*n*-alkane	Hexane/dodecane	21	0.82	0.18	0.42	0.56	0.12	0.28	0.006
*n*-Alkane/*n*-alkane	Methane/ethane	70	8.05	0.56	2.47	3.29	0.19	1.70	0.074
*n*-Alkane/*n*-alkane	Methane/propane	27	5.69	0.12	0.24	5.67	0.12	0.24	− 0.001
*n*-Alkane/*n*-alkane	Pentane/heptane	99^a^	8.80	0.11	0.67	8.80	0.11	0.67	0.000
*n*-Alkane/*n*-alkane	Pentane/hexadecane	35	8.07	1.58	2.45	1.67	0.31	1.05	0.045
*n*-Alkane/*n*-alkane	Pentane/methane	7	54.34	1.35	1.70	10.43	0.16	0.36	0.227
*n*-Alkane/naphthene	Decane/cyclohexane	6	2.68	0.66	0.70	1.14	0.28	0.68	− 0.017
*n*-Alkane/naphthene	Dodecane/methylcyclohexane	9	2.52	0.62	0.88	0.46	0.11	0.37	− 0.018
*n*-Alkane/naphthene	Heptane/cyclohexane	55	1.44	0.30	0.64	0.56	0.12	0.35	0.011
*n*-Alkane/naphthene	Hexadecane/methylcyclohexane	9	0.67	0.18	0.57	0.72	0.19	0.54	0.002
*n*-Alkane/naphthene	Hexane/cyclohexane	27	0.96	0.20	0.54	0.75	0.16	0.54	0.007
*n*-Alkane/naphthene	Pentane/cyclohexane	7	2.02	0.42	0.62	0.45	0.09	0.16	0.013
*n*-Alkane/other	Butane/carbon dioxide	42^b^	28.99	0.38	1.18	20.98	0.17	0.67	− 0.178
*n*-Alkane/other	Hexane/carbon dioxide	5	28.23	2.62	5.28	1.37	0.15	0.33	0.186
*n*-Alkane/other	Decane/carbon dioxide	64	468.69	0.82	1.97	358.68	0.49	1.08	0.081
*n*-Alkane/other	Decane/DMC	11	3.40	0.78	1.62	2.04	0.47	0.91	0.023
*n*-Alkane/other	Octane/DMC	12	5.22	1.11	2.22	2.41	0.52	1.23	0.036
*n*-Alkane/siloxane	Hexadecane/D4	9	5.39	1.13	2.12	1.90	0.39	0.72	0.045
Naphthene/*br*-alkane	Cyclohexane/isooctane	126	1.38	0.27	0.68	0.60	0.12	0.68	0.010
Naphthene/*br*-alkane	Methylcyclohexane/isooctane	44	0.56	0.12	0.25	0.53	0.11	0.25	0.001
Naphthene/ketone	Cyclohexane/acetone	54	0.94	0.21	0.48	0.83	0.19	0.51	0.002

^a^Six points omitted from statistics due to REFPROP calculation problems

^b^Three points omitted from statistics due to REFPROP calculation problems

**Fig. 2:**
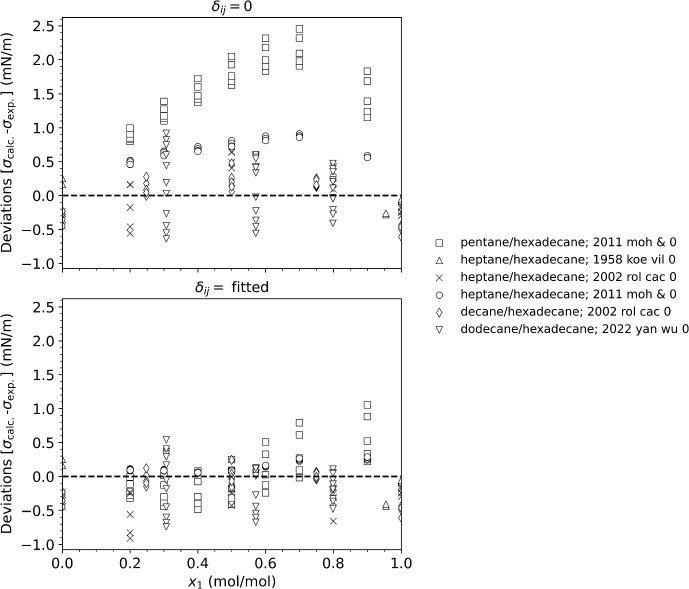
Deviations between the model and experimental data for mixtures of a series of *n*-alkanes with hexadecane

Overall, Fig. 1 and Table 3 show that without the use of binary interaction parameters, non-polar mixtures such as linear and branched alkanes, and linear and branched alkanes with naphthenes have average absolute percentage deviations of about 3 % or less. The propane/dimethyl ether mixture and the cyclohexane/acetone mixture also are represented very well without an interaction parameter. Mixtures of linear alkanes show increasing deviations as the mixtures become more asymmetric with respect to size, as has been discussed previously [199]. Figure 2 shows that the deviations of the parachor model for a series of mixtures of components of varying chain lengths (pentane, heptane, decane, and dodecane). Note that the full citations for the reference codes used in the figures are given in Table 2. The pentane/hexadecane mixture has the largest size difference, and the largest deviation, reaching 2.5 mN·m^−1^, and this deviation can be reduced with the use of a fitted binary interaction parameter to 1 mN·m^−1^ indicating that even mixtures of linear alkanes that only have size differences can benefit from the use of a binary interaction parameter. The temperatures of the data covered 293 K to 598 K, the details for each data set are given in Table 2. Although we used a simple constant binary interaction parameter, Hugill and Van Welsenes [16] and Gasem *et al.* [200] pointed out that the binary interaction parameters are temperature dependent, and introducing temperature dependence in the interaction parameters could further reduce the deviations.

Mixtures of n-alkanes with dimethyl carbonate and hexadecane with a siloxane have larger deviations with the maximum absolute deviation of approximately 2 mN·m^−1^, and although the use of an interaction parameter can reduce the deviations, the parachor model does not perform quite as well for these systems (with a max AD of ~ 1 mN·m^−1^) as it does for the *n*-alkane/*n*-alkane systems that often have max AD of 0.7 mN·m^−1^ or less with an interaction parameter. There are three mixtures of methane with cryogens; methane/argon and methane/krypton were represented to within 3 % without an interaction parameter, but methane/nitrogen required a binary interaction parameter to achieve an AAPD of less than 4 %.

**Fig. 3:**
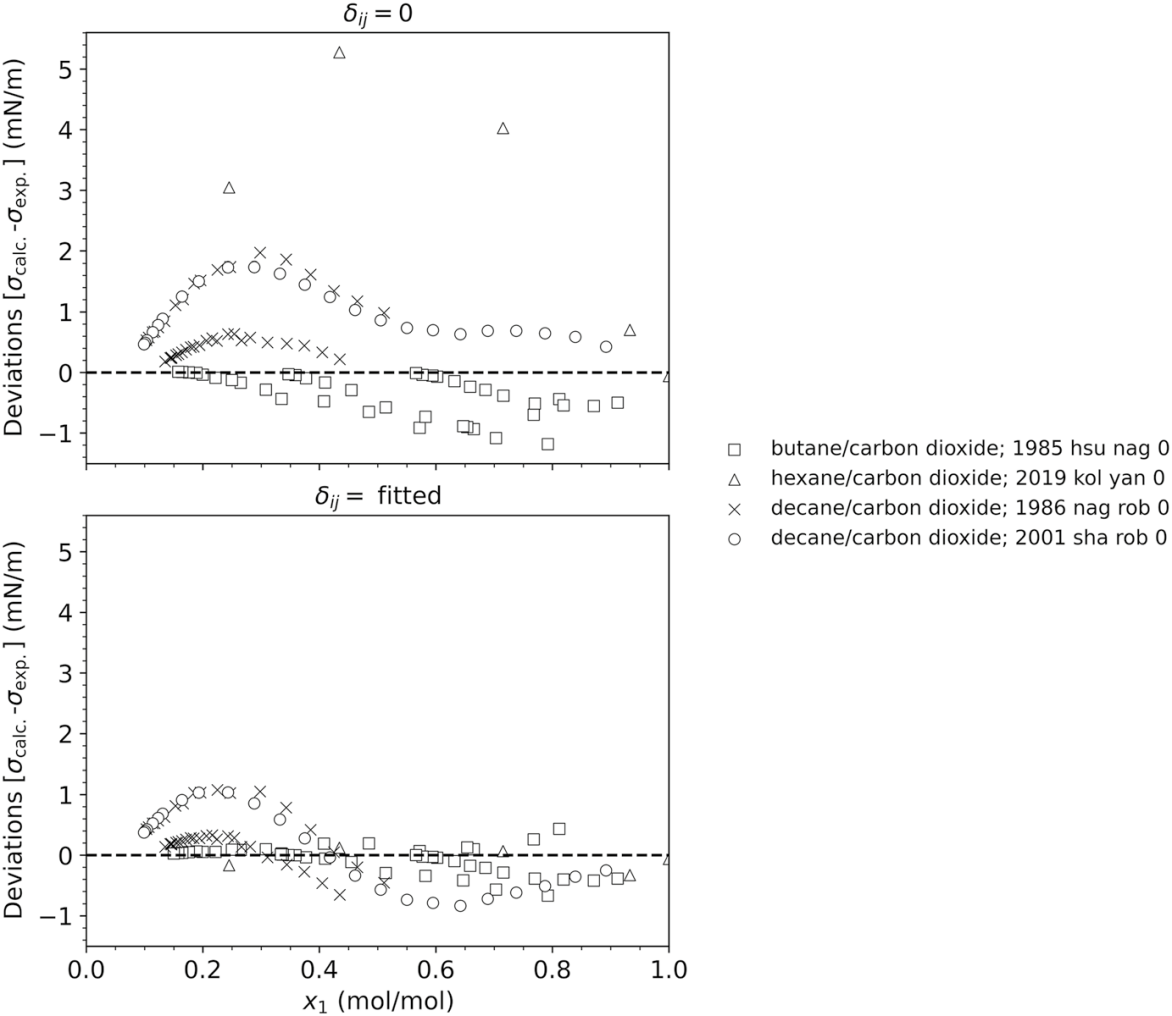
Deviations between the model and experimental data for mixtures of a series of *n*-alkanes with carbon dioxide

Finally, the parachor model without interaction parameters does not adequately capture the mixture composition behavior of *n*-alkanes with carbon dioxide, and an interaction parameter is needed. This is illustrated in Fig. 3. The temperatures of the data covered 303 K to 378 K, the details for each data set are given in Table 2. Similar to what is indicated in Fig. 2, Fig. 3 shows the largest deviations occur for systems with the largest size differences, with decane/CO_2_ showing larger deviations than hexane/CO_2_ and butane/CO_2_. For the hexane/CO_2_ mixture without interaction parameters, the AAPD is near 30 % but can be reduced to less than 2 % (0.3 mN·m^−1^) with a binary interaction parameter. Note that the percentage deviations for decane/CO_2_ and butane/CO_2_ are still large even with a binary interaction parameter, but this is because the data sets contain points approaching the critical region where the values of the surface tensions are small and the resulting percentage deviations are very large.

### Mixtures with Alcohols

**Fig. 4:**
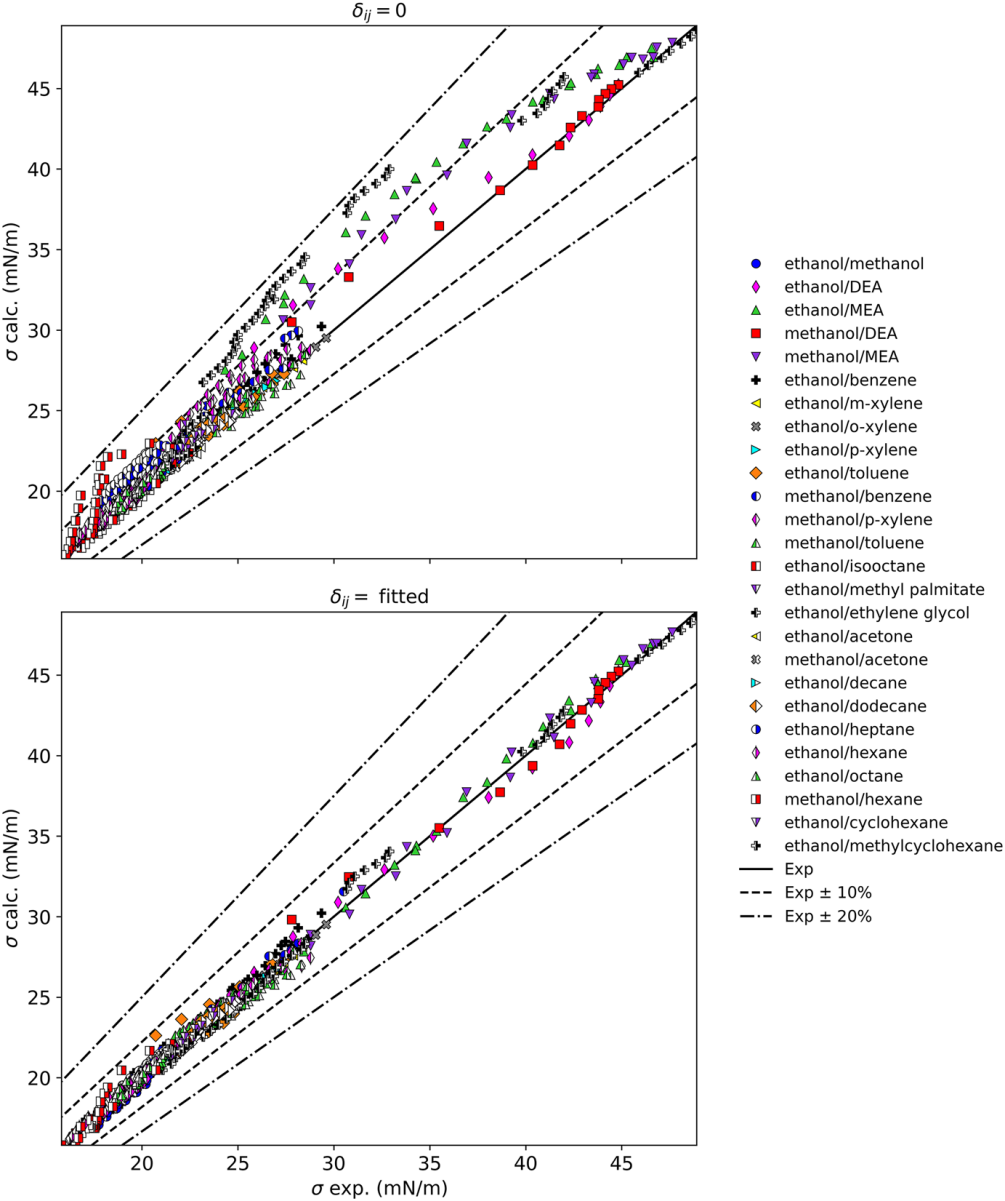
Summary of results for mixtures with alcohols

Table 4 summarizes the results for mixtures with alcohols, and Fig. 4 displays these results graphically. We include only mixtures with methanol and ethanol; larger alcohols are not presently available in REFPROP. This group of mixtures includes alcohols with a variety of fluid types [alcohols, *n*-alkanes, branched alkanes, amines, aromatics, glycols, ketones, naphthenes, and a fatty acid methyl ester (FAME)]. Mixtures with water are excluded and treated in Sect. 4.3. For the binary mixture of methanol and ethanol, the parachor method represents the surface tension to essentially within experimental uncertainty, and an interaction parameter is unnecessary. Similarly, mixtures of methanol and ethanol with acetone are represented very well without an interaction parameter. Mixtures of alcohols with aromatics and alcohols with linear alkanes have AAPD’s without binary interaction parameters ranging from roughly 1 % to 5 %, which can be reduced to less than 3 % with binary interaction parameters. In Fig. 4, for mixtures without binary interaction parameters, mixtures of alcohols with the amines MEA and DEA, and with ethylene glycol show large deviations. Of the components in mixtures with methanol and ethanol, pure MEA, DEA, and ethylene glycol have the largest values of surface tension (approximately 45 mN·m^−1^ at 313 K) compared to less than about 27 mN·m^−1^ for the other fluids in Table 4, and approximately 21 mN·m^−1^ for pure methanol and ethanol. Maximum deviations can be as large as 7 mN·m^−1^ for the mixtures with these three fluids and the parachor model is not recommended without a binary interaction parameter. With a binary interaction parameter, the maximum deviations can be reduced to 1–2 mN·m^−1^.

**Table 4:** Summary of results for alcohol mixtures

Mixture class	Fluids	Npts	*δ*_ij_ = 0			Fitted results			
			AAPD (%)	AAD (mN·m^−1^)	max AD (mN·m^−1^)	AAPD (%)	AAD (mN·m^−1^)	max AD (mN·m^−1^)	*δ*_ij_
Alcohol/alcohol	Ethanol/methanol	39	0.37	0.08	0.18	0.21	0.04	0.18	− 0.002
Alcohol/amine	Ethanol/DEA	13	4.78	1.47	3.67	1.83	0.64	1.44	0.046
Alcohol/amine	Ethanol/MEA	26	10.65	3.55	5.45	1.47	0.52	1.17	0.089
Alcohol/amine	Methanol/DEA	14	1.97	0.66	2.68	1.80	0.63	2.00	0.013
Alcohol/amine	Methanol/MEA	22	7.43	2.62	4.84	1.23	0.47	1.06	0.063
Alcohol/aromatic	Ethanol/benzene	76	2.71	0.64	1.59	1.53	0.36	1.16	0.020
Alcohol/aromatic	Ethanol/*m*-xylene	11	1.07	0.27	0.52	0.79	0.20	0.32	0.006
Alcohol/aromatic	Ethanol/*o*-xylene	11	0.83	0.21	0.45	0.56	0.15	0.21	0.005
Alcohol/aromatic	Ethanol/*p*-xylene	11	0.99	0.24	0.55	0.79	0.20	0.33	0.006
Alcohol/aromatic	Ethanol/toluene	15	2.60	0.60	2.25	2.95	0.69	1.90	0.015
Alcohol/aromatic	Methanol/benzene	19^a^	5.00	1.25	2.04	1.42	0.35	0.92	0.033
Alcohol/aromatic	Methanol/*p*-xylene	44	5.42	1.30	2.58	1.42	0.35	1.39	0.040
Alcohol/aromatic	Methanol/toluene	55	2.32	0.55	1.17	2.06	0.50	1.38	0.007
Alcohol/*br*-alkane	Ethanol/isooctane	44	0.86	0.16	0.45	0.66	0.13	0.32	− 0.005
Alcohol/FAME	Ethanol/methyl palmitate	1	8.08	1.80	1.80	0.00	0.00	0.00	0.089
Alcohol/glycol	Ethanol/ethylene glycol	56	11.44	3.26	7.09	1.67	0.47	1.40	0.100
Alcohol/ketone	Ethanol/acetone	5	1.08	0.25	0.56	0.82	0.19	0.36	− 0.008
Alcohol/ketone	Methanol/acetone	20	0.52	0.12	0.32	0.44	0.10	0.32	0.001
Alcohol/*n*-alkane	Ethanol/decane	32	0.88	0.19	0.53	0.76	0.16	0.40	0.004
Alcohol/*n*-alkane	Ethanol/dodecane	22	1.43	0.33	0.85	0.92	0.21	0.42	0.010
Alcohol/*n*-alkane	Ethanol/heptane	88	5.81	1.14	2.15	1.82	0.36	0.66	0.045
Alcohol/*n*-alkane	Ethanol/hexane	114	1.80	0.34	0.79	0.99	0.19	0.41	0.013
Alcohol/*n*-alkane	Ethanol/octane	48	1.14	0.23	0.77	0.78	0.16	0.61	0.008
Alcohol/*n*-alkane	Methanol/hexane	26	8.21	1.47	3.89	3.16	0.56	1.51	0.073
Alcohol/naphthene	Ethanol/cyclohexane	17	1.98	0.44	0.83	0.82	0.18	0.43	0.015
Alcohol/naphthene	Ethanol/methylcyclohexane	10	1.82	0.39	0.83	1.07	0.23	0.41	0.015

^a^One point omitted from statistics due to REFPROP calculation problems

### Mixtures with Water

**Fig. 5:**
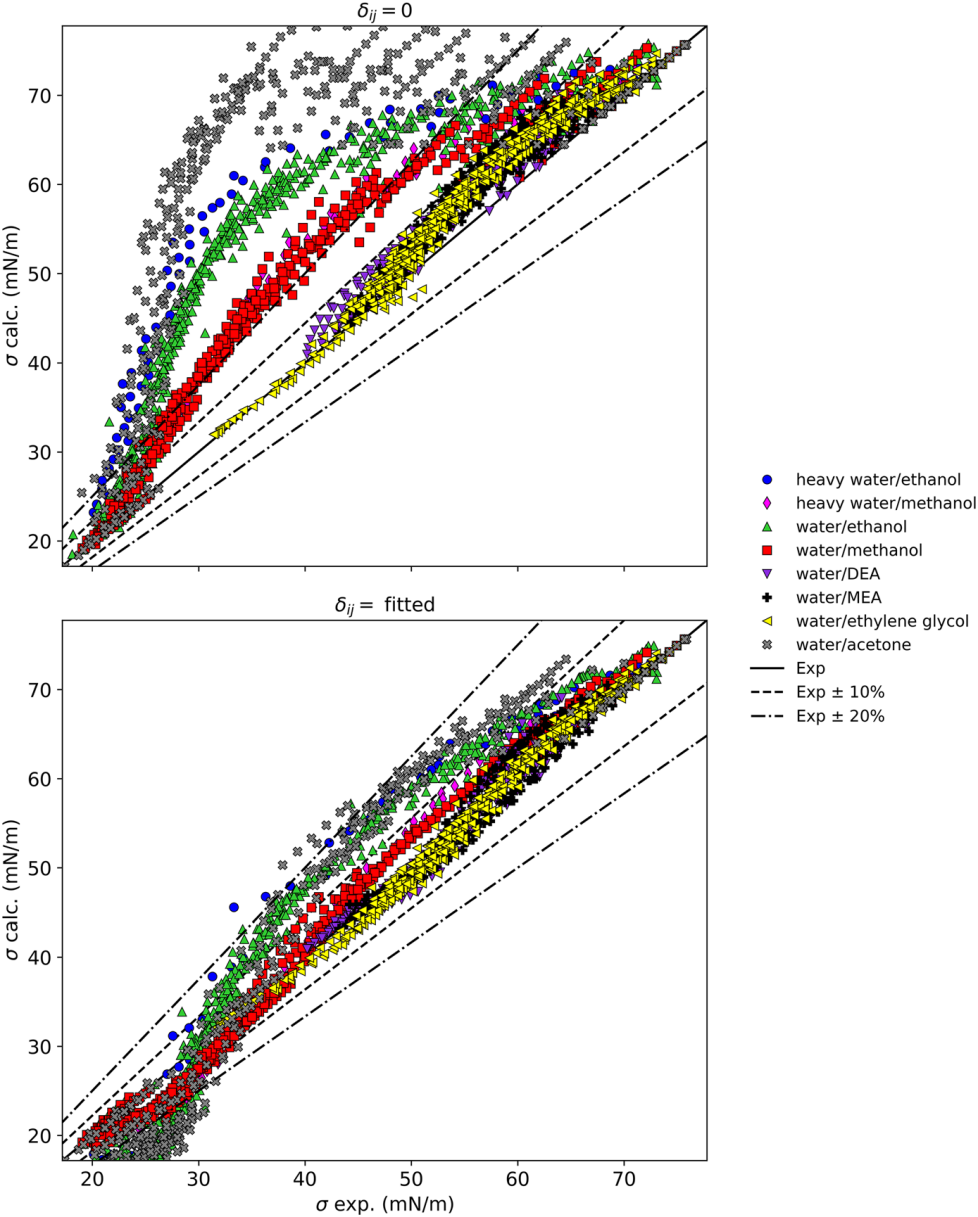
Summary of results for aqueous mixtures

**Fig. 6:**
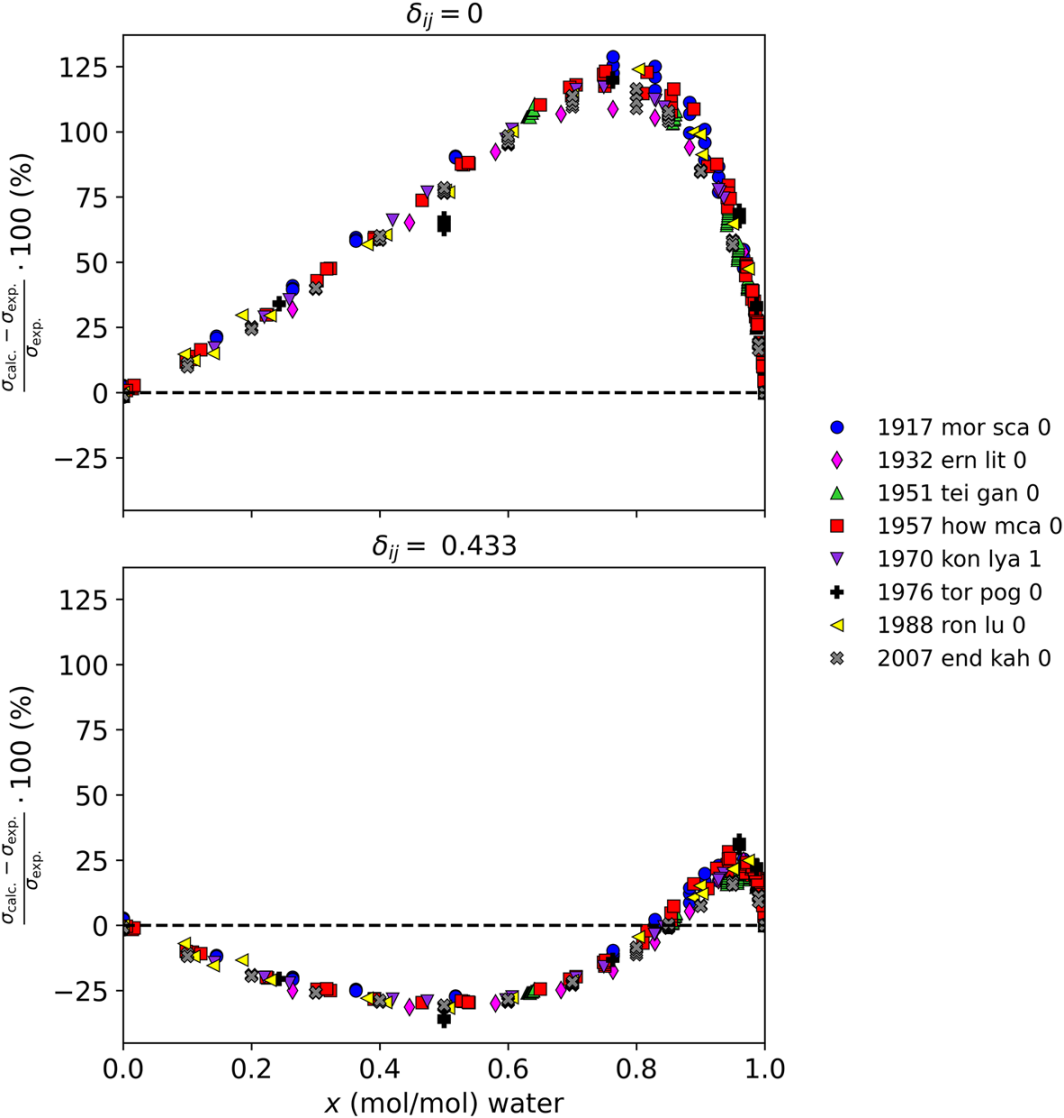
Relative deviations as a function of composition of water for acetone/water mixture

Table 5 summarizes the results for mixtures with water, and Fig. 5 displays these results graphically. The mixtures of water and heavy water with methanol and ethanol, and those with water and acetone show extremely large deviations, with a maximum AAD reaching 15–41 mN·m^−1^. The parachor model completely fails to represent the surface tension of these mixtures. The use of a single, constant binary interaction parameter somewhat reduces the magnitude of the deviations, but the model is still not very good with maximum deviations on the order of 5–13 mN·m^−1^. Without interaction parameters, all organic/aqueous mixtures investigated here show a common deviation pattern, where the deviations have an asymmetric shape with respect to composition, with a very rapid change as one nears the pure water end [8]. Water also has a very high surface tension (~ 70 mM·m^−1^ at 313 K) compared to other fluids. Figure 6, showing the percentage deviations of the acetone/water mixture as a function of composition, illustrates this pattern. The temperatures of the data covered 273 K to 343 K, the details for each data set are given in Table 2. One can see that although the use of an interaction parameter can somewhat reduce the size of the deviations, it cannot properly reproduce the composition behavior. A small amount of the organic can greatly change the surface tension, and the parachor model does not have the ability to model this composition behavior. It is possible that a more complex, composition and temperature dependent interaction parameter could capture this behavior, but it is beyond the scope of this work.

**Table 5:** Summary of results for aqueous mixtures

Mixture class	Fluids	Npts	*δ*_ij_ = 0			Fitted results			
			AAPD (%)	AAD (mN·m^−1^)	max AD (mN·m^−1^)	AAPD (%)	AAD (mN·m^−1^)	max AD (mN·m^−1^)	*δ*_ij_
Water/alcohol	Heavy water/ethanol	84	40.53	13.18	27.65	15.06	5.14	12.25	0.353
Water/alcohol	Heavy water/methanol	64	23.30	9.19	14.99	7.22	2.85	5.58	0.184
Water/alcohol	Water/ethanol	615	32.34	11.24	25.27	15.23	5.07	12.90	0.304
Water/alcohol	Water/methanol	401	18.62	7.14	16.73	5.48	2.04	5.42	0.164
Water/amine	Water/DEA	212	4.93	2.67	6.74	1.84	1.03	5.79	0.037
Water/amine	Water/MEA	273	5.41	3.05	7.10	2.30	1.32	4.13	0.041
Water/glycol	Water/ethylene glycol	427	4.91	2.77	7.18	2.03	1.11	4.40	0.041
Water/ketone	Water/acetone	403	55.44	18.90	40.51	14.90	5.33	12.96	0.433

### Mixtures with Aromatics

**Fig. 7:**
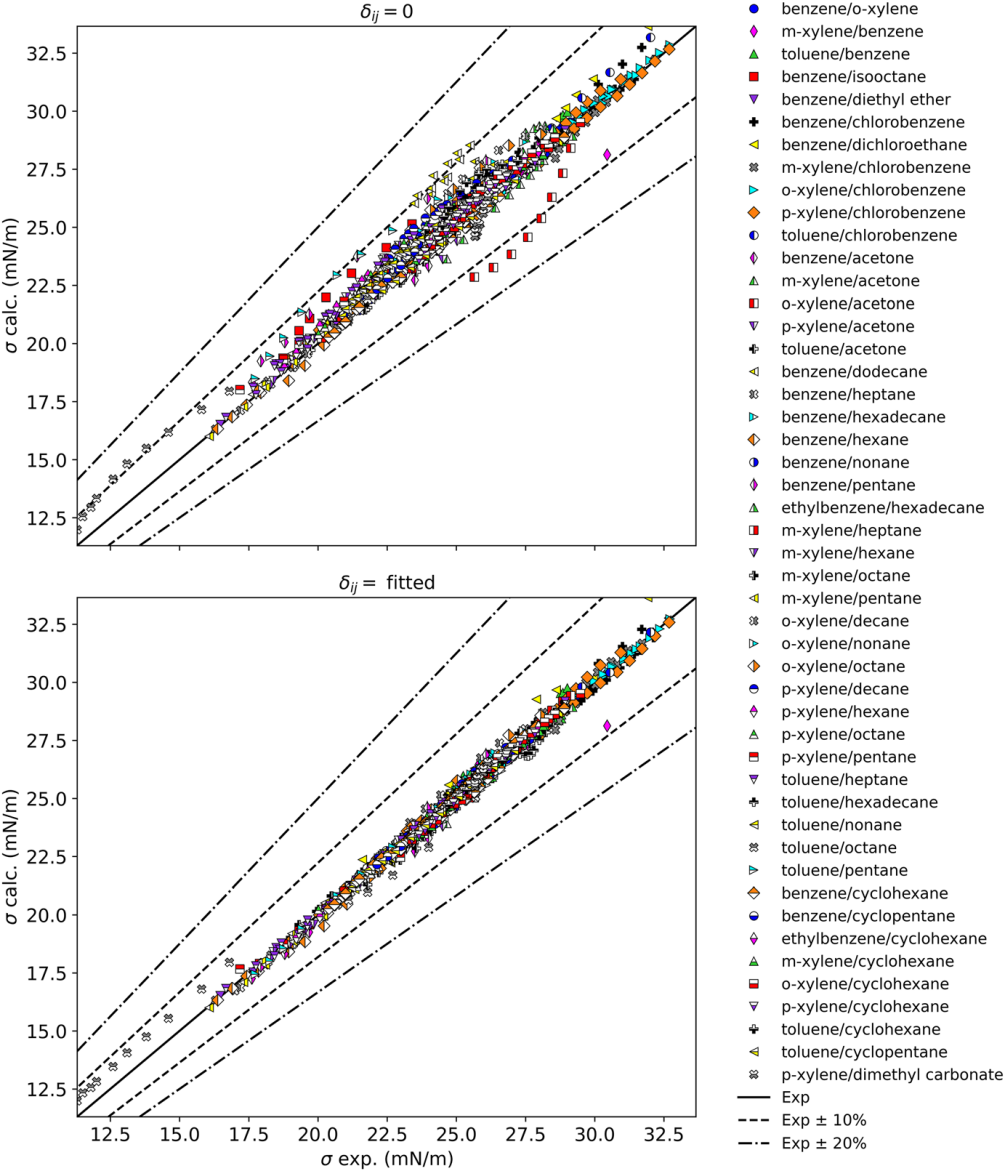
Summary of results for mixtures with aromatics

Table 6 summarizes the results for mixtures with aromatics, and Fig. 7 displays these results graphically. With a fitted binary interaction parameter all mixtures show an AAPD below 5 % except for the mixture toluene/octane that has an AAPD of 5 %. Without interaction parameters some of the points exceed 10 % deviation. However, these points occur at relatively high temperatures (380 K to 400 K) where the magnitude of the deviation is not excessively large (AAD of less than 0.82 mN·m^−1^) but the percentage deviations are larger due to the smaller value of the surface tension at higher temperatures. Other systems with deviations of approximately 10 % without interaction parameters are *o*-xylene/acetone, and some points in benzene/dodecane, toluene/pentane, and *m*-xylene/benzene. With interactions parameters the AADP in these systems, except *m*-xylene/benzene, can be reduced to 1 %. The *m*-xylene/benzene point with near 10 % deviation (for both fitted and non-fitted cases) is due to a pure fluid point for *m*-xylene (that we believe is flawed) from the 1929 data set of Hammick and Andrew [56]. In summary, when a fitted binary interaction parameter is used, the aromatic/alkane and aromatic/napthene mixtures have an AAPD of no greater than 1 %, however the deviations are a function of composition. With the use of a binary interaction parameter these mixtures can generally be represented to within 3 % over the entire composition range.

**Table 6:** Summary of results for aromatic mixtures

Mixture class	Fluids	Npts	*δ*_ij_ = 0			Fitted results			
			AAPD (%)	AAD (mN·m^−1^)	max AD (mN·m^−1^)	AAPD (%)	AAD (mN·m^−1^)	max AD (mN·m^−1^)	*δ*_ij_
Aromatic/aromatic	Benzene/*o*-xylene	8	1.33	0.38	0.52	0.32	0.09	0.15	0.008
Aromatic/aromatic	*m*-Xylene/benzene	5	2.80	0.82	2.33	1.80	0.54	2.33	0.010
Aromatic/aromatic	Toluene/benzene	27	1.05	0.28	0.99	1.30	0.34	0.76	0.005
Aromatic/*br*-alkane	Benzene/isooctane	9	5.39	1.13	1.81	0.48	0.10	0.35	0.041
Aromatic/ether	Benzene/diethyl ether	4	2.48	0.52	0.90	0.42	0.09	0.15	0.020
Aromatic/halocb	Benzene/chlorobenzene	23	1.22	0.36	1.04	1.06	0.31	0.73	0.008
Aromatic/halocb	Benzene/dichloroethane	13	3.85	1.05	1.74	2.09	0.57	1.74	0.021
Aromatic/halocb	*m*-Xylene/chlorobenzene	18	0.62	0.18	0.61	0.62	0.18	0.63	0.001
Aromatic/halocb	*o*-Xylene/chlorobenzene	18	0.73	0.22	0.42	0.23	0.07	0.15	0.005
Aromatic/halocb	*p*-Xylene/chlorobenzene	18	0.80	0.24	0.67	0.87	0.26	0.53	0.004
Aromatic/halocb	Toluene/chlorobenzene	6	3.35	0.97	1.16	0.59	0.17	0.28	0.021
Aromatic/ketone	Benzene/acetone	33	0.94	0.24	0.77	0.85	0.21	0.77	0.003
Aromatic/ketone	*m*-Xylene/acetone	9	2.14	0.56	1.02	0.76	0.20	0.74	− 0.013
Aromatic/ketone	*o*-Xylene/acetone	8	8.78	2.39	3.16	1.06	0.29	0.62	− 0.062
Aromatic/ketone	*p*-Xylene/acetone	9	1.36	0.35	0.91	0.85	0.22	0.75	− 0.008
Aromatic/ketone	Toluene/acetone	110	1.10	0.27	0.83	0.84	0.20	0.71	0.007
Aromatic/*n*-alkane	Benzene/dodecane	23	6.71	1.65	3.15	0.95	0.24	0.64	0.060
Aromatic/*n*-alkane	Benzene/heptane	47	1.57	0.35	1.16	1.02	0.22	0.87	0.012
Aromatic/*n*-alkane	Benzene/hexadecane	44	1.08	0.28	0.69	1.01	0.26	0.55	0.005
Aromatic/*n*-alkane	Benzene/hexane	31	1.86	0.41	1.08	1.20	0.26	0.73	0.015
Aromatic/*n*-alkane	Benzene/nonane	44	1.52	0.35	0.77	0.57	0.14	0.34	0.012
Aromatic/*n*-alkane	Benzene/pentane	7	7.62	1.61	2.33	2.04	0.44	0.81	0.051
Aromatic/*n*-alkane	Ethylbenzene/hexadecane	9	3.63	1.01	1.58	0.45	0.12	0.27	0.026
Aromatic/*n*-alkane	*m*-Xylene/heptane	11	2.31	0.54	0.89	0.13	0.03	0.11	0.019
Aromatic/*n*-alkane	*m*-Xylene/hexane	29	3.00	0.68	1.30	0.87	0.19	0.35	0.024
Aromatic/*n*-alkane	*m*-Xylene/octane	11	2.33	0.57	0.98	0.16	0.04	0.07	0.020
Aromatic/*n*-alkane	*m*-Xylene/pentane	20	1.46	0.32	0.84	0.91	0.20	0.36	0.012
Aromatic/*n*-alkane	*o*-Xylene/decane	11	5.26	1.33	1.86	0.81	0.21	0.58	0.039
Aromatic/*n*-alkane	*o*-Xylene/nonane	11	4.59	1.14	1.68	0.92	0.23	0.52	0.034
Aromatic/*n*-alkane	*o*-Xylene/octane	11	4.83	1.17	1.73	1.09	0.27	0.84	0.037
Aromatic/*n*-alkane	*p*-Xylene/decane	22	4.68	1.12	1.51	0.67	0.17	0.49	0.031
Aromatic/*n*-alkane	*p*-Xylene/hexane	16	3.42	0.76	1.13	0.51	0.11	0.21	0.024
Aromatic/*n*-alkane	*p*-Xylene/octane	12	3.90	0.88	1.19	0.83	0.19	0.53	0.027
Aromatic/*n*-alkane	*p*-Xylene/pentane	7	3.30	0.68	0.89	0.97	0.20	0.51	0.019
Aromatic/*n*-alkane	Toluene/heptane	34	2.88	0.62	1.28	0.55	0.12	0.32	0.024
Aromatic/*n*-alkane	Toluene/hexadecane	52	1.80	0.47	1.06	0.84	0.22	0.82	0.013
Aromatic/*n*-alkane	Toluene/nonane	44	2.37	0.55	0.99	0.34	0.08	0.29	0.019
Aromatic/*n*-alkane	Toluene/octane	17	6.06	0.82	1.73	5.03	0.77	1.15	0.027
Aromatic/*n*-alkane	Toluene/pentane	8	8.31	1.72	2.24	0.91	0.18	0.30	0.050
Aromatic/naphthene	Benzene/cyclohexane	96	0.53	0.13	0.37	0.50	0.13	0.41	0.001
Aromatic/naphthene	Benzene/cyclopentane	9	1.63	0.40	0.54	0.22	0.06	0.14	0.011
Aromatic/naphthene	Ethylbenzene/cyclohexane	28	1.56	0.40	0.82	0.50	0.13	0.24	0.013
Aromatic/naphthene	*m*-Xylene/cyclohexane	28	1.38	0.35	0.70	0.36	0.09	0.31	0.011
Aromatic/naphthene	*o*-Xylene/cyclohexane	28	1.31	0.34	0.76	0.45	0.11	0.26	0.010
Aromatic/naphthene	*p*-Xylene/cyclohexane	28	1.13	0.29	0.56	0.31	0.08	0.23	0.009
Aromatic/naphthene	Toluene/cyclohexane	11	4.40	1.12	1.55	0.11	0.03	0.07	0.031
Aromatic/naphthene	Toluene/cyclopentane	10	0.20	0.05	0.07	0.07	0.02	0.03	− 0.001
Aromatic/other	*p*-Xylene/dimethyl carbonate	10	2.39	0.62	1.09	0.72	0.19	0.42	− 0.019

### Mixtures with Halocarbons

**Fig. 8:**
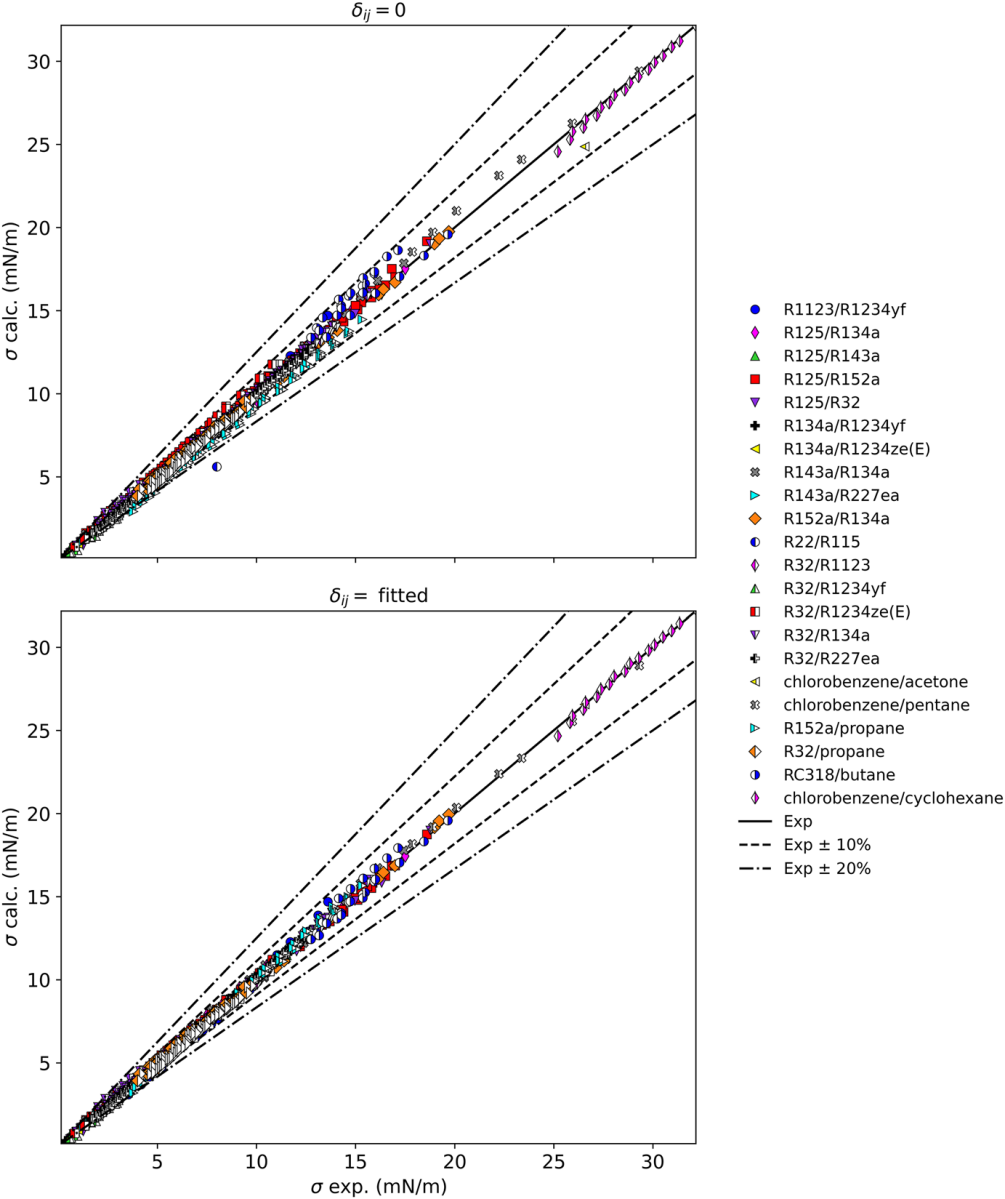
Summary of results for halocarbon mixtures

Table 7 summarizes the results for mixtures with halocarbons, and Fig. 8 displays these results graphically. Included are mixtures containing some of the new low-GWP fluids such as R1234yf and R1234ze(E) in addition to HFC’s such as R32, R134a, R143a, R152a, and R125, and mixtures of polar halocarbons with nonpolar alkanes such as propane and butane. Without using an interaction parameter, almost all results are within 10 %, the AAPD’s are generally less than 5 %. Exceptions are visible in Fig. 8; the single point for R22/R115 has very large deviations; it is unclear why this mixture should deviate from the others. The mixture of R152a/propane also has deviations slightly greater than 10 % without an interaction parameter. It is unclear why R152a/propane should show this magnitude of deviation (AAPD 10.6 %), as a similar polar/nonpolar mixture of R32/propane displays smaller deviations (AAPD 2.6 %) without the use of an interaction parameter. The mixtures of halocarbons with other halocarbons without an interaction parameter have AAD of about 0.3 mN·m^−1^, while the mixtures of polar halocarbons with nonpolar alkanes have a higher AAD of up to 0.9 mN·m^−1^. The use of an interaction parameter improves the results, providing an AAD less than 0.35 mN·m^−1^ for both types of mixtures.

**Table 7:** Summary of results for halocarbon mixtures

Mixture class	Fluids	Npts	*δ*_ij_ = 0			Fitted results			
			AAPD (%)	AAD (mN·m^−1^)	max AD (mN·m^−1^)	AAPD (%)	AAD (mN·m^−1^)	max AD (mN·m^−1^)	*δ*_ij_
Halocb/halocb	R1123/R1234yf	39	4.37	0.32	1.11	4.43	0.31	1.09	0.002
Halocb/halocb	R125/R134a	21	1.13	0.07	0.25	0.99	0.06	0.18	0.004
Halocb/halocb	R125/R143a	38	2.07	0.09	0.28	1.65	0.05	0.16	0.008
Halocb/halocb	R125/R152a	75	3.24	0.25	0.68	1.66	0.11	0.31	0.020
Halocb/halocb	R125/R32	262	1.87	0.07	0.38	1.81	0.05	0.50	0.004
Halocb/halocb	R134a/R1234yf	23	8.05	0.19	0.37	4.88	0.09	0.22	− 0.025
Halocb/halocb	R134a/R1234ze(E)	9	2.71	0.10	0.25	2.43	0.04	0.06	0.012
Halocb/halocb	R143a/R134a	126	1.73	0.11	0.69	1.96	0.12	0.61	0.002
Halocb/halocb	R143a/R227ea	241	3.57	0.17	0.31	1.89	0.08	0.21	− 0.013
Halocb/halocb	R152a/R134a	21	1.50	0.15	0.38	1.06	0.12	0.33	− 0.006
Halocb/halocb	R22/R115	1	29.91	2.39	2.39	0.00	0.00	0.00	− 0.196
Halocb/halocb	R32/R1123	37	4.24	0.27	0.56	2.29	0.14	0.41	− 0.021
Halocb/halocb	R32/R1234yf	60	7.65	0.23	0.47	6.24	0.18	0.43	− 0.017
Halocb/halocb	R32/R1234ze(E)	52	4.14	0.28	0.97	3.07	0.15	0.41	0.026
Halocb/halocb	R32/R134a	317	1.08	0.07	0.47	1.11	0.07	0.47	0.001
Halocb/halocb	R32/R227ea	412	3.90	0.19	0.43	2.58	0.12	0.36	− 0.012
Halocb/ketone	Chlorobenzene/acetone	1	6.36	1.69	1.69	0.00	0.00	0.00	− 0.036
Halocb/*n*-alkane	Chlorobenzene/pentane	7	3.04	0.63	0.91	1.20	0.27	0.42	0.017
Halocb/*n*-alkane	R152a/propane	51	10.61	0.87	1.09	3.54	0.25	0.60	− 0.050
Halocb/*n*-alkane	R32/propane	99	2.60	0.16	0.51	2.53	0.15	0.45	− 0.010
Halocb/*n*-alkane	RC318/butane	24	5.89	0.88	1.68	2.27	0.34	0.79	0.044
Halocb/naphthene	Chlorobenzene/cyclohexane	18	0.89	0.24	0.64	0.49	0.13	0.54	− 0.005

### Mixtures with Miscellaneous Compounds

**Fig. 9:**
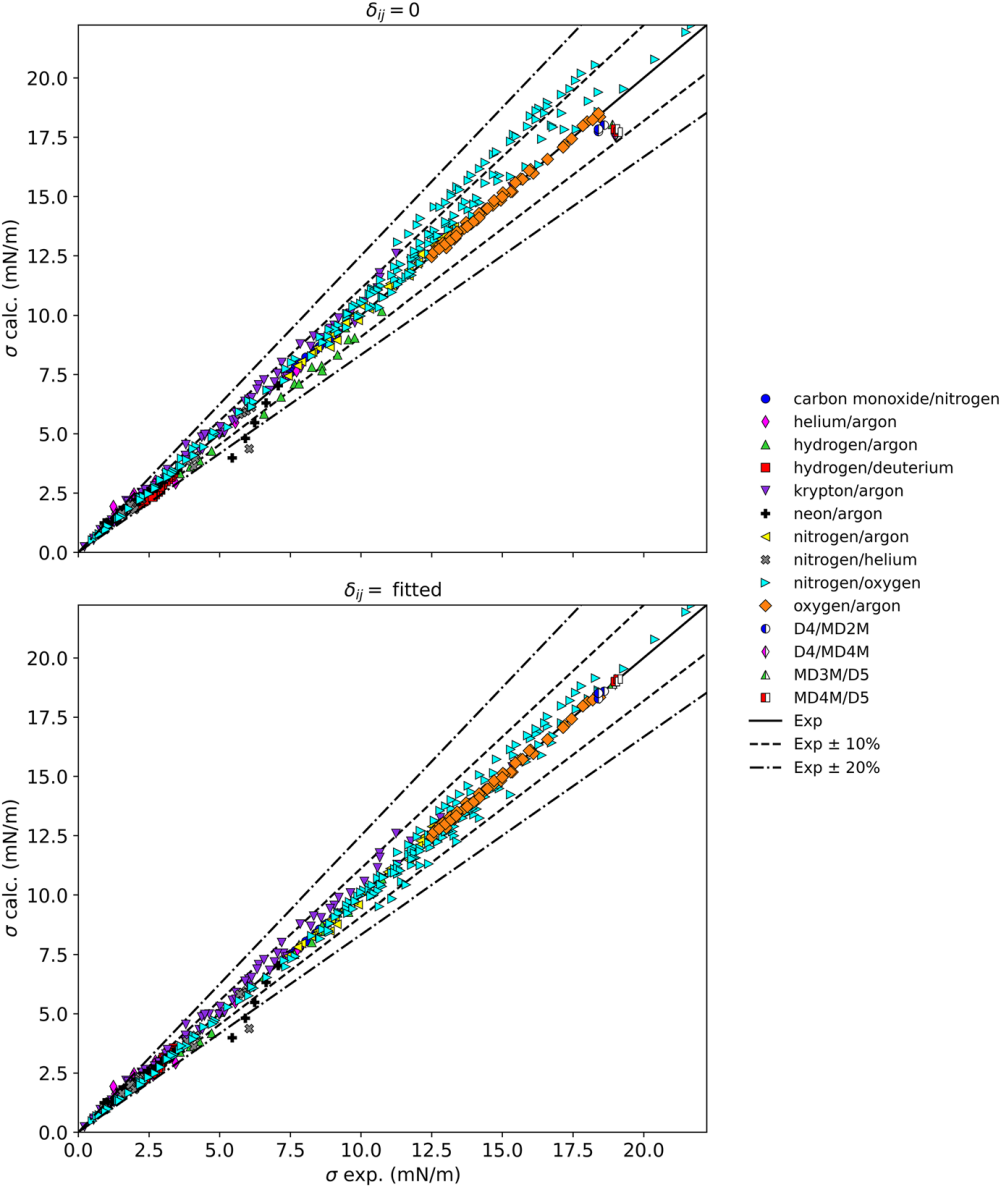
Summary of results for mixtures with miscellaneous compounds

Table 8 summarizes the results for mixtures with miscellaneous compounds, and Fig. 9 displays these results graphically. The mixtures are either of cryogens with other cryogens, or siloxanes with siloxanes. All mixtures without interaction parameters except helium/argon show an AAPD of less than 10 %. As shown in Table 2, the helium/argon mixture data were obtained only for extremely dilute solutions of helium less than about a helium mole fraction of 0.01. Without more data over a larger composition range, it is difficult to assess the performance of the parachor model for the helium/argon system. In addition, there were convergence failures in REFPROP for the systems helium/argon, krypton/argon, and nitrogen/helium; points without convergence were not included in the statistics and binary interaction parameters were not determined for these systems. There also was an extremely limited composition range for neon/argon, hydrogen/argon, and nitrogen/helium so we cannot fully assess these systems either. The data for siloxane mixtures are very limited in the number of points, so it also is premature to assess these systems. For the cryogen/cryogen mixtures where there are a wide range of data, the parachor model appears to represent the data to within 10 % without interaction parameters, with AAPD’s of less than 5 %.

**Table 8:** Summary of results for miscellaneous mixtures

Mixture class	Fluids	Npts	*δ*_ij_ = 0			Fitted results			
			AAPD (%)	AAD (mN·m^−1^)	max AD (mN·m^−1^)	AAPD (%)	AAD (mN·m^−1^)	max AD (mN·m^−1^)	*δ*_ij_
Cryogen/cryogen	Carbon monoxide/nitrogen	10	1.28	0.10	0.18	0.21	0.02	0.04	0.012
Cryogen/cryogen	Helium/argon	33^a^	13.08	0.25	0.69	13.08	0.25	0.69	0.000
Cryogen/cryogen	Hydrogen/argon	21	6.42	0.44	0.96	2.92	0.15	0.54	− 0.678
Cryogen/cryogen	Hydrogen/deuterium	67	4.98	0.13	0.30	2.65	0.07	0.19	− 0.033
Cryogen/cryogen	Krypton/argon	100^b^	4.92	0.24	1.35	4.92	0.24	1.35	0.000
Cryogen/cryogen	Neon/argon	27	7.82	0.22	1.47	8.13	0.23	1.46	0.048
Cryogen/cryogen	Nitrogen/argon	40	1.96	0.21	0.50	1.15	0.13	0.41	0.012
Cryogen/cryogen	Nitrogen/helium	38^c^	4.94	0.16	1.67	4.94	0.16	1.67	0.000
Cryogen/cryogen	Nitrogen/oxygen	183	5.41	0.68	2.41	3.31	0.33	1.26	0.055
Cryogen/cryogen	Oxygen/argon	64	0.48	0.07	0.19	0.48	0.07	0.19	0.001
Siloxane/siloxane	D4/MD2M	3	3.26	0.60	0.66	0.43	0.08	0.12	− 0.019
Siloxane/siloxane	D4/MD4M	2	7.35	1.40	1.43	0.28	0.05	0.06	− 0.047
Siloxane/siloxane	MD3M/D5	1	4.56	0.86	0.86	0.00	0.00	0.00	− 0.024
Siloxane/siloxane	MD4M/D5	3	6.55	1.25	1.39	0.02	0.00	0.01	− 0.039

^a^14 points omitted from statistics due to REFPROP calculation problems

^b^3 points omitted from statistics due to REFPROP calculation problems

^c^15 points omitted from statistics due to REFPROP calculation problems

## Conclusions

We compiled a database for the surface tension of binary mixtures by extracting data from the NIST TDE database [34]. It contains a wide variety of fluids, covering the chemical classes water, alcohols, amines, ketones, linear and branched alkanes, naphthenes, aromatics, refrigerants, and cryogens. The data set includes 65 pure fluids and 154 binary pairs with a total of 8205 points. We used this database to test the performance of a parachor model for mixtures, in both a predictive mode (no mixture data used) and with a single, constant binary interaction parameter found by fitting the mixture data. The parachor model is not new and variants of it have been used for many years, but a comprehensive summary of its performance on a wide variety of mixtures has not been available until now. The data are available in the supporting information to enable model comparisons for future research on binary mixtures with new models. In general, the parachor model in a predictive mode without fitted interaction parameters can predict the surface tension of binary mixtures of non-polar fluids such as linear and branched alkanes, linear and branched alkanes with naphthenes, aromatics with aromatics, aromatics with naphthenes, and mixtures of linear alkanes of similar sizes with an AAPD of about 3 % or less. For mixtures of linear alkanes of differing sizes, as the size difference increases it is necessary to use a fitted binary interaction parameter to reduce deviations. Similarly, in a predictive mode the model has large deviations for mixtures of *n*-alkanes with CO_2_, and an interaction parameter should be used. Mixtures of methanol and ethanol did not require an interaction parameter. Polar mixtures of halocarbons with other halocarbons and also polar/nonpolar mixtures of alkanes with halocarbons could be modeled with an AAD of less than 0.35 mN·m^−1^ with the use of a binary interaction parameter for each pair of fluids. Future work on developing a predictive scheme for binary interaction parameters for classes of mixtures would make the parachor model more useful. Finally, the parachor model even with a fitted binary interaction parameter is not suitable for mixtures of water with organic compounds.

## Supplementary Information

Below is the link to the electronic supplementary material.

Supplementary file1 (PDF 573 KB)

Supplementary file2 (TXT 909 KB)

Supplementary file3 (PDF 2214 KB)

## Footnotes

1 Certain equipment, instruments, software, or materials, commercial or non-commercial, are identified in this paper to specify the experimental procedure adequately. Such identification is not intended to imply recommendation or endorsement of any product or service by NIST, nor is it intended to imply that the materials or equipment identified are necessarily the best available for the purpose.
