# Supplementary material for: Assessment of a Parachor Model for the Surface Tension of Binary Mixtures
Source: Int J Thermophys. 2023 Jun 7;44(7):110. doi: 10.1007/s10765-023-03216-z (PMC10247860; doi:10.1007/s10765-023-03216-z)
Supplement: Supplementary file 1 — Supplementary file1 (PDF 573 KB) [file 10765_2023_3216_MOESM1_ESM.pdf]

Appendix A: Changes and additions to REFPROP v10.0

The following changes were made:

- We used the REFPROP v10.0 program in our analysis but made several changes and additions to v10.0 that are described below.
- The value of the exponent *m* in Eq. 2 was changed from 3.86 to 3.87 in accordance with recent theory as described in the manuscript.
- In the source code, the maximum upper temperature limit on calculations in the surface tension subroutine STN was increased to the maximum of the value of Tc or Tcritf.
- In the subroutine UTILITY.FOR lines were added to enable accessing the surface tension binary interaction parameter "fstkij" from the DLL.
- Family names for DEA, MEA, EGLYCOL, ACETONE, HEAVY WATER, and WATER were changed in the fluid files to be consistent with Table 1. (Previously they were "other")
- The fluid files for DEA, EGLYCOL, and C16 had the following minor changes made:
    - DEA.FLD minimum temperature lowered from 301.1 K to 297 K, maximum density changed from 10.4 to 10.44 mol/l
    - C16.FLD maximum density increased from 3.423 mol/l to 3.5 mol/l
- Changes were made to the HMX.BNC file that contains interaction parameters for phase equilibrium properties.
    - Binary interaction parameters for mixtures R134a/R1234yf, R134a/R1234ze(E), R1234yf/R1234ze(E), R1234ze(E)/R227ea, R125/R1234yf, R1234yf/R152a were used as published in Bell[1]
    - We implemented the model of Beckmüller et al. [2] for H_2 + [CO, CO_2, N_2, CH_4].
    - We implemented the model of Neumann for DEA/water[3].
    - MEA/water, MEA/ethanol, DEA/methanol, MEA/ethanol, DEA/ethanol, toluene/hexadecane, dodecane/ethanol, hexadecane/benzene, pentane/hexadecane, ethanol/ethylene glycol were fit using TDE, internal version (Aug. 2022), and the resulting parameters are given in Table A1. Insufficient data were found for DEA/ethanol so parameters for DEA/methanol were used. The model used for XR0 is discussed in Bell and Lemmon[4]. The model for KW0 is discussed in [5].
    - We implemented parameters from Bell and Lemmon[4] for methanol/*p*-xylene, butane/RC318, ethanol/*m*-xylene, acetone/*m*-xylene, toluene/pentane, hexane/methanol.
    - We implemented the mixture model of Tkaczuk et al.[6] for binary mixtures of He-4, Neon, and Argon.

Table A1: Binary interaction parameters obtained from TDE.

Fluids	Model	β_T	γ_T	β_V	γ_V	F_{ij}
MEA/water	XR0	1.0036	1.01607	1.00171	1.0781	
MEA/methanol	XR0	1	1.15737	1	1.02181	
MEA/ethanol	XR0	1	1.01194	1	0.984648	
DEA/methanol	XR0	1.00901	1.07937	0.977159	1.04905	
DEA/ethanol	XR0	1.00901	1.07937	0.977159	1.04905	
pentane/hexadecane	XR0	0.985071	1.13351	1.01315	1.05461	
toluene/hexadecane	KW0	0.990966	1.04277	1.00669	1.07638	0.73764
dodecane/ethanol	KW0	0.978255	0.911439	0.973929	1.18355	3.46457
hexadecane/benzene	KW0	1.00201	1.05679	0.989713	1.09668	0.400853
ethanol/ethylene glycol	KW0	1.00415	1.01236	1.00393	0.982048	-0.407454

Appendix B: Table of results for all data sets listed in Table 2 of the main manuscript.

Table B1: Summary of Results

Ref.	fluid 1	fluid 2	δ_{ij} = 0 results			Fitted results			
			AADP (%)	AAD (mN/m)	max AD (mN/m)	AAD P (%)	AAD (mN/m)	max AD (mN/m)	δ_{ij}
[7]	ethylbenzene	cyclohexane	1.56	0.40	0.82	0.50	0.13	0.24	0.013
[8]	ethylbenzene	hexadecane	3.63	1.01	1.58	0.45	0.12	0.27	0.026
[9]	*p*-xylene	chlorobenzene	0.80	0.24	0.67	0.87	0.26	0.53	0.004
[10]	*p*-xylene	pentane	3.30	0.68	0.89	0.97	0.20	0.51	0.019
[11]	*p*-xylene	hexane	3.42	0.76	1.13	0.51	0.11	0.21	0.024
[7]	*p*-xylene	cyclohexane	1.13	0.29	0.56	0.31	0.08	0.23	0.009
[12]	*p*-xylene	octane	3.90	0.88	1.19	0.83	0.19	0.53	0.027
[13]	*p*-xylene	decane	4.29	1.06	1.31	0.67	0.17	0.41	0.031
[12]	*p*-xylene	decane	5.06	1.19	1.51	0.67	0.16	0.49	0.031
[14]	*p*-xylene	ethanol	0.99	0.24	0.55	0.79	0.20	0.33	0.006
[15]	*p*-xylene	methanol	5.42	1.30	2.58	1.42	0.35	1.39	0.040
[16]	*p*-xylene	acetone	1.36	0.35	0.91	0.85	0.22	0.75	-0.008
[12]	*p*-xylene	dimethyl-carbonate	2.39	0.62	1.09	0.72	0.19	0.42	-0.019
[17]	butane	RC318	5.89	0.88	1.68	2.27	0.34	0.79	0.044
[18]	butane	carbon dioxide	29.0[a]	0.38	1.18	21.0	0.17	0.67	-0.178
[19]	butane	methane	18.2	0.15	0.15	0.00	0.00	0.00	-0.089
[20]	dichloroethane	benzene	3.85	1.05	1.74	2.09	0.57	1.74	0.021
[9]	*m*-xylene	chlorobenzene	0.62	0.18	0.61	0.62	0.18	0.63	0.001
[10]	*m*-xylene	pentane	2.57	0.56	0.84	0.90	0.20	0.36	0.012
[21]	*m*-xylene	pentane	0.56	0.12	0.25	0.91	0.19	0.27	0.012
[22]	*m*-xylene	hexane	3.50	0.79	1.30	1.00	0.22	0.35	0.024
[21]	*m*-xylene	hexane	2.19	0.50	0.93	0.66	0.14	0.29	0.024
[7]	*m*-xylene	cyclohexane	1.38	0.35	0.70	0.36	0.09	0.31	0.011
[21]	*m*-xylene	octane	2.33	0.57	0.98	0.16	0.04	0.07	0.020
[21]	*m*-xylene	heptane	2.31	0.54	0.89	0.13	0.03	0.11	0.019
[14]	*m*-xylene	ethanol	1.07	0.27	0.52	0.79	0.20	0.32	0.006
[16]	*m*-xylene	acetone	2.14	0.56	1.02	0.76	0.20	0.74	-0.013
[23]	*m*-xylene	benzene	2.80	0.82	2.33	1.80	0.54	2.33	0.010
[24]	toluene	chlorobenzene	3.35	0.97	1.16	0.59	0.17	0.28	0.021
[10]	toluene	pentane	8.31	1.72	2.24	0.91	0.18	0.30	0.050
[25]	toluene	cyclohexane	4.40	1.12	1.55	0.11	0.03	0.07	0.031
[26]	toluene	octane	6.06	0.82	1.73	5.03	0.77	1.15	0.027
[27]	toluene	nonane	2.37	0.55	0.99	0.34	0.08	0.29	0.019
[28]	toluene	heptane	2.88	0.62	1.28	0.55	0.12	0.32	0.024
[25]	toluene	cyclopentane	0.20	0.05	0.07	0.07	0.02	0.03	-0.001
[8]	toluene	hexadecane	0.43	0.12	0.26	1.89	0.52	0.82	0.013
[27]	toluene	hexadecane	2.05	0.53	1.06	0.65	0.17	0.40	0.013
[29]	toluene	ethanol	0.42	0.10	0.16	2.04	0.51	0.86	0.015
[30]	toluene	ethanol	6.97	1.58	2.25	4.78	1.07	1.90	0.015
[31]	toluene	methanol	2.65	0.63	1.05	2.41	0.58	0.86	0.007
[15]	toluene	methanol	2.24	0.54	1.17	1.97	0.48	1.38	0.007
[32]	toluene	acetone	0.75	0.18	0.49	0.75	0.18	0.71	0.007
[33]	toluene	acetone	1.45	0.35	0.83	0.93	0.22	0.53	0.007

Ref.	fluid 1	fluid 2	δ_{ij} = 0 results			Fitted results			
			AADP (%)	AAD (mN/m)	max AD (mN/m)	AAD P (%)	AAD (mN/m)	max AD (mN/m)	δ_{ij}
[24]	toluene	benzene	3.45	0.93	0.99	2.63	0.71	0.76	0.005
[34]	toluene	benzene	0.36	0.09	0.22	0.92	0.24	0.39	0.005
[10]	chlorobenzene	pentane	3.04	0.63	0.91	1.20	0.27	0.42	0.017
[9]	chlorobenzene	cyclohexane	0.89	0.24	0.64	0.49	0.13	0.54	-0.005
[24]	chlorobenzene	acetone	6.36	1.69	1.69	0.00	0.00	0.00	-0.036
[24]	chlorobenzene	benzene	3.40	0.98	1.04	2.03	0.59	0.73	0.008
[9]	chlorobenzene	benzene	0.45	0.13	0.23	0.72	0.22	0.33	0.008
[9]	chlorobenzene	*o*-xylene	0.73	0.22	0.42	0.23	0.07	0.15	0.005
[10]	pentane	cyclohexane	2.02	0.42	0.62	0.45	0.09	0.16	0.013
[35]	pentane	heptane	14.0[b]	0.04	0.27	14.0[b]	0.04	0.27	0.000
[36]	pentane	heptane	1.33	0.20	0.67	1.33	0.20	0.67	0.000
[37]	pentane	hexadecane	8.07	1.58	2.45	1.67	0.31	1.05	0.045
[10]	pentane	benzene	7.62	1.61	2.33	2.04	0.44	0.81	0.051
[38]	pentane	methane	54.3	1.35	1.70	10.4	0.16	0.36	0.227
[39]	hexane	cyclohexane	0.70	0.15	0.33	0.53	0.11	0.27	0.007
[40]	hexane	cyclohexane	1.58	0.34	0.54	1.29	0.27	0.54	0.007
[41]	hexane	dodecane	0.82	0.18	0.42	0.56	0.12	0.28	0.006
[42]	hexane	carbon dioxide	28.23	2.62	5.28	1.37	0.15	0.33	0.186
[43]	hexane	ethanol	1.84	0.35	0.69	0.64	0.12	0.22	0.013
[44]	hexane	ethanol	1.66	0.31	0.69	0.77	0.14	0.27	0.013
[45]	hexane	ethanol	1.82	0.34	0.79	1.13	0.21	0.41	0.013
[46]	hexane	methanol	4.95	1.02	2.55	2.72	0.56	1.26	0.073
[47]	hexane	methanol	8.81	1.56	3.89	3.24	0.57	1.51	0.073
[48]	hexane	benzene	1.80	0.40	1.08	0.88	0.19	0.73	0.015
[40]	hexane	benzene	2.03	0.45	1.00	2.12	0.45	0.70	0.015
[49]	cyclohexane	decane	2.68	0.66	0.70	1.14	0.28	0.68	-0.017
[32]	cyclohexane	heptane	1.44	0.30	0.64	0.56	0.12	0.35	0.011
[50]	cyclohexane	isooctane	1.38	0.27	0.68	0.60	0.12	0.68	0.010
[46]	cyclohexane	ethanol	1.85	0.42	0.79	1.04	0.24	0.43	0.015
[29]	cyclohexane	ethanol	2.06	0.45	0.83	0.70	0.15	0.29	0.015
[32]	cyclohexane	acetone	0.80	0.18	0.45	0.60	0.13	0.36	0.002
[51]	cyclohexane	acetone	1.55	0.35	0.48	1.85	0.42	0.51	0.002
[23]	cyclohexane	benzene	0.95	0.24	0.37	1.04	0.27	0.41	0.001
[40]	cyclohexane	benzene	0.43	0.11	0.25	0.35	0.09	0.24	0.001
[52]	cyclohexane	benzene	0.85	0.22	0.34	0.88	0.23	0.38	0.001
[34]	cyclohexane	benzene	0.44	0.11	0.20	0.41	0.10	0.23	0.001
[25]	cyclohexane	benzene	0.45	0.12	0.32	0.38	0.10	0.28	0.001
[7]	cyclohexane	*o*-xylene	1.31	0.34	0.76	0.45	0.11	0.26	0.010
[53]	DEA	ethanol	4.78	1.47	3.67	1.83	0.64	1.44	0.046
[53]	DEA	methanol	1.97	0.66	2.68	1.80	0.63	2.00	0.013
[54]	DEA	water	4.59	2.79	6.17	3.07	1.87	5.17	0.037
[55]	DEA	water	4.58	2.58	4.87	2.55	1.40	3.33	0.037
[56]	DEA	water	7.25	3.99	4.46	0.63	0.34	0.69	0.037
[57]	DEA	water	4.21	2.51	5.93	2.91	1.74	4.93	0.037
[58]	DEA	water	7.25	3.99	4.46	0.63	0.34	0.69	0.037
[59]	DEA	water	3.76	2.33	3.24	0.57	0.35	0.79	0.037
[60]	DEA	water	4.99	2.99	6.74	3.73	2.23	5.79	0.037

Ref.	fluid 1	fluid 2	δ_{ij} = 0 results			Fitted results			
			AADP (%)	AAD (mN/m)	max AD (mN/m)	AAD P (%)	AAD (mN/m)	max AD (mN/m)	δ_{ij}
[61]	DEA	water	4.35	2.49	4.77	1.71	0.95	1.78	0.037
[62]	DEA	water	5.48	2.61	4.84	0.79	0.37	0.83	0.037
[63]	octane	isooctane	1.41	0.27	0.38	0.46	0.09	0.33	-0.010
[64]	octane	ethanol	0.82	0.17	0.49	0.82	0.17	0.28	0.008
[65]	octane	ethanol	1.32	0.26	0.77	0.76	0.15	0.61	0.008
[66]	octane	*o*-xylene	4.83	1.17	1.73	1.09	0.27	0.84	0.037
[12]	octane	DMC	5.22	1.11	2.22	2.41	0.52	1.23	0.036
[27]	nonane	benzene	1.52	0.35	0.77	0.57	0.14	0.34	0.012
[66]	nonane	*o*-xylene	4.59	1.14	1.68	0.92	0.23	0.52	0.034
[67]	methyl-palmitate	ethanol	8.08	1.80	1.80	0.00	0.00	0.00	0.089
[68]	dodecane	isooctane	1.22	0.26	0.47	0.16	0.03	0.08	-0.010
[69]	dodecane	hexadecane	3.04	0.40	0.92	2.86	0.28	0.74	0.011
[65]	dodecane	ethanol	1.43	0.33	0.85	0.92	0.21	0.42	0.010
[48]	dodecane	benzene	6.71	1.65	3.15	0.95	0.24	0.64	0.060
[70]	dodecane	methyl-cyclohexane	2.52	0.62	0.88	0.46	0.11	0.37	-0.018
[71]	dimethyl ether	propane	1.37	0.11	0.36	1.38	0.11	0.37	0.001
[72]	decane	carbon dioxide	472.	0.76	1.97	356.	0.43	1.08	0.081
[73]	decane	carbon dioxide	463.	0.93	1.74	364.	0.59	1.04	0.081
[74]	decane	heptane	1.86	0.39	0.61	0.90	0.19	0.61	-0.012
[49]	decane	isooctane	3.68	0.79	0.96	1.37	0.29	0.65	-0.026
[74]	decane	hexadecane	1.10	0.27	0.61	0.86	0.21	0.61	0.005
[75]	decane	docosane	6.23	1.51	2.00	0.68	0.17	0.46	0.036
[65]	decane	ethanol	0.88	0.19	0.53	0.76	0.16	0.40	0.004
[66]	decane	*o*-xylene	5.26	1.33	1.86	0.81	0.21	0.58	0.039
[12]	decane	DMC	3.40	0.78	1.62	2.04	0.47	0.91	0.023
[76]	hydrogen	deuterium	4.98	0.13	0.30	2.65	0.07	0.19	-0.033
[77]	hydrogen	argon	6.42	0.44	0.96	2.92	0.15	0.54	-0.678
[53]	MEA	ethanol	10.4	3.41	5.45	1.65	0.59	1.17	0.089
[78]	MEA	ethanol	11.0	3.71	5.44	1.27	0.44	0.73	0.089
[53]	MEA	methanol	7.99	2.78	4.84	1.32	0.53	1.06	0.063
[78]	MEA	methanol	6.75	2.43	3.72	1.13	0.39	0.71	0.063
[79]	MEA	water	2.32	1.38	2.20	0.46	0.28	0.71	0.041
[80]	MEA	water	7.29	4.14	6.70	2.77	1.63	3.79	0.041
[56]	MEA	water	10.4	5.72	6.03	2.05	1.12	1.48	0.041
[81]	MEA	water	3.32	1.91	3.07	2.35	1.32	2.85	0.041
[82]	MEA	water	4.92	2.45	2.59	2.87	1.43	1.46	0.041
[83]	MEA	water	4.28	2.46	3.39	2.56	1.48	2.18	0.041
[59]	MEA	water	10.7	6.41	7.10	5.91	3.53	4.13	0.041
[61]	MEA	water	6.90	3.95	6.72	3.52	2.00	3.49	0.041
[62]	MEA	water	4.14	2.17	3.66	1.19	0.62	1.40	0.041
[84]	heptane	isooctane	0.60	0.12	0.19	0.57	0.11	0.19	-0.010
[63]	heptane	isooctane	1.37	0.25	0.41	0.52	0.10	0.33	-0.010
[85]	heptane	hexadecane	0.81	0.18	0.28	1.06	0.23	0.44	0.015
[74]	heptane	hexadecane	1.37	0.31	0.64	1.43	0.32	0.91	0.015
[37]	heptane	hexadecane	3.06	0.69	0.91	0.65	0.14	0.29	0.015
[86]	heptane	docosane	5.72[c]	1.27	2.13	2.03[c]	0.49	1.70	0.030

Ref.	fluid 1	fluid 2	δ_{ij} = 0 results			Fitted results			
			AADP (%)	AAD (mN/m)	max AD (mN/m)	AAD P (%)	AAD (mN/m)	max AD (mN/m)	δ_{ij}
[43]	heptane	ethanol	5.76	1.16	2.15	2.03	0.41	0.64	0.045
[87]	heptane	ethanol	5.82	1.14	2.06	1.75	0.34	0.66	0.045
[34]	heptane	benzene	1.08	0.23	0.72	0.78	0.15	0.38	0.012
[88]	heptane	benzene	2.23	0.52	1.16	1.35	0.31	0.87	0.012
[25]	cyclopentane	benzene	1.63	0.40	0.54	0.22	0.06	0.14	0.011
[89]	R125	R143a	1.32	0.07	0.23	1.33	0.04	0.11	0.008
[90]	R125	R143a	0.98	0.05	0.13	0.98	0.06	0.16	0.008
[91]	R125	R143a	4.41	0.18	0.28	2.80	0.09	0.11	0.008
[89]	R125	R32	16.6	0.28	0.38	17.1	0.31	0.50	0.004
[90]	R125	R32	1.47	0.09	0.17	0.88	0.05	0.12	0.004
[92]	R125	R32	1.40	0.06	0.21	1.36	0.05	0.13	0.004
[89]	R125	R152a	4.98	0.34	0.68	1.92	0.07	0.19	0.020
[93]	R125	R152a	2.56	0.21	0.46	1.55	0.13	0.31	0.020
[89]	R125	R134a	1.13	0.07	0.25	0.99	0.06	0.18	0.004
[89]	R143a	R134a	5.49	0.34	0.69	5.07	0.31	0.61	0.002
[94]	R143a	R134a	0.98	0.06	0.22	1.34	0.09	0.27	0.002
[95]	R143a	R227ea	3.57	0.17	0.31	1.89	0.08	0.21	-0.013
[87]	isooctane	ethanol	0.86	0.16	0.45	0.66	0.13	0.32	-0.005
[68]	isooctane	benzene	5.39	1.13	1.81	0.48	0.10	0.35	0.041
[96]	isooctane	methyl-cyclohexane	0.56	0.12	0.25	0.53	0.11	0.25	0.001
[27]	hexadecane	benzene	1.08	0.28	0.69	1.01	0.26	0.55	0.005
[70]	hexadecane	methyl-cyclohexane	0.67	0.18	0.57	0.72	0.19	0.54	0.002
[97]	hexadecane	D4	5.39	1.13	2.12	1.90	0.39	0.72	0.045
[23]	diethyl ether	benzene	2.48	0.52	0.90	0.42	0.09	0.15	0.020
[98]	carbon monoxide	nitrogen	1.28	0.10	0.18	0.21	0.02	0.04	0.012
[34]	ethanol	methanol	0.37	0.08	0.18	0.21	0.04	0.18	-0.002
[23]	ethanol	acetone	1.08	0.25	0.56	0.82	0.19	0.36	-0.008
[99]	ethanol	benzene	2.98	0.69	1.59	1.63	0.37	1.16	0.020
[100]	ethanol	benzene	1.78	0.44	0.73	1.13	0.28	0.42	0.020
[101]	ethanol	benzene	4.27	1.02	1.29	2.05	0.49	0.95	0.020
[23]	ethanol	benzene	0.99	0.25	0.55	1.77	0.44	0.75	0.020
[46]	ethanol	benzene	1.43	0.36	0.61	1.07	0.27	0.42	0.020
[29]	ethanol	benzene	1.42	0.34	0.66	0.66	0.16	0.37	0.020
[102]	ethanol	water	36.4	14.49	22.22	10.7	5.07	9.16	0.304
[103]	ethanol	water	35.9	12.15	22.73	15.7	4.93	8.93	0.304
[104]	ethanol	water	35.1	10.96	25.27	15.7	4.68	10.67	0.304
[105]	ethanol	water	29.2	9.95	22.41	16.3	5.15	9.04	0.304
[106]	ethanol	water	41.1	13.32	22.52	21.9	6.50	9.93	0.304
[107]	ethanol	water	36.0	12.34	23.12	17.6	5.76	9.17	0.304
[108]	ethanol	water	31.9	10.43	21.77	19.7	5.78	8.91	0.304
[109]	ethanol	water	33.8	12.75	22.51	15.0	5.48	9.33	0.304
[110]	ethanol	water	25.3	9.57	25.04	12.4	4.57	9.46	0.304
[111]	ethanol	water	42.7	14.12	23.41	14.5	4.78	8.62	0.304
[112]	ethanol	water	29.3	9.03	21.92	14.2	4.05	8.26	0.304
[113]	ethanol	water	41.1	12.12	19.66	16.8	4.86	9.00	0.304

Ref.	fluid 1	fluid 2	δ_{ij} = 0 results			Fitted results			
			AADP (%)	AAD (mN/m)	max AD (mN/m)	AAD P (%)	AAD (mN/m)	max AD (mN/m)	δ_{ij}
[114]	ethanol	water	36.9	12.35	22.46	16.4	5.13	8.95	0.304
[115]	ethanol	water	25.0	7.40	22.44	18.6	4.87	8.97	0.304
[116]	ethanol	water	24.0	8.01	20.87	17.2	4.98	8.68	0.304
[117]	ethanol	water	50.5	22.35	22.35	29.1	12.90	12.90	0.304
[118]	ethanol	water	30.1	7.99	15.83	29.9	7.68	9.06	0.304
[119]	ethanol	water	34.4	14.82	22.04	14.3	6.40	9.21	0.304
[120]	ethanol	water	32.1	8.93	13.43	29.3	7.89	9.09	0.304
[121]	ethanol	water	7.64	5.08	6.00	7.26	4.83	5.58	0.304
[122]	ethanol	water	55.5	20.36	22.56	14.4	5.77	10.87	0.304
[123]	ethanol	heavy water	40.5	13.18	27.65	15.1	5.14	12.25	0.353
[14]	ethanol	o-xylene	0.83	0.21	0.45	0.56	0.15	0.21	0.005
[29]	ethanol	methyl-cyclohexane	1.82	0.39	0.83	1.07	0.23	0.41	0.015
[124]	ethanol	ethylene glycol	11.4	3.26	7.09	1.67	0.47	1.40	0.100
[125]	methanol	acetone	0.43	0.10	0.25	0.30	0.07	0.25	0.001
[31]	methanol	acetone	0.65	0.14	0.32	0.66	0.14	0.32	0.001
[101]	methanol	benzene	5.01[d]	1.26	2.04	1.59[c]	0.39	0.92	0.033
[30]	methanol	benzene	4.98	1.20	1.93	0.98	0.24	0.39	0.033
[102]	methanol	water	17.3	7.81	12.98	4.05	2.10	4.03	0.164
[104]	methanol	water	21.9	7.97	15.44	5.61	1.99	4.50	0.164
[107]	methanol	water	16.7	6.24	13.28	6.78	2.32	3.57	0.164
[110]	methanol	water	15.8	6.91	14.57	4.54	2.11	4.33	0.164
[126]	methanol	water	17.3	6.30	12.34	6.17	2.02	4.98	0.164
[111]	methanol	water	26.6	9.21	16.73	5.75	1.95	5.42	0.164
[113]	methanol	water	19.7	5.76	10.88	7.00	1.69	2.71	0.164
[114]	methanol	water	20.0	7.59	12.89	5.38	1.98	3.73	0.164
[116]	methanol	water	18.5	7.17	12.77	5.45	2.00	3.62	0.164
[123]	methanol	heavy water	23.3	9.19	14.99	7.22	2.85	5.58	0.184
[23]	acetone	benzene	0.81	0.21	0.37	0.43	0.11	0.22	0.003
[127]	acetone	benzene	1.12	0.28	0.77	1.35	0.34	0.77	0.003
[128]	acetone	benzene	0.88	0.22	0.76	0.69	0.17	0.64	0.003
[101]	acetone	water	63.6	21.29	40.51	14.6	5.01	11.38	0.433
[129]	acetone	water	81.8	27.18	35.71	20.3	6.54	11.73	0.433
[130]	acetone	water	46.7	18.95	38.86	12.9	5.95	10.56	0.433
[131]	acetone	water	49.0	16.91	37.28	15.1	5.80	11.60	0.433
[34]	acetone	water	71.8	23.05	37.42	15.8	4.80	8.38	0.433
[132]	acetone	water	53.3	17.13	36.20	15.6	5.30	12.96	0.433
[128]	acetone	water	58.6	18.27	37.18	17.2	5.13	12.11	0.433
[33]	acetone	water	57.2	17.78	37.11	15.2	4.46	8.84	0.433
[16]	acetone	*o*-xylene	8.78	2.39	3.16	1.06	0.29	0.62	-0.062
[128]	benzene	water	6.30	2.33	4.78	5.06	1.93	3.72	2.000
[25]	benzene	*o*-xylene	1.33	0.38	0.52	0.32	0.09	0.15	0.008
[133]	methane	ethane	8.05	0.56	2.47	3.29	0.19	1.70	0.074
[134]	methane	propane	5.69	0.12	0.24	5.67	0.12	0.24	-0.001
[135]	methane	nitrogen	8.85	1.02	2.24	3.75	0.43	0.88	0.067
[98]	methane	nitrogen	12.1	1.23	2.19	3.48	0.40	0.85	0.067
[136]	methane	krypton	1.30	0.19	0.31	0.35	0.05	0.18	0.008

Ref.	fluid 1	fluid 2	δ_{ij} = 0 results			Fitted results			
			AADP (%)	AAD (mN/m)	max AD (mN/m)	AAD P (%)	AAD (mN/m)	max AD (mN/m)	δ_{ij}
[135]	methane	argon	2.72	0.42	1.22	2.34	0.36	0.85	0.017
[137]	propane	R32	2.60	0.16	0.51	2.53	0.15	0.45	-0.010
[138]	propane	R152a	10.6	0.87	1.09	3.54	0.25	0.60	-0.050
[89]	R32	R134a	6.24	0.29	0.47	6.05	0.27	0.47	0.001
[139]	R32	R134a	0.79	0.06	0.25	0.83	0.06	0.23	0.001
[140]	R32	R1123	4.24	0.27	0.56	2.29	0.14	0.41	-0.021
[141]	R32	R227ea	3.90	0.19	0.43	2.58	0.12	0.36	-0.012
[142]	R32	R1234yf	12.4	0.26	0.47	10.4	0.19	0.42	-0.017
[140]	R32	R1234yf	4.46	0.21	0.42	3.45	0.18	0.43	-0.017
[143]	R32	R1234ze(E)	4.11	0.36	0.97	2.56	0.19	0.41	0.026
[142]	R32	R1234ze(E)	4.17	0.21	0.64	3.59	0.11	0.31	0.026
[89]	R152a	R134a	1.50	0.15	0.38	1.06	0.12	0.33	-0.006
[144]	R22	R115	29.9	2.39	2.39	0.00	0.00	0.00	-0.196
[145]	nitrogen	oxygen	3.16	0.48	1.41	4.43	0.57	1.26	0.055
[146]	nitrogen	oxygen	7.96	1.10	2.41	2.50	0.36	1.01	0.055
[147]	nitrogen	oxygen	2.99	0.18	0.68	3.85	0.17	0.49	0.055
[148]	helium	argon	13.1[e]	0.25	0.69	13.1[d]	0.25	0.69	0.000
[148]	neon	argon	7.82	0.22	1.47	8.13	0.23	1.46	0.048
[149]	nitrogen	helium	4.94[f]	0.16	1.67	4.94[e]	0.16	1.67	0.000
[135]	nitrogen	argon	2.02	0.24	0.50	1.82	0.21	0.41	0.012
[98]	nitrogen	argon	1.89	0.18	0.30	0.41	0.04	0.08	0.012
[150]	water	ethylene glycol	2.74	1.40	2.77	2.74	1.40	2.77	0.041
[151]	water	ethylene glycol	6.05	3.53	5.40	1.91	1.17	2.17	0.041
[152]	water	ethylene glycol	5.98	3.63	5.43	1.96	1.22	2.23	0.041
[153]	water	ethylene glycol	4.58	2.38	5.77	2.16	1.06	2.13	0.041
[154]	water	ethylene glycol	4.97	3.16	6.74	2.04	1.30	2.72	0.041
[155]	water	ethylene glycol	4.88	2.86	5.24	2.10	1.19	2.26	0.041
[156]	water	ethylene glycol	2.98	1.96	5.03	1.02	0.68	1.23	0.041
[157]	water	ethylene glycol	5.95	3.35	7.18	2.53	1.43	4.40	0.041
[158]	water	ethylene glycol	5.26	3.10	5.48	1.52	0.89	2.00	0.041
[159]	water	ethylene glycol	3.70	2.37	4.41	0.86	0.56	0.97	0.041
[145]	oxygen	argon	0.49	0.08	0.15	0.52	0.08	0.17	0.001
[160]	oxygen	argon	0.48	0.06	0.19	0.45	0.06	0.19	0.001
[161]	R134a	R1234yf	8.05	0.19	0.37	4.88	0.09	0.22	-0.025
[161]	R134a	R1234ze(E)	2.71	0.10	0.25	2.43	0.04	0.06	0.012
[162]	D4	MD4M	7.35	1.40	1.43	0.28	0.05	0.06	-0.047
[162]	D4	MD2M	3.26	0.60	0.66	0.43	0.08	0.12	-0.019
[163]	krypton	argon	2.63[g]	0.13	0.55	2.63[f]	0.13	0.55	0.000
[164]	krypton	argon	8.19	0.40	1.35	8.19	0.40	1.35	0.000
[162]	MD4M	D5	6.55	1.25	1.39	0.02	0.00	0.01	-0.039
[162]	MD3M	D5	4.56	0.86	0.86	0.00	0.00	0.00	-0.024
[140]	R1123	R1234yf	4.37	0.32	1.11	4.43	0.31	1.09	0.002

Omitted from statistics due to REFPROP calculation problems: [a] 3 points, [b]6 points, [c] 4 points, [d] 1 point, [e] 14 points, [f]15points, [g] 3 points.

References

1. I.H. Bell, J. Phys. Chem. Ref. Data 51, 013103 (2022) https://doi.org/10.1063/5.0086060
2. R. Beckmüller, M. Thol, I.H. Bell, E.W. Lemmon, R. Span, J. Phys. Chem. Ref. Data 50, 013102 (2021) https://doi.org/10.1063/5.0040533
3. T. Neumann, (Ruhr University Bochum, 2021), https://hss-opus.ub.ruhr-uni-bochum.de/opus4/frontdoor/deliver/index/docId/8564/file/diss.pdf
4. I.H. Bell, E.W. Lemmon, J. Chem. Eng. Data 61, 3752 (2016) https://doi.org/10.1021/acs.jced.6b00257
5. O. Kunz, W. Wagner, J. Chem. Eng. Data 57, 3032 (2012) https://doi.org/10.1021/je300655b
6. J. Tkaczuk, I.H. Bell, E.W. Lemmon, N. Luchier, F. Millet, J. Phys. Chem. Ref. Data 49, 023101 (2020) https://doi.org/10.1063/1.5142275
7. D.V.S. Jain, S. Singh, Indian J. Chem. 12, 714 (1974)
8. D.J.L. Prak, J.S. Cowart, A.M. McDaniel, P.C. Trulove, J. Chem. Eng. Data 59, 3571 (2014) https://doi.org/10.1021/je500498m
9. M.S. Dhillon, B.S. Mahl, Z. Phys. Chem. (Leipzig) 259, 249 (1978) https://doi.org/10.1515/zpch-1978-25933
10. B.S. Mahl, S.L. Chopra, P.P. Singh, Z. Phys. Chem. (Leipzig) 249, 337 (1972)
11. M. Domínguez-Peréz, E. Rilo, L. Segade, C. Franjo, O. Cabeza, J. Chem. Eng. Data 55, 1317 (2010) https://doi.org/10.1021/je900643r
12. A. Gayol, L.M. Casas, A.E. Andreatta, R.E. Martini, J.L. Legido, J. Chem. Eng. Data 58, 758 (2013) https://doi.org/10.1021/je301282p
13. L. Mosteiro, L.M. Casás, J.L. Legido, J. Chem. Thermodyn. 41, 695 (2009) https://doi.org/10.1016/j.jct.2008.12.010
14. G. Ouyang, G. Lu, Y. Yang, Z.Q. Huang, Acta Sci. Nat. Univ. Sunyatseni 43, 44 (2004)
15. R.K. Wanchoo, J. Narayan, Phys. Chem. Liq. 25, 15 (1992) https://doi.org/10.1080/00319109208027283
16. G. Ouyang, Y. Yang, S.S. Lu, Z.Q. Huang, B. Kang, J. Chem. Eng. Data 49, 330 (2004) https://doi.org/10.1021/je0341763
17. J.C.G. Calado, I.A. McLure, V.A.M. Soares, Fluid Phase Equilib. 2, 199 (1978) https://doi.org/10.1016/0378-3812(78)80009-0
18. J.J.-C. Hsu, N. Nagarajan, R.L. Robinson Jr, J. Chem. Eng. Data 30, 485 (1985) https://doi.org/10.1021/je00042a036
19. F. Gozalpour, A. Danesh, A.C. Todd, B. Tohidi, Fluid Phase Equilib. 233, 144 (2005) https://doi.org/10.1016/j.fluid.2005.03.032
20. R.P. Worley, J. Chem. Soc. 105, 273 (1914) https://doi.org/10.1039/CT9140500273
21. R. Tahery, J. Chem. Thermodyn. 106, 95 (2017) https://doi.org/10.1016/j.jct.2016.11.018
22. M. Dominguez-Perez, L. Segade, O. Cabeza, C. Franjo, E. Jimenez, J. Chem. Eng. Data 51, 294 (2006) https://doi.org/10.1021/je0504111
23. D.L. Hammick, L.W. Andrew, J. Chem. Soc., 754 (1929) https://doi.org/10.1039/JR9290000754
24. J.L.R. Morgan, M.A. Griggs, J. Am. Chem. Soc. 39, 2261 (1917) https://doi.org/10.1021/ja02256a002
25. V.T. Lam, G.C. Benson, Can. J. Chem. 48, 3773 (1970) https://doi.org/10.1139/v70-637
26. T.D. Ling, M. Van Winkle, Ind. Eng. Chem. Chem. Eng. Data Ser. 3, 82 (1958) https://doi.org/10.1021/i460003a017
27. G.R. Vakili-Nezhaad, M. Al-Wadhahi, S. Al-Haddabi, A. Vakilinejad, W.E. Acree, J. Chem. Thermodyn. 152, 106292 (2021) https://doi.org/10.1016/j.jct.2020.106292
28. H. Kahl, T. Wadewitz, J. Winkelmann, J. Chem. Eng. Data 48, 580 (2003) https://doi.org/10.1021/je0201323
29. R.S. Myers, H.L. Clever, J. Chem. Thermodyn. 6, 949 (1974) https://doi.org/10.1016/0021-9614(74)90216-x
30. S.S. Shastri, A.K. Mukherjee, T.R. Das, J. Chem. Eng. Data 38, 399 (1993) https://doi.org/10.1021/je00011a017

31. S. Singh, B.S. Lark, S.K. Aggarwal, Indian J. Chem., Sect. A: Inorg., Phys., Theor. Anal. 21, 1116 (1982)
32. H. Kahl, T. Wadewitz, J. Winkelmann, J. Chem. Eng. Data 48, 1500 (2003) https://doi.org/10.1021/je034062r
33. S. Enders, H. Kahl, J. Winkelmann, J. Chem. Eng. Data 52, 1072 (2007) https://doi.org/10.1021/je7000182
34. B.I. Konobeev, V.V. Lyapin, Zh. Prikl. Khim. 43, 803 (1970)
35. I.M. Abdulagatov, A.P. Adamov, I.M. Abdurakhmanov, J. Eng. Phys. Thermophys. 63, 1193 (1992) https://doi.org/10.1007/BF00853519
36. M. Mohsen-Nia, H. Rasa, S.F. Naghibi, J. Chem. Thermodyn. 42, 110 (2010) https://doi.org/10.1016/j.jct.2009.07.018
37. M. Mohsen-Nia, Phys. Chem. Liq. 49, 608 (2011) https://doi.org/10.1080/00319104.2010.489189
38. J. Satherley, D.L. Cooper, D.J. Schiffrin, Fluid Phase Equilib. 456, 193 (2018) https://doi.org/10.1016/j.fluid.2017.10.023
39. H.L. Clever, W.E. Chase, J. Chem. Eng. Data 8, 291 (1963) https://doi.org/10.1021/je60018a004
40. K. Ridgway, P.A. Butler, J. Chem. Eng. Data 12, 509 (1967) https://doi.org/10.1021/je60035a012
41. R.L. Schmidt, H.L. Clever, J. Colloid Interface Sci. 26, 19 (1968) https://doi.org/10.1016/0021-9797(68)90266-X
42. T.M. Koller, S. Yan, C. Steininger, T. Klein, A.P. Fröba, Int. J. Thermophys. 40, 79 (2019) https://doi.org/10.1007/s10765-019-2544-y
43. D. Papaioannou, C.G. Panayiotou, J. Chem. Eng. Data 39, 457 (1994) https://doi.org/10.1021/je00015a012
44. E. Jimenez Cuesta, H. Casad, L. Segade, C. Franjo, J. Chem. Eng. Data 45, 862 (2000) https://doi.org/10.1021/je000060k
45. B. Giner, A. Villares, S. Martin, H. Artigas, C. Lafuente, J. Chem. Eng. Data 52, 1904 (2007) https://doi.org/10.1021/je700215z
46. H.G. Trieschmann, Z. Phys. Chem. Abt. B 29, 328 (1935)
47. V. Ramakrishna, M. Patel, Indian J. Chem. 8, 256 (1970)
48. R.L. Schmidt, J.C. Randall, H.L. Clever, J. Phys. Chem. 70, 3912 (1966) https://doi.org/10.1021/j100884a027
49. D. Gomez-Diaz, J.C. Mejuto, J.M. Navaza, A. Rodriguez-Alvarez, J. Chem. Eng. Data 47, 872 (2002) https://doi.org/10.1021/je010288n
50. D. Gomez-Diaz, J.C. Mejuto, J.M. Navaza, J. Chem. Eng. Data 46, 720 (2001) https://doi.org/10.1021/je000310x
51. A. Mejia, H. Segura, M. Cartes, C. Calvo, Fluid Phase Equilib. 270, 75 (2008) https://doi.org/10.1016/j.fluid.2008.06.006
52. S.K. Suri, V. Ramakrishna, J. Phys. Chem. 72, 3073 (1968) https://doi.org/10.1021/j100855a001
53. M. Abroodi, A. Bagheri, B.M. Razavizadeh, J. Mol. Liq. 287, 110924 (2019) https://doi.org/10.1016/j.molliq.2019.110924
54. E.B. Rinker, D.W. Oelschlager, A.T. Colussi, K.R. Henry, O.C. Sandall, J. Chem. Eng. Data 39, 392 (1994) https://doi.org/10.1021/je00014a046
55. G. Vazquez, E. Alvarez, R. Rendo, E. Romero, J.M. Navaza, J. Chem. Eng. Data 41, 806 (1996) https://doi.org/10.1021/je960012t
56. E. Alvarez, R. Rendo, B. Sanjurjo, M. Sanchez-Vilas, J.M. Navaza, J. Chem. Eng. Data 43, 1027 (1998) https://doi.org/10.1021/je980106y
57. J. Aguila-Hernandez, A. Trejo Rodriguez, J. Gracia-Fadrique, Fluid Phase Equilib. 185, 165 (2001) https://doi.org/10.1016/s0378-3812(01)00467-8
58. E. Alvarez, A. Cancela, R. Maceiras, J.M. Navaza, R. Taboas, J. Chem. Eng. Data 48, 32 (2003) https://doi.org/10.1021/je020048n
59. D. Fu, L. Du, H. Wang, J. Chem. Thermodyn. 69, 132 (2014) https://doi.org/10.1016/j.jct.2013.10.016

60. A. Dey, S.K. Dash, B. Mandal, Fluid Phase Equilib. 463, 91 (2018) https://doi.org/10.1016/j.fluid.2018.01.030
61. D. Fu, J. Xie, F. Wang, S. Wang, J. Chem. Thermodyn. 116, 197 (2018) https://doi.org/10.1016/j.jct.2017.08.024
62. A. Shojaeian, J. Mol. Liq. 254, 26 (2018) https://doi.org/10.1016/j.molliq.2018.01.077
63. J.d.l.S. Lopez-Lazaro, G.A. Iglesias-Silva, A. Estrada-Baltazar, J. Baraja-Fernandez, J. Chem. Eng. Data 60, 1823 (2015) https://doi.org/10.1021/acs.jced.5b00009
64. L. Segade, J.J. de Llano, M. Dominguez-Perez, O. Cabeza, M. Cabanas, E. Jimenez, J. Chem. Eng. Data 48, 1251 (2003) https://doi.org/10.1021/je034053i
65. A. Mejia, M. Cartes, H. Segura, J. Chem. Thermodyn. 43, 1395 (2011) https://doi.org/10.1016/j.jct.2011.04.005
66. A.E. Andreatta, R.E. Martini, J.L. Legido, L. Casas, Int. J. Eng. Res. Sci. (IJOER) 2, 51 (2016)
67. E. Onder, N. Sarier, Thermochim. Acta 690, 178698 (2020) https://doi.org/10.1016/j.tca.2020.178698
68. H.B. Evans, H.L. Clever, J. Phys. Chem. 68, 3433 (1964) https://doi.org/10.1021/j100793a507
69. J. Yang, J. Wu, J. Chem. Thermodyn. 170, 106782 (2022) https://doi.org/10.1016/j.jct.2022.106782
70. D.J.L. Prak, A.L. Mungan, J.S. Cowart, P.C. Trulove, J. Chem. Eng. Data 63, 1642 (2018) https://doi.org/10.1021/acs.jced.8b00008
71. S. Bi, X. Li, G. Zhao, J. Wu, Fluid Phase Equilib. 298, 150 (2010) https://doi.org/10.1016/j.fluid.2010.07.026
72. N. Nagarajan, R.L. Robinson Jr, J. Chem. Eng. Data 31, 168 (1986) https://doi.org/10.1021/je00044a012
73. R.D. Shaver, R.L. Robinson Jr, K.A.M. Gasem, Fluid Phase Equilib. 179, 43 (2001) https://doi.org/10.1016/s0378-3812(00)00475-1
74. L.I. Rolo, A.I. Caco, A.J. Queimada, I.M. Marrucho, J.A.P. Coutinho, J. Chem. Eng. Data 47, 1442 (2002) https://doi.org/10.1021/je025536+
75. A.J. Queimada, A.I. Caco, I.M. Marrucho, J.A.P. Coutinho, J. Chem. Eng. Data 50, 1043 (2005) https://doi.org/10.1021/je050024r
76. V.N. Grigor'ev, N.S. Rudenko, Zh. Eksp. Teor. Fiz. 47, 92 (1964)
77. Y.P. Blagoi, G.P. Kropachev, V.V. Pashkov, Ukr. Fiz. Zh. 12, 1300 (1967)
78. M. Abroodi, A. Bagheri, B.M. Razavizadeh, J. Chem. Eng. Data 65, 3173 (2020) https://doi.org/10.1021/acs.jced.0c00192
79. Anonymous, Natural Gas Research Institute, Chem. Eng. Oil Gas, 1 (1981)
80. G. Vazquez, E. Alvarez, J.M. Navaza, R. Rendo, E. Romero, J. Chem. Eng. Data 42, 57 (1997) https://doi.org/10.1021/je960238w
81. J. Han, J. Jin, D.A. Eimer, M.C. Melaaen, J. Chem. Eng. Data 57, 1095 (2012) https://doi.org/10.1021/je2010038
82. S.A. Jayarathna, C.K. Jayarathna, D.A. Kottage, S. Dayarathna, D.A. Eimer, M.C. Melaaen, J. Chem. Eng. Data 58, 343 (2013) https://doi.org/10.1021/je300920t
83. S.A. Jayarathna, A. Weerasooriya, S. Dayarathna, D.A. Eimer, M.C. Melaaen, J. Chem. Eng. Data 58, 986 (2013) https://doi.org/10.1021/je301279x
84. D.J. Luning Prak, J.S. Cowart, P.C. Trulove, J. Chem. Eng. Data 59, 3842 (2014) https://doi.org/10.1021/je5007532
85. J. Koefoed, J.V. Villadsen, Acta Chem. Scand. 12, 1124 (1958)
86. A.J. Queimada, F.A.E. Silva, A.I. Caco, I.M. Marrucho, J.A.P. Coutinho, Fluid Phase Equilib. 214, 211 (2003) https://doi.org/10.1016/s0378-3812(03)00354-6
87. H. Yue, Z. Liu, J. Chem. Eng. Data 61, 1270 (2016) https://doi.org/10.1021/acs.jced.5b00903
88. H. Zhou, W. Zhu, Huaxue Gongcheng 21, 61 (1993)
89. R. Heide, J. Schenk, Forschungsrat Kaltetechnik E.V. Betimmung der Transportgroben von HFKW 115 103 708, Dresden (1996)
90. M. Okada, T. Shibata, Y. Sato, Y. Higashi, Int. J. Thermophys. 20, 119 (1999) https://doi.org/10.1023/a:1021482231102

91. A.P. Fröba, S. Will, A. Leipertz, Int. J. Thermophys. 22, 1349 (2001) https://doi.org/10.1023/a:1012832701996
92. Y.Y. Duan, H. Lin, Fluid Phase Equilib. 213, 89 (2003) https://doi.org/10.1016/s0378-3812(03)00236-x
93. S. Bi, G. Zhao, J. Wu, Fluid Phase Equilib. 287, 23 (2009) https://doi.org/10.1016/j.fluid.2009.09.005
94. H. Lin, Y.Y. Duan, J. Chem. Eng. Data 49, 372 (2004) https://doi.org/10.1021/je0342163
95. H. Lin, Y.Y. Duan, Int. J. Thermophys. 24, 1495 (2003) https://doi.org/10.1023/b:ijot.0000004090.64922.63
96. C. Zhang, G. Li, L. Yue, Y. Guo, W. Fang, J. Chem. Eng. Data 60, 2541 (2015) https://doi.org/10.1021/acs.jced.5b00105
97. R.S. Myers, H.L. Clever, J. Chem. Eng. Data 14, 161 (1969) https://doi.org/10.1021/je60041a014
98. F.B. Sprow, J.M. Prausnitz, Trans. Am. Inst. Chem. Eng. 62, 1105 (1965) https://doi.org/10.1039/TF9666201105
99. W. Ramsay, E. Aston, Trans. R. Irish Acad. 32, 93 (1902)
100. A. Ritzel, Z. Phys. Chem., Stoechiom. Verwandschaftsl. 60, 319 (1907)
101. J.L.R. Morgan, A.J. Scarlett, J. Am. Chem. Soc. 39, 2275 (1917) https://doi.org/10.1021/ja02256a003
102. J. Traube, J. Prakt. Chem. 31, 77 (1885)
103. M. Descude, J. Phys., Paris 2, 348 (1903)
104. J.L.R. Morgan, M. Neidle, J. Am. Chem. Soc. 35, 1856 (1913) https://doi.org/10.1021/ja02201a006
105. L.L. Bircumshaw, J. Chem. Soc. Trans. 121, 887 (1922) https://doi.org/10.1039/CT9222100887
106. R.C. Ernst, C.H. Watkins, H.H. Ruwe, J. Phys. Chem. 40, 627 (1936) https://doi.org/10.1021/j150374a008
107. S. Valentiner, H.W. Hohls, Z. Phys. 108, 101 (1937)
108. W.S. Bonnell, L. Byman, D.B. Keyes, Ind. Eng. Chem. 32, 532 (1940) https://doi.org/10.1021/ie50364a019
109. B. Stahlberger, A. Guyer, Helv. Chim. Acta 33, 243 (1950)
110. B.Y. Teitel'baum, T.A. Gortalova, E.E. Sidorova, Zh. Fiz. Khim. 25, 911 (1951)
111. Y.V. Efremov, Zh. Fiz. Khim. 42, 1906 (1968)
112. L.S.C. Wan, T. Jeyabalan, Chem. Pharm. Bull. 34, 4744 (1986) https://doi.org/10.1248/cpb.34.4744
113. M.A. Kalbassl, M.W. Biddulph, J. Chem. Eng. Data 33, 473 (1988) https://doi.org/10.1021/je00054a024
114. G. Vazquez, E. Alvarez, J.M. Navaza, J. Chem. Eng. Data 40, 611 (1995) https://doi.org/10.1021/je00019a016
115. R. Belda, J.V. Herraez, O. Diez, Phys. Chem. Liq. 43, 91 (2005) https://doi.org/10.1080/00319100512331327342
116. R.B. Maximino, Phys. Chem. Liq. 47, 475 (2009) https://doi.org/10.1080/00319100802241657
117. K. Ludzik, K. Kustrzepa, H. Piekarski, M. Jozwiak, J. Chem. Eng. Data 61, 1047 (2016) https://doi.org/10.1021/acs.jced.5b00485
118. D. Gonçalves, M.F. Paludetti, P.M. Florido, C. Tonetti, C.B. Gonçalves, C.E.C. Rodrigues, J. Chem. Eng. Data 63, 2718 (2018) https://doi.org/10.1021/acs.jced.8b00086
119. M.A. Raza, P.D. Hallett, X. Liu, M. He, W. Afzal, J. Chem. Eng. Data 64, 5049 (2019) https://doi.org/10.1021/acs.jced.9b00026
120. D. Gonçalves, C. Panzarin, C.B. Gonçalves, C.E.C. Rodrigues, Fluid Phase Equilib. 521, 112730 (2020) https://doi.org/10.1016/j.fluid.2020.112730
121. S. Khosharay, M. Rahmanzadeh, B. ZareNezhad, Int. J. Thermophys. 41, 166 (2020) https://doi.org/10.1007/s10765-020-02738-0
122. D. Gomez-Diaz, J.M. Navaza, J. Chem. Eng. Data 66, 2160 (2021) https://doi.org/10.1021/acs.jced.1c00061

123. A. Bagheri, A.H. Amiri-Majed, J. Chem. Thermodyn. 51, 45 (2012) https://doi.org/10.1016/j.jct.2012.02.017
124. S. Azizian, M. Hemmati, J. Chem. Eng. Data 48, 662 (2003) https://doi.org/10.1021/je025639s
125. R.H. Campbell, E.M. Kartzmark, J. Chem. Thermodyn. 5, 163 (1973) https://doi.org/10.1016/s0021-9614(73)80076-x
126. S.I. Uchida, K. Matsumoto, Kagaku Kogaku 22, 570 (1958)
127. W.E. Shipp, J. Chem. Eng. Data 15, 308 (1970) https://doi.org/10.1021/je60045a020
128. Z.M. Rong, Y. Lu, Huaxue Gongcheng 16, 56 (1988)
129. R.C. Ernst, E.E. Litkenhous, J.W. Spanyer, J. Phys. Chem. 36, 842 (1932) https://doi.org/10.1021/j150333a006
130. B.Y. Teitel'baum, S.G. Ganelina, T.A. Gortalova, Zh. Fiz. Khim. 25, 1043 (1951)
131. K.S. Howard, R.A. McAllister, AIChE J. 3, 325 (1957) https://doi.org/10.1002/aic.690030308
132. A.I. Toryanik, V.G. Pogrebnik, J. Struct. Chem. 17, 464 (1976) https://doi.org/10.1007/BF00746671
133. V.G. Baidakov, A.M. Kaverin, M.N. Khotienkova, Fluid Phase Equilib. 356, 90 (2013) https://doi.org/10.1016/j.fluid.2013.07.008
134. K.N. Seneviratne, T.J. Hughes, M.L. Johns, K.N. Marsh, E.F. May, J. Chem. Thermodyn. 111, 173 (2017) https://doi.org/10.1016/j.jct.2017.03.002
135. Y.P. Blagoi, Ukr. Fiz. Zh. 5, 109 (1960)
136. S. Fuks, A. Bellemans, Physica 32, 594 (1966) https://doi.org/10.1016/0031-8914(66)90049-8
137. K. Tanaka, Y. Higashi, J. Chem. Eng. Data 54, 1656 (2009) https://doi.org/10.1021/je800756r
138. G. Zhao, S. Bi, J. Wu, Z. Liu, J. Chem. Eng. Data 55, 3077 (2010) https://doi.org/10.1021/je901085t
139. Y.Y. Duan, L. Hong, J. Chem. Eng. Data 48, 1068 (2003) https://doi.org/10.1021/je030137f
140. Y. Liu, C. Kondou, C. Coquelet, C. Houriez, Int. J. Refrig. 132, 276 (2021) https://doi.org/10.1016/j.ijrefrig.2021.09.021
141. H. Lin, Y.Y. Duan, J. Chem. Eng. Data 50, 182 (2005) https://doi.org/10.1021/je049743g
142. J. Cui, S. Bi, X. Meng, J. Wu, J. Chem. Eng. Data 61, 950 (2016) https://doi.org/10.1021/acs.jced.5b00798
143. K. Tanaka, Y. Higashi, J. Chem. Eng. Jpn. 46, 371 (2013) https://doi.org/10.1252/jcej.13we021
144. Anonymous, E.I. Du Pont De Nemours & CO. (INC.) Wilmington, Delaware (1969)
145. Y.P. Blagoi, N.S. Rudenko, Izv. Vyssh. Uchebn. Zaved., Fiz., 22 (1959)
146. V.B. Ostromukhov, M.G. Ostronov, Zh. Fiz. Khim. 68, 39 (1994)
147. V.G. Baidakov, A.M. Kaverin, V.N. Andbaeva, Fluid Phase Equilib. 270, 116 (2008) https://doi.org/10.1016/j.fluid.2008.06.016
148. A.M. Kaverin, V.N. Andbaeva, V.G. Baidakov, Zh. Fiz. Khim. 80, 495 (2006) https://doi.org/10.1134/s0036024406030174
149. V.G. Baidakov, A.M. Kaverin, Zh. Fiz. Khim. 78, 1150 (2004)
150. K.A. Clendenning, Can. J. Res. F 24, 249 (1946)
151. K. Nakanishi, T. Matsumoto, M. Hayatsu, J. Chem. Eng. Data 16, 44 (1971) https://doi.org/10.1021/je60048a010
152. Y.S. Won, D.K. Chung, A.F. Mills, J. Chem. Eng. Data 26, 140 (1981) https://doi.org/10.1021/je00024a011
153. B.C. Hoke, J.C. Chen, J. Chem. Eng. Data 36, 322 (1991) https://doi.org/10.1021/je00003a019
154. A. Horibe, S. Fukusako, M. Yamada, Int. J. Thermophys. 17, 483 (1996) https://doi.org/10.1007/bf01443405
155. N.G. Tsierkezos, I.E. Molinou, J. Chem. Eng. Data 43, 989 (1998) https://doi.org/10.1021/je9800914
156. K. Habrdova, S. Hovorka, L. Bartovska, J. Chem. Eng. Data 49, 1003 (2004) https://doi.org/10.1021/je049955d
157. J. Zhang, P. Zhang, F. Han, G. Chen, R. Deng, X.H. Wei, J. Chem. Eng. Data 53, 2372 (2008) https://doi.org/10.1021/je800271e

158. A.A. Rafati, A. Bagheri, M. Najafi, J. Chem. Thermodyn. 43, 248 (2011) https://doi.org/10.1016/j.jct.2010.09.003
159. A.K. Tiwari, Sonu, S.K. Saha, J. Chem. Thermodyn. 70, 24 (2014) https://doi.org/10.1016/j.jct.2013.10.010
160. Y. Saji, T. Okuda, Adv. Cryog. Eng. 10, 209 (1965)
161. S. Bi, J. Cui, G. Zhao, J. Wu, Fluid Phase Equilib. 414, 60 (2016) https://doi.org/10.1016/j.fluid.2016.01.013
162. H.I. Waterman, W.R.E. van Herwijnen, H.W. den Hartog, J. Appl. Chem. (London) 8, 625 (1958) https://doi.org/10.1002/jctb.5010081001
163. K.C. Nadler, Ph.D. Thesis (Cornell University), (1987)
164. I.I. Sulla, V.G. Baidakov, Zh. Fiz. Khim. 68, 63 (1994)
