# Supplementary material for: Assessment of a Parachor Model for the Surface Tension of Binary Mixtures
Source: Int J Thermophys. 2023 Jun 7;44(7):110. doi: 10.1007/s10765-023-03216-z (PMC10247860; doi:10.1007/s10765-023-03216-z)
Supplement: Supplementary file 3 — Supplementary file3 (PDF 2214 KB) [file 10765_2023_3216_MOESM3_ESM.pdf]

Supplementary Figures – Assessment of a Parachor Model for the Surface Tension of Binary Mixtures

Alexandra Metallinou Log, Vladimir Diky and Marcia L. Huber

May 11, 2023

In the following document, we present figures showing the deviation of the parachor method's surface tension estimate from experimentally measured results. Two figures are shown for each mixture, one with the binary interaction coefficient δ_{ij} set to zero, and another with the fitted binary interaction coefficient. A total of 154 binary mixtures are considered, and they are grouped into mixtures with alkanes, alcohols, water, aromatics, halocarbons and miscellaneous mixtures.

1 Alcohol mixtures

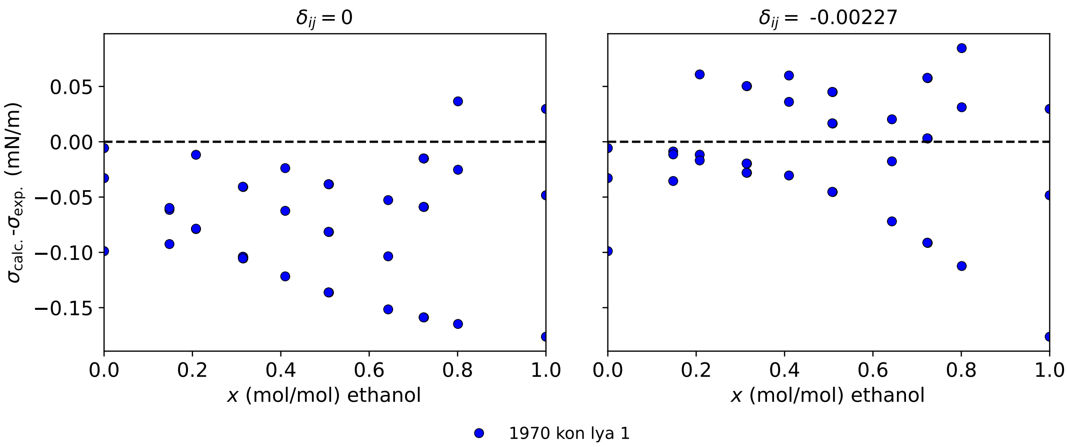

Figure 1: ethanol/methanol

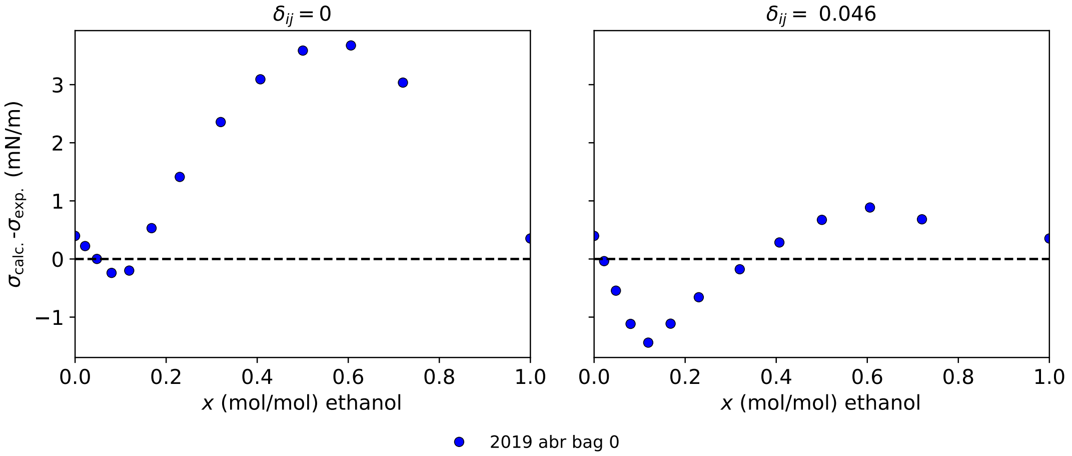

Figure 2: ethanol/DEA

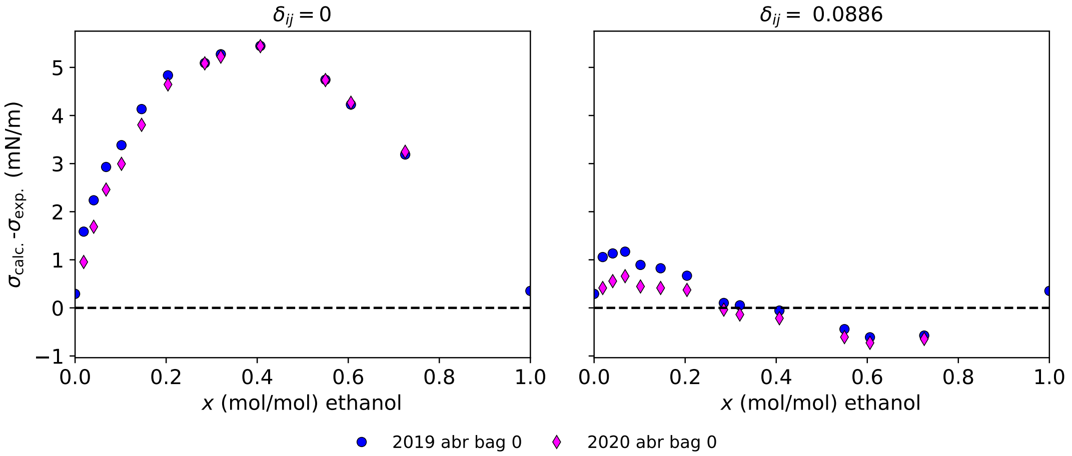

Figure 3: ethanol/MEA

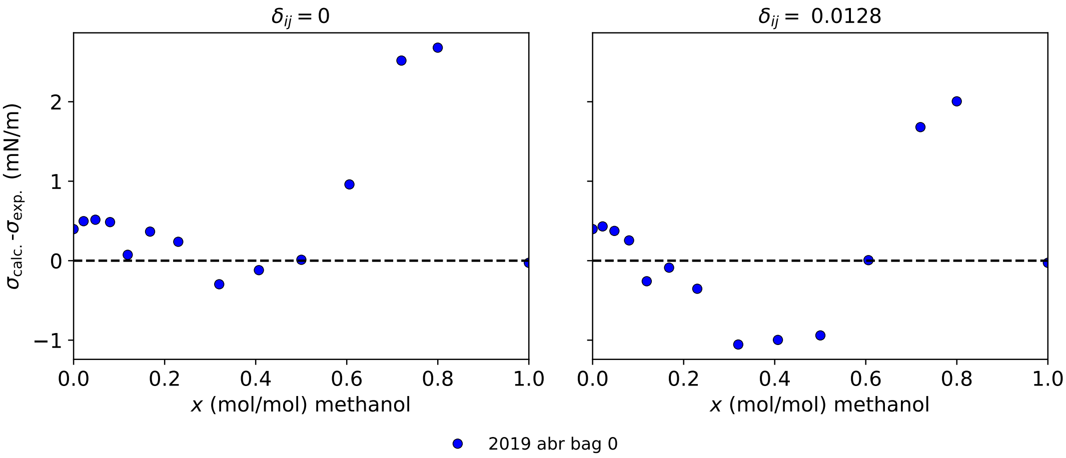

Figure 4: methanol/DEA

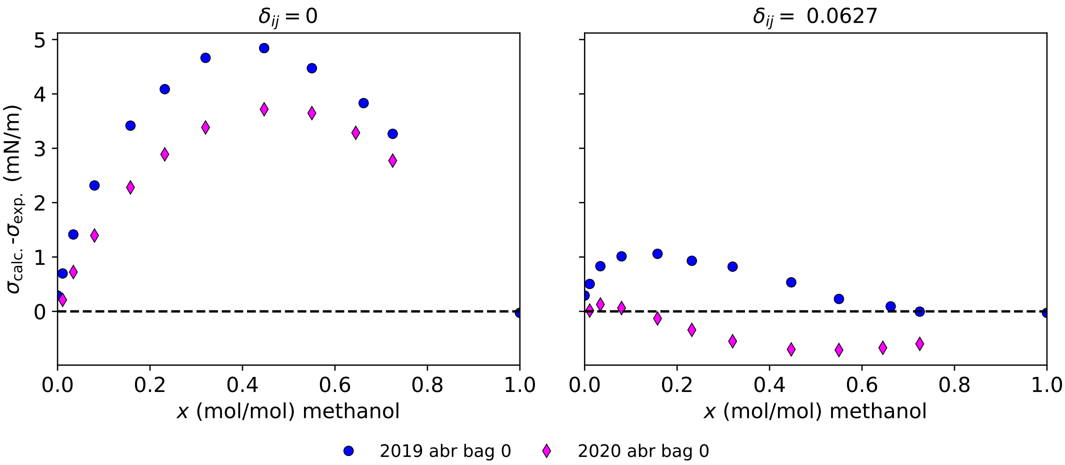

Figure 5: methanol/MEA

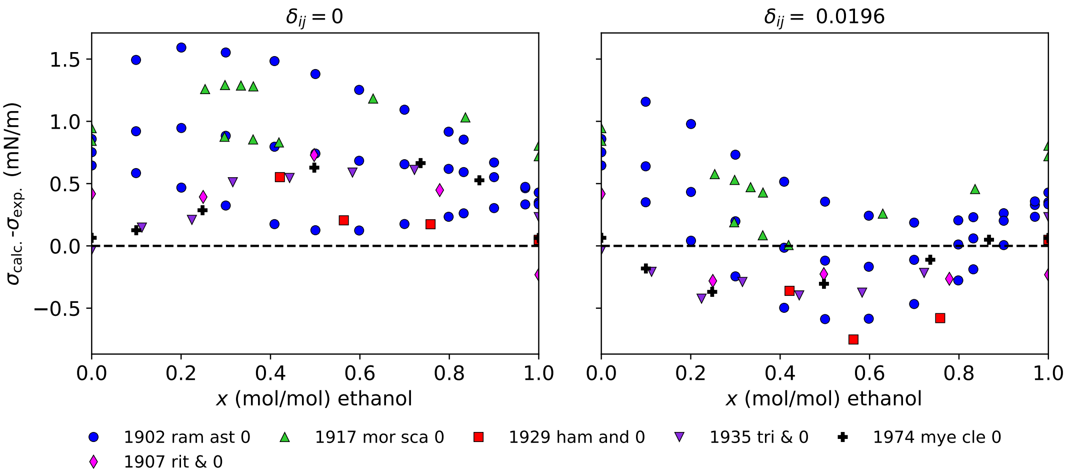

Figure 6: ethanol/benzene

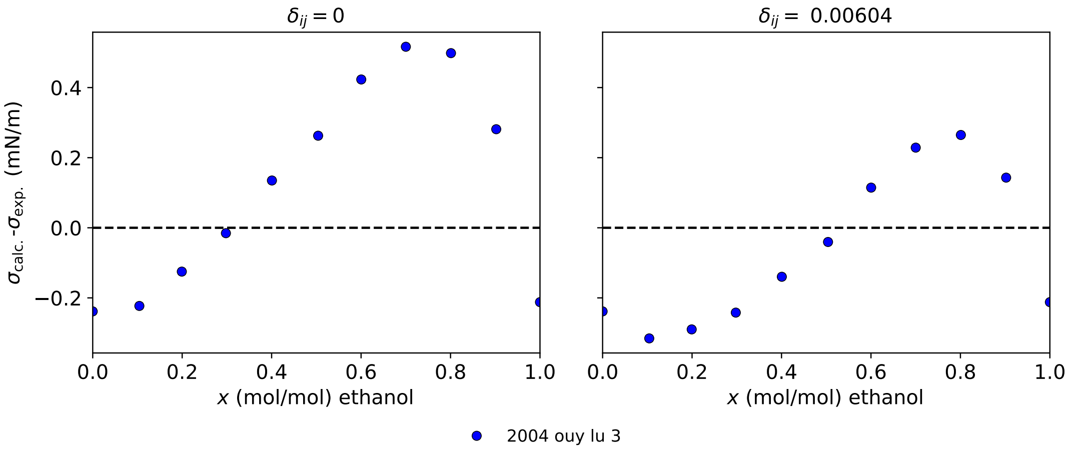

Figure 7: ethanol/m-xylene

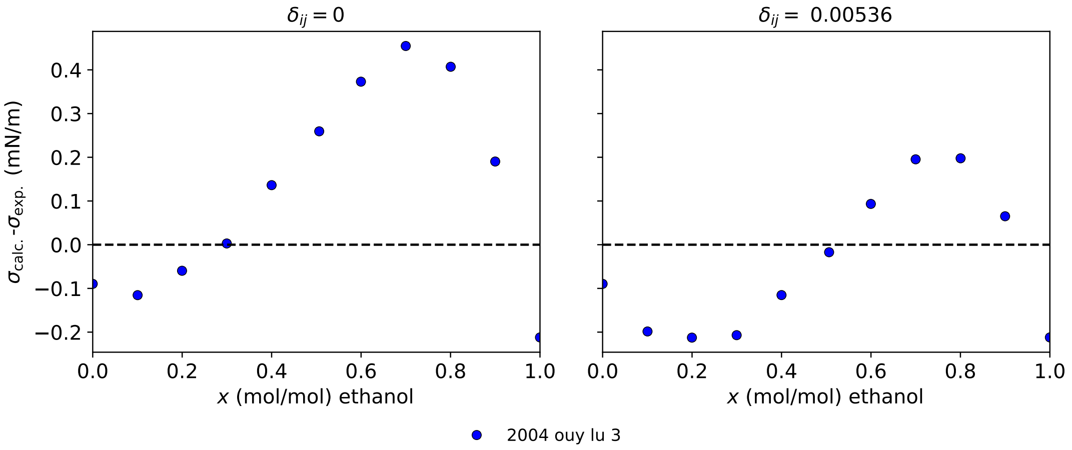

Figure 8: ethanol/o-xylene

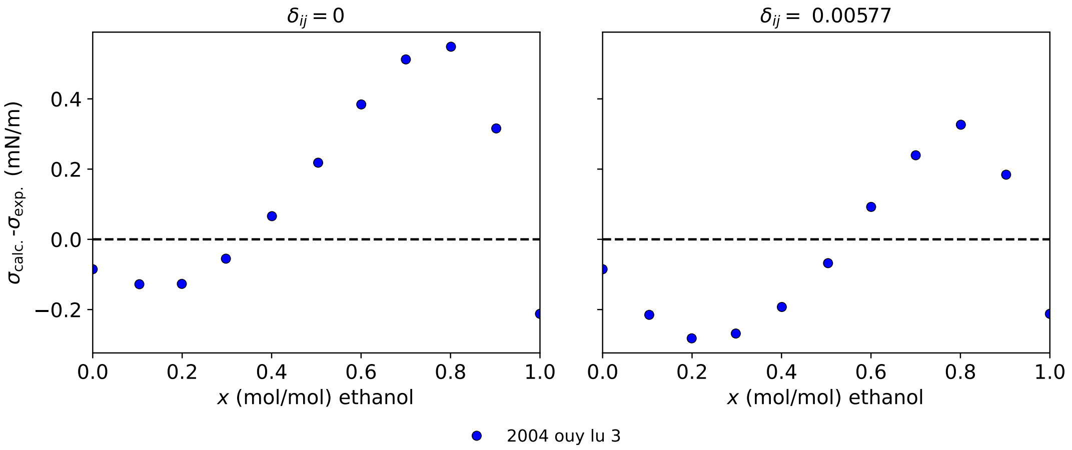

Figure 9: ethanol/p-xylene

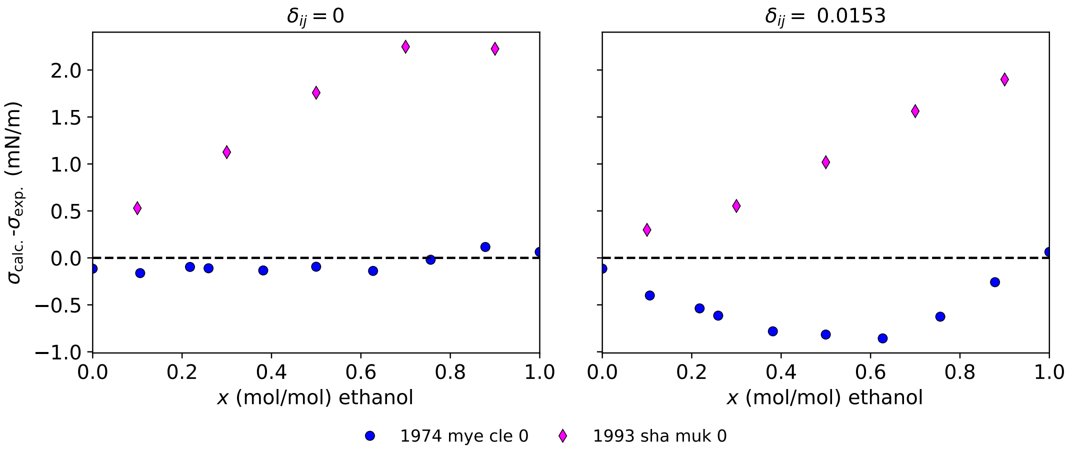

Figure 10: ethanol/toluene

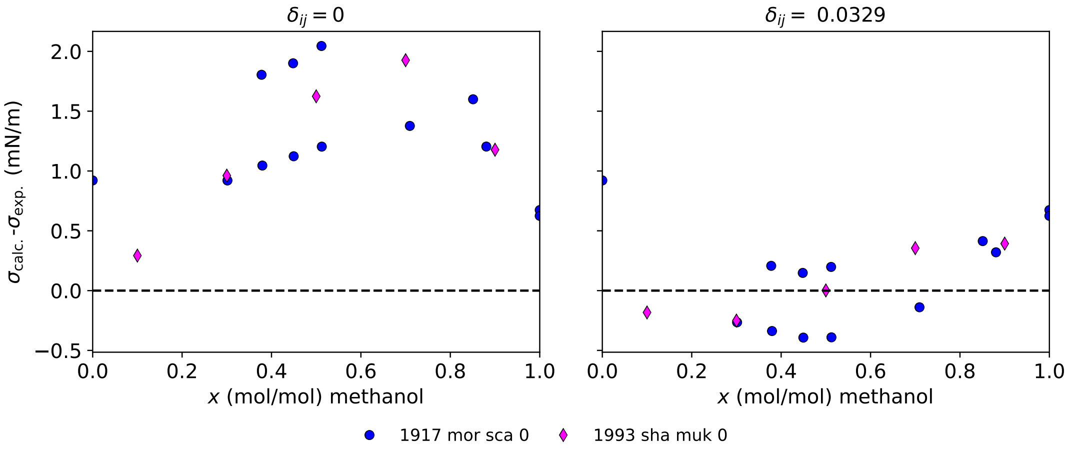

Figure 11: methanol/benzene

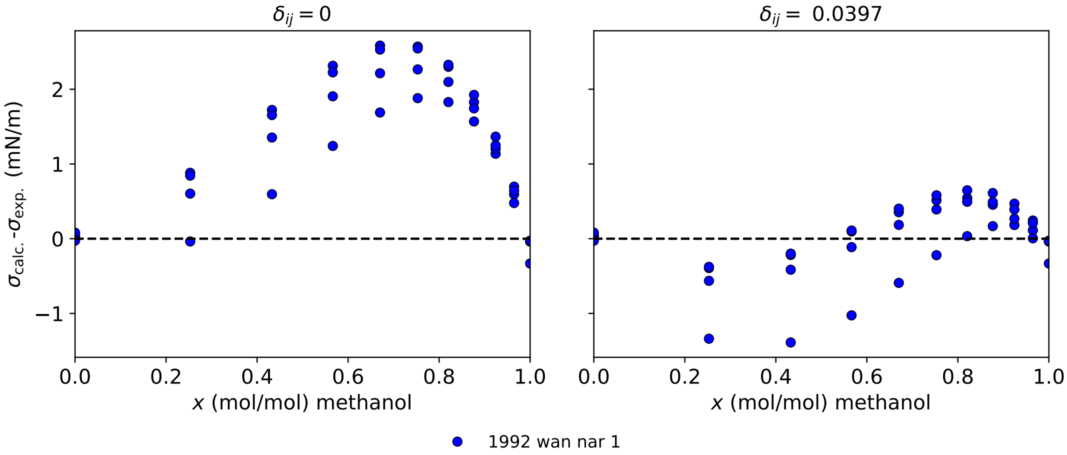

Figure 12: methanol/p-xylene

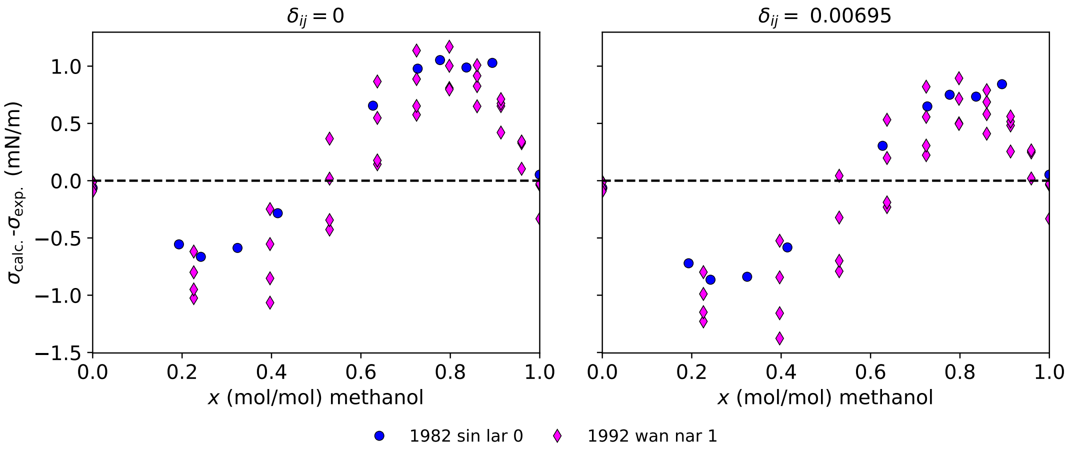

Figure 13: methanol/toluene

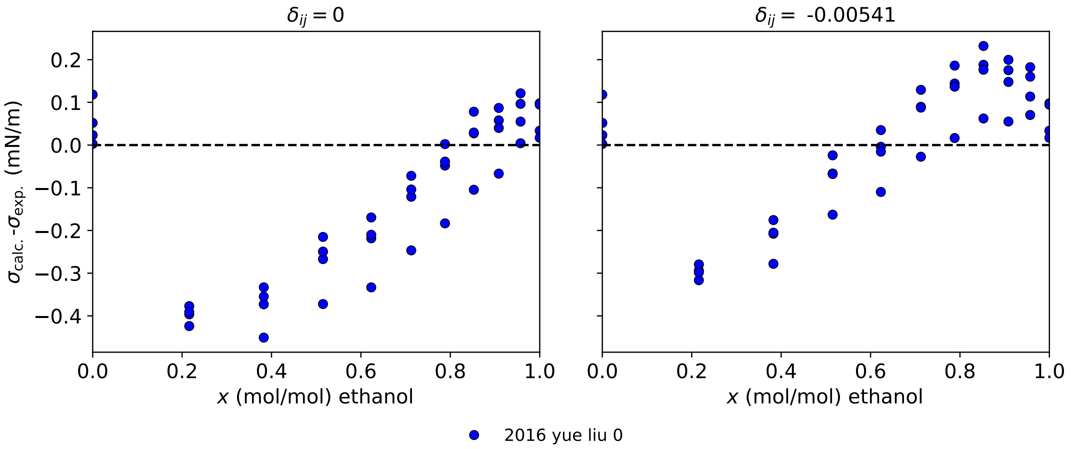

Figure 14: ethanol/isooctane

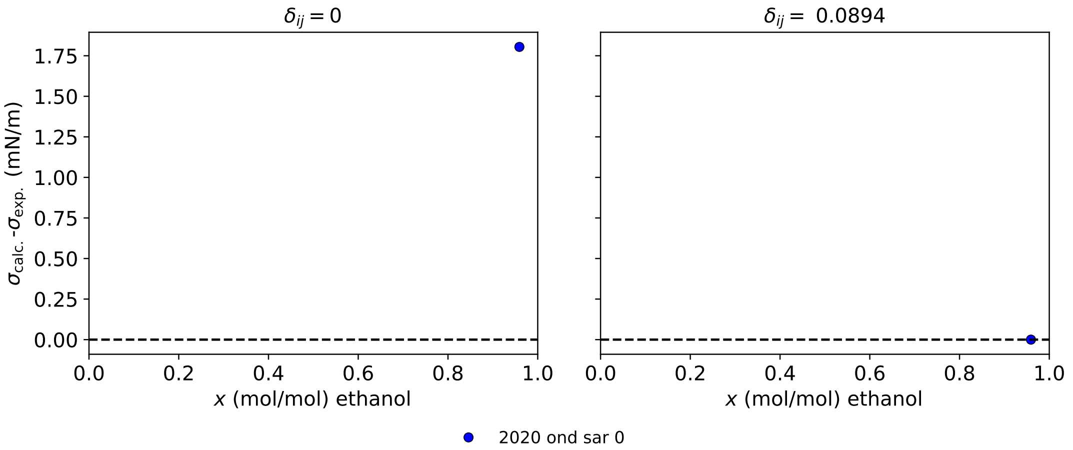

Figure 15: ethanol/methyl palmitate

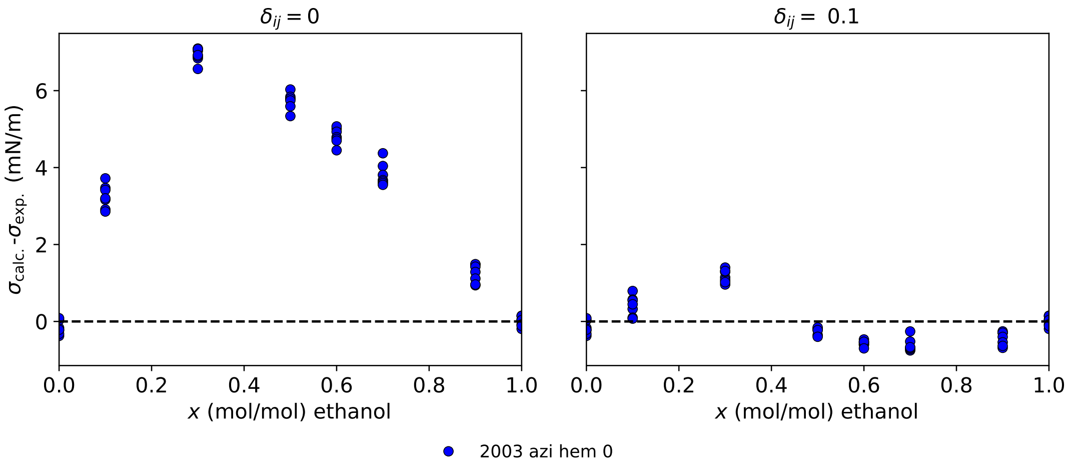

Figure 16: ethanol/ethylene glycol

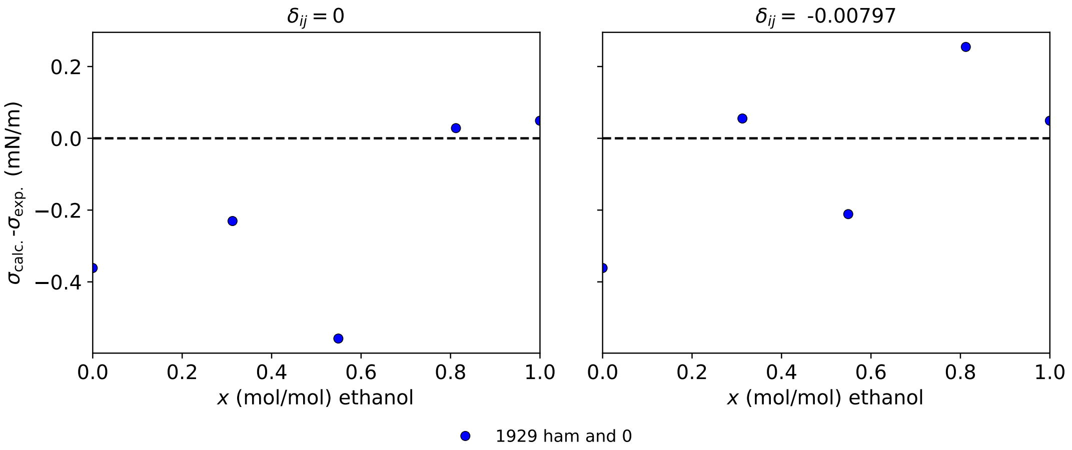

Figure 17: ethanol/acetone

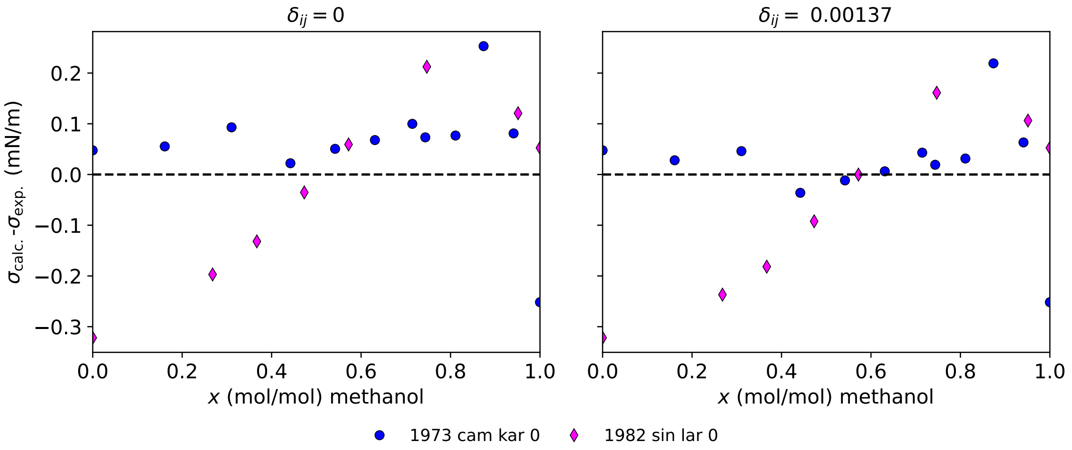

Figure 18: methanol/acetone

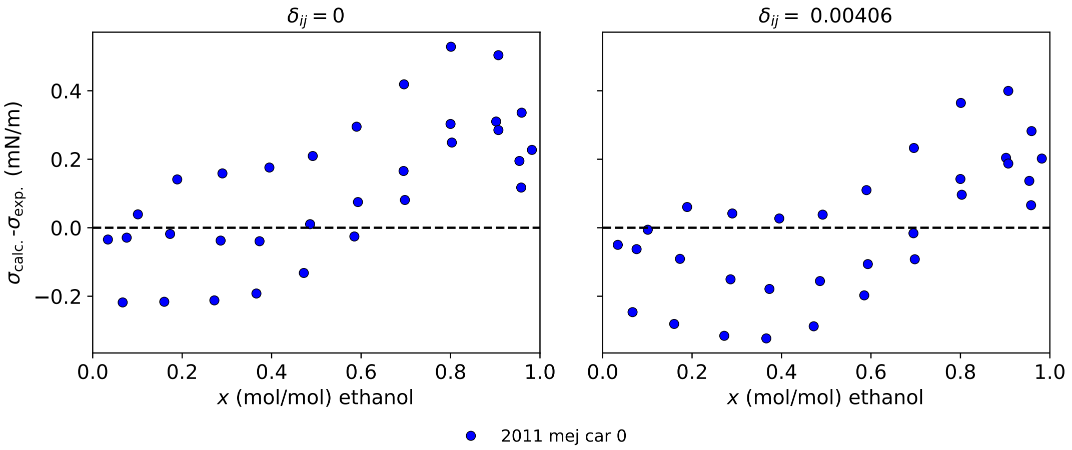

Figure 19: ethanol/decane

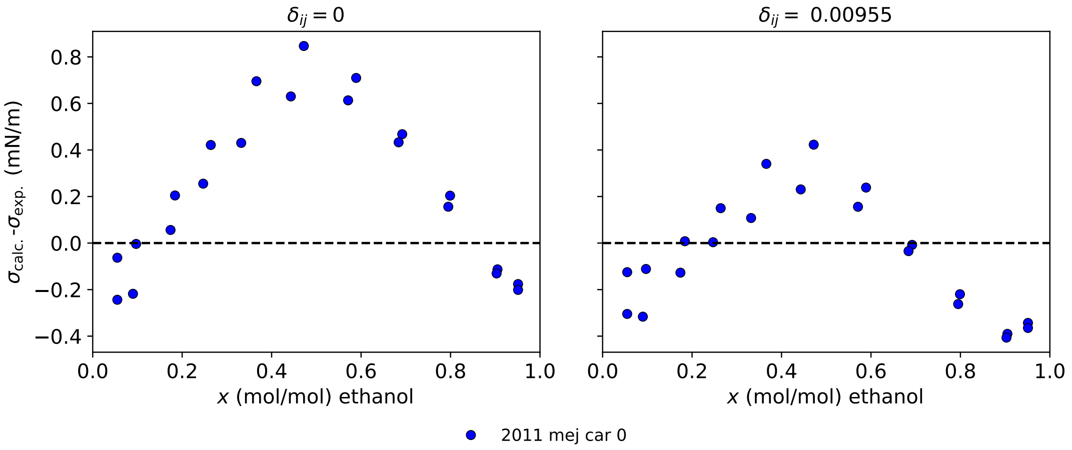

Figure 20: ethanol/dodecane

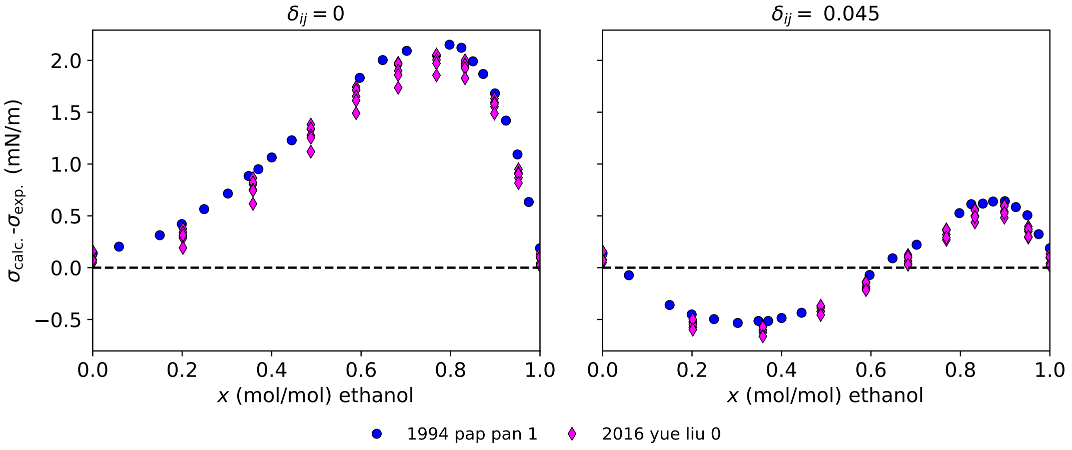

Figure 21: ethanol/heptane

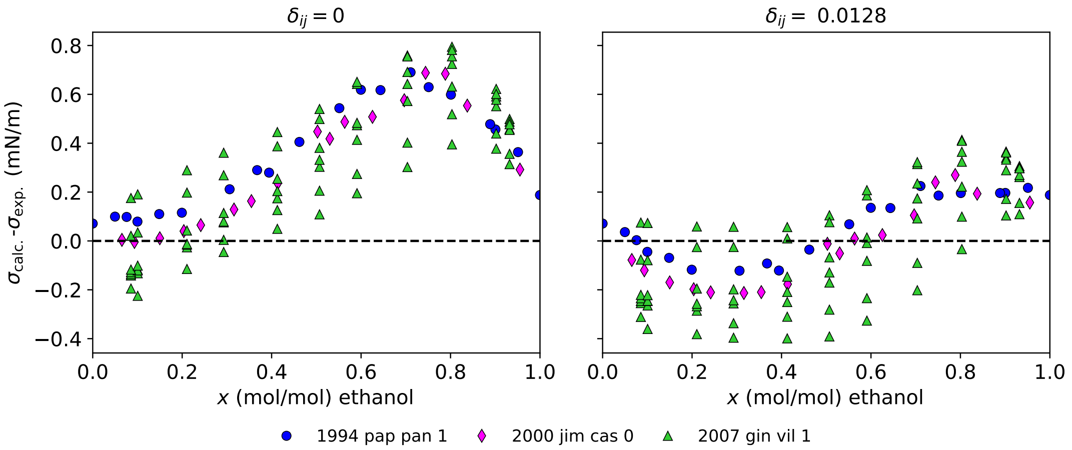

Figure 22: ethanol/hexane

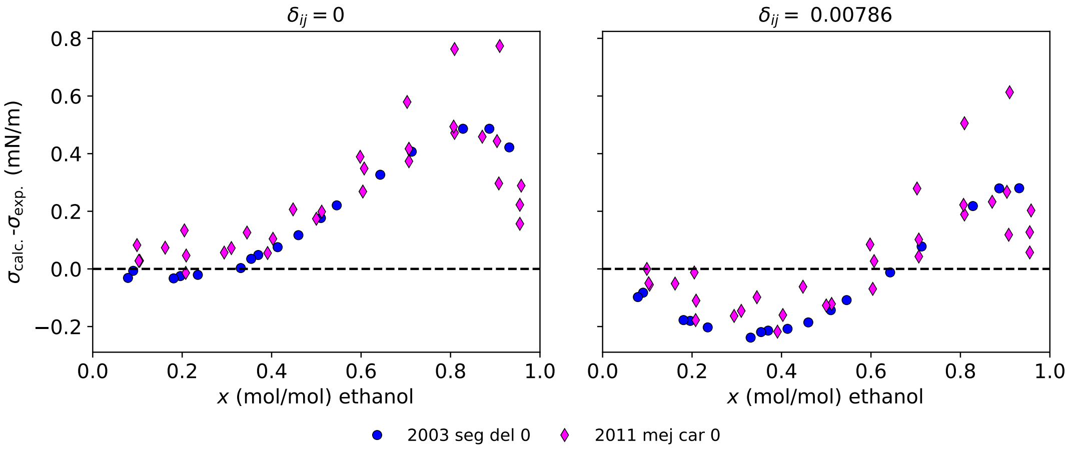

Figure 23: ethanol/octane

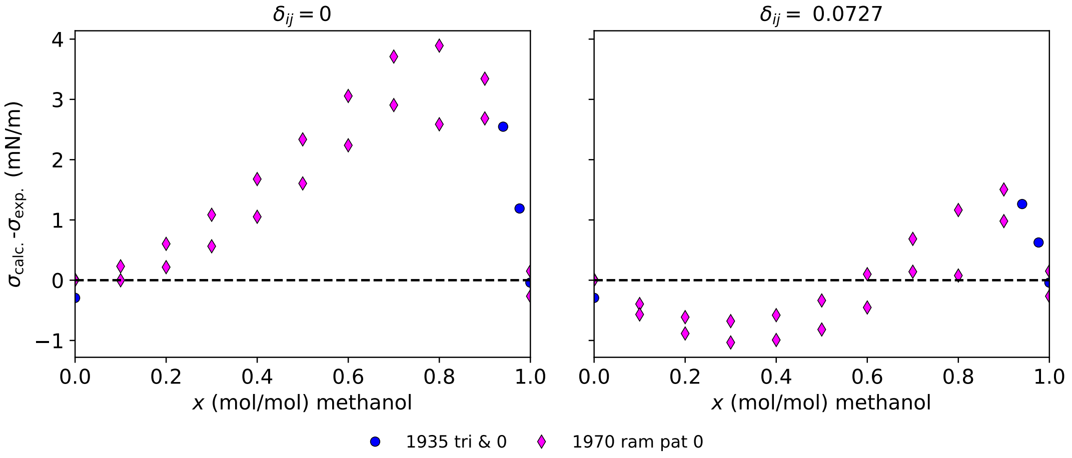

Figure 24: methanol/hexane

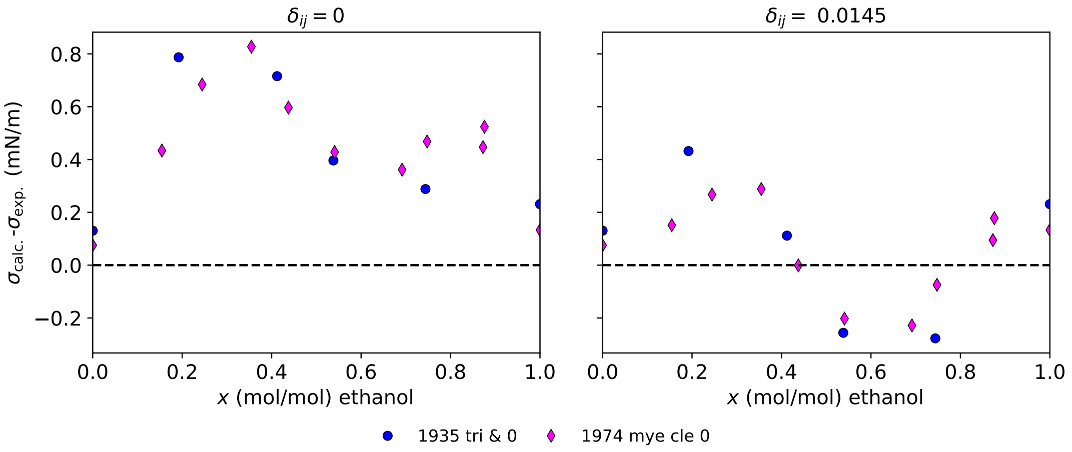

Figure 25: ethanol/cyclohexane

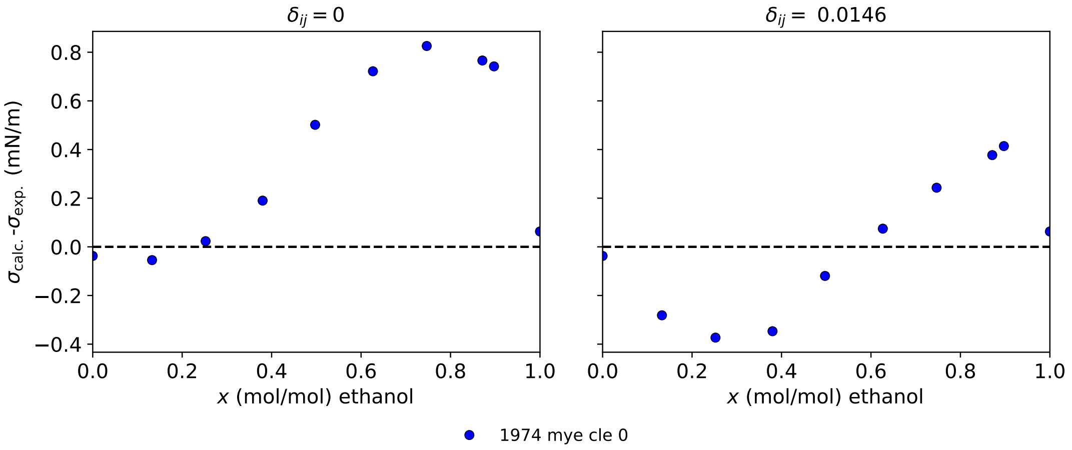

Figure 26: ethanol/methylcyclohexane

2 Aromatic mixtures

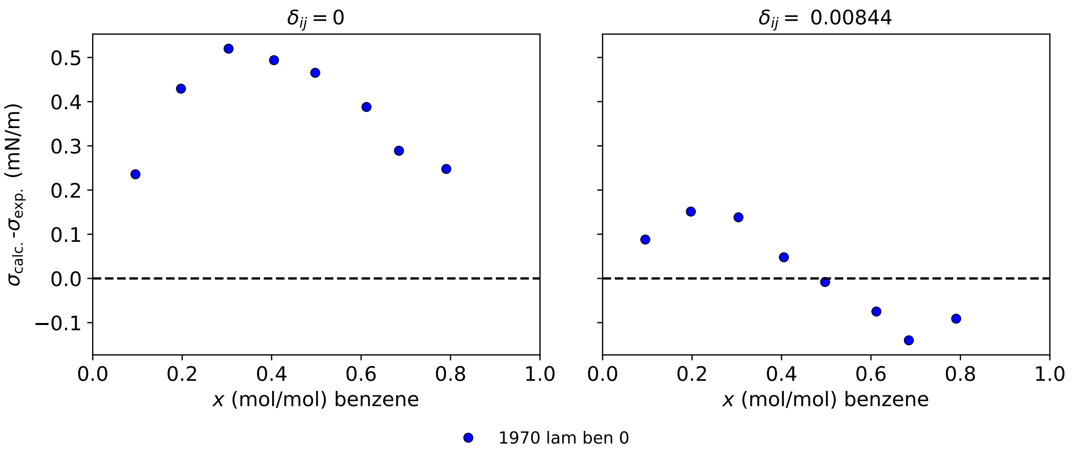

Figure 27: benzene/o-xylene

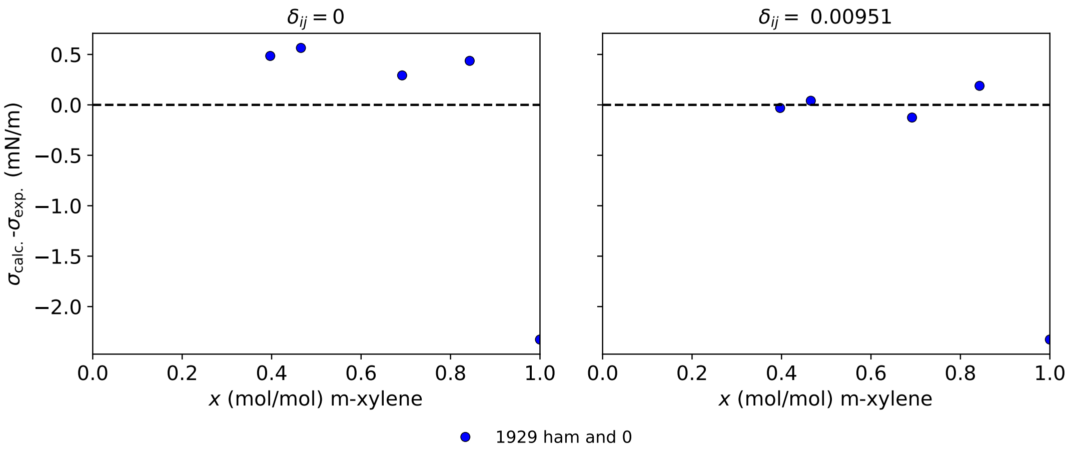

Figure 28: m-xylene/benzene

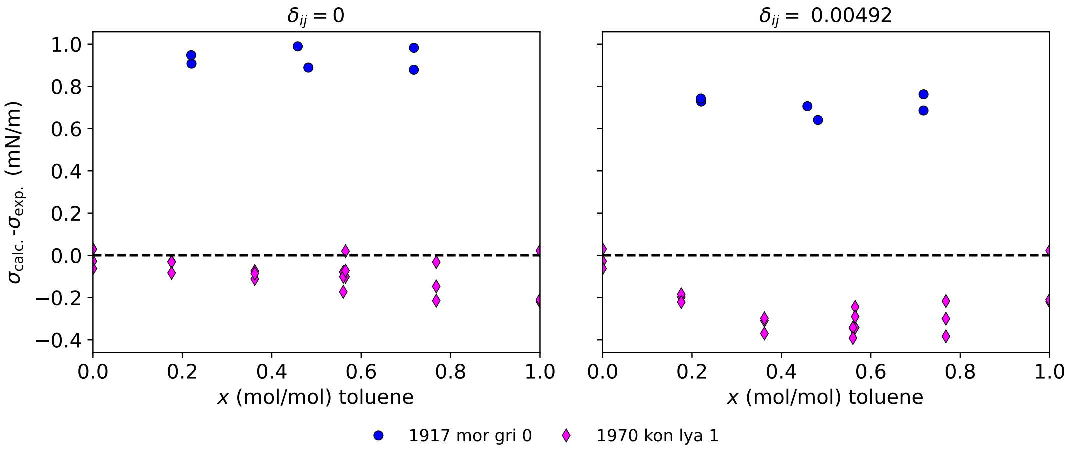

Figure 29: toluene/benzene

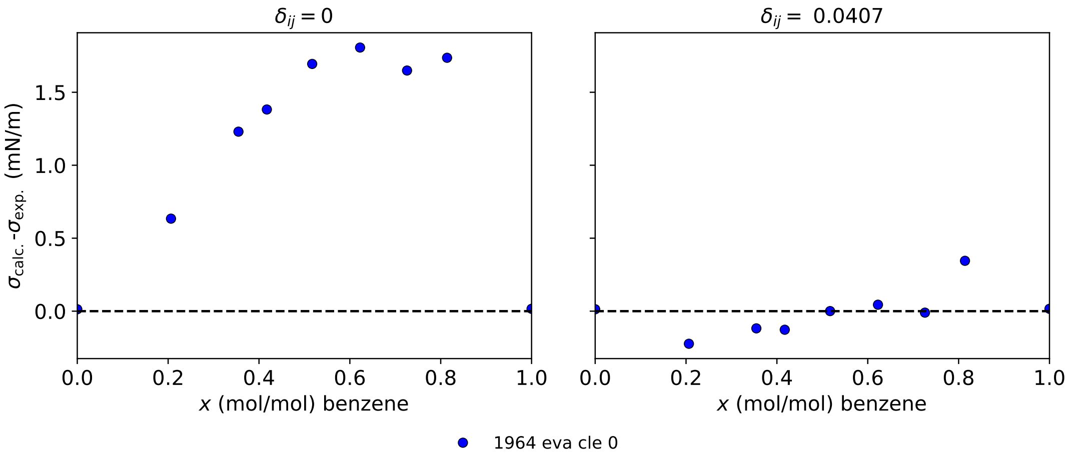

Figure 30: benzene/isooctane

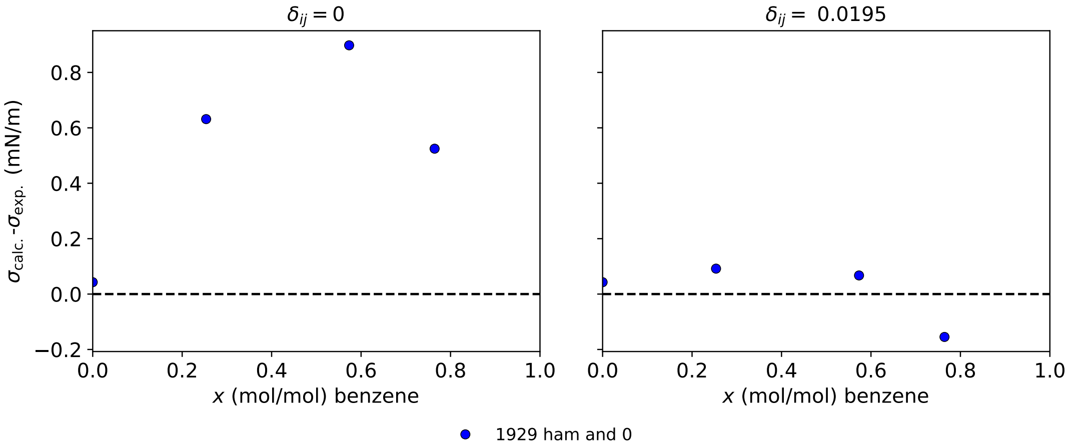

Figure 31: benzene/diethyl ether

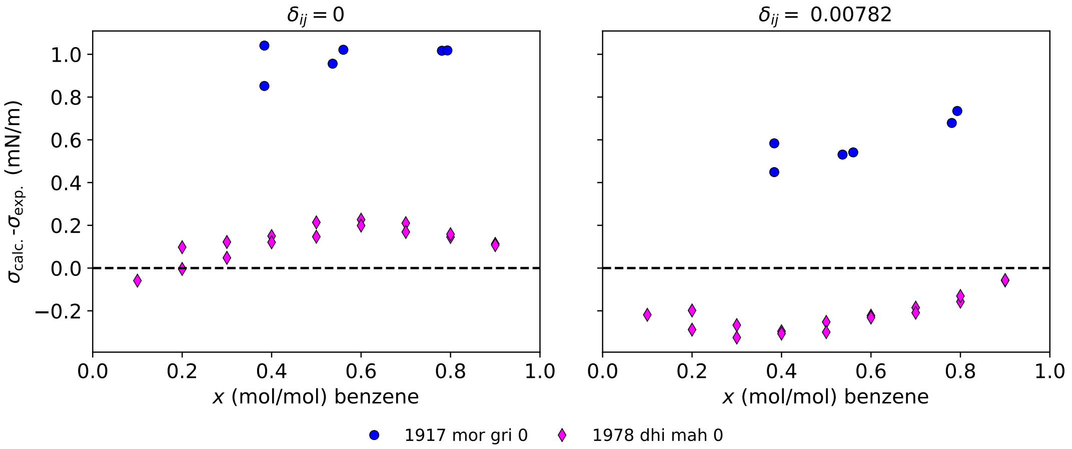

Figure 32: benzene/chlorobenzene

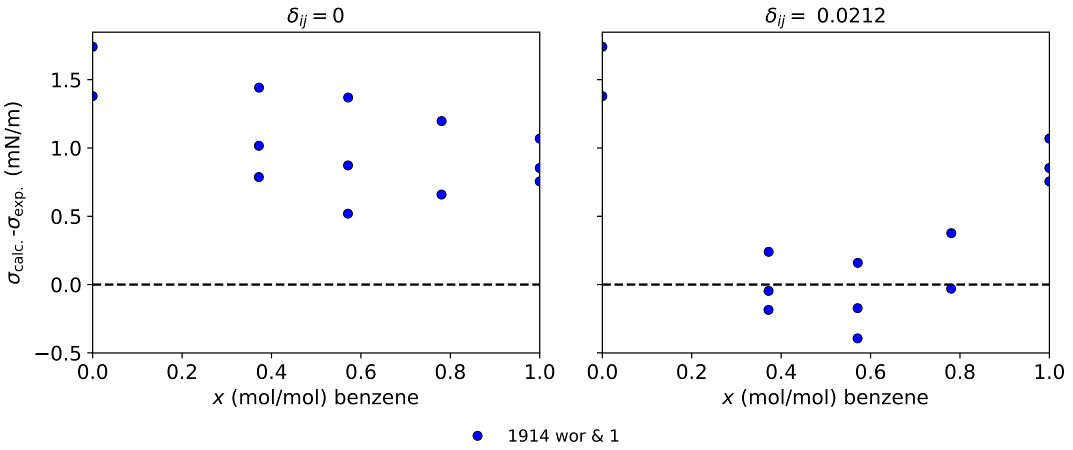

Figure 33: benzene/dichloroethane

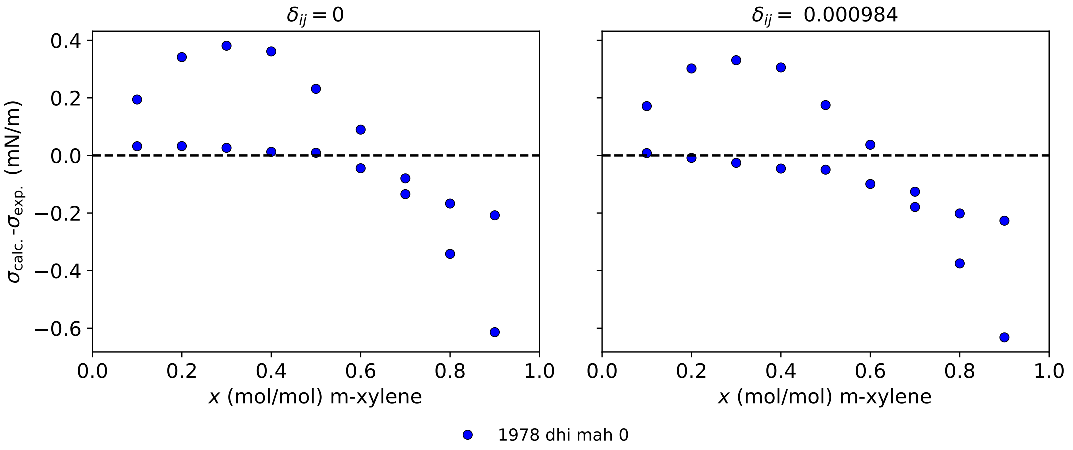

Figure 34: m-xylene/chlorobenzene

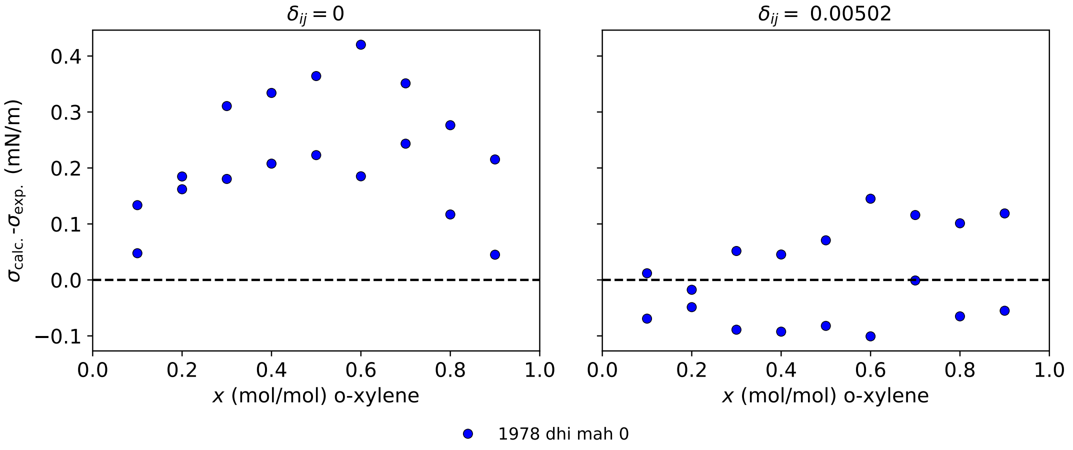

Figure 35: o-xylene/chlorobenzene

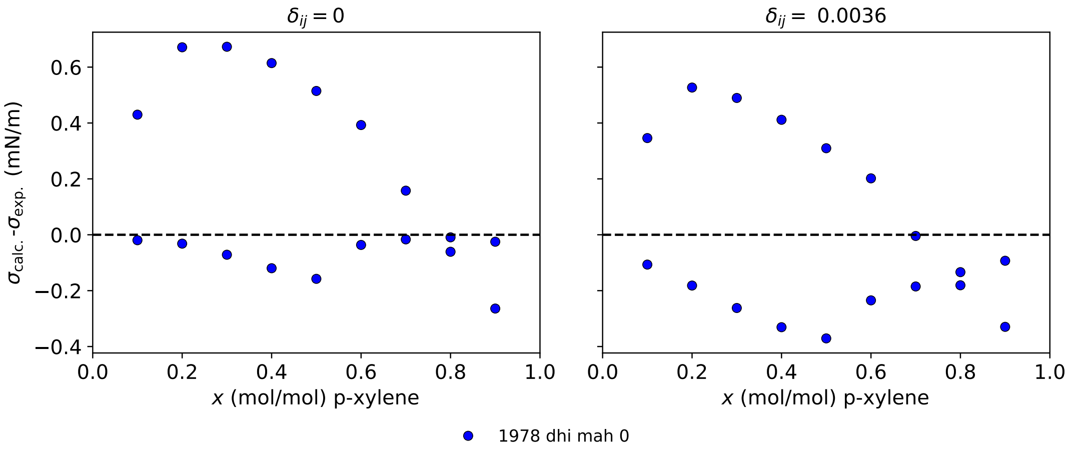

Figure 36: p-xylene/chlorobenzene

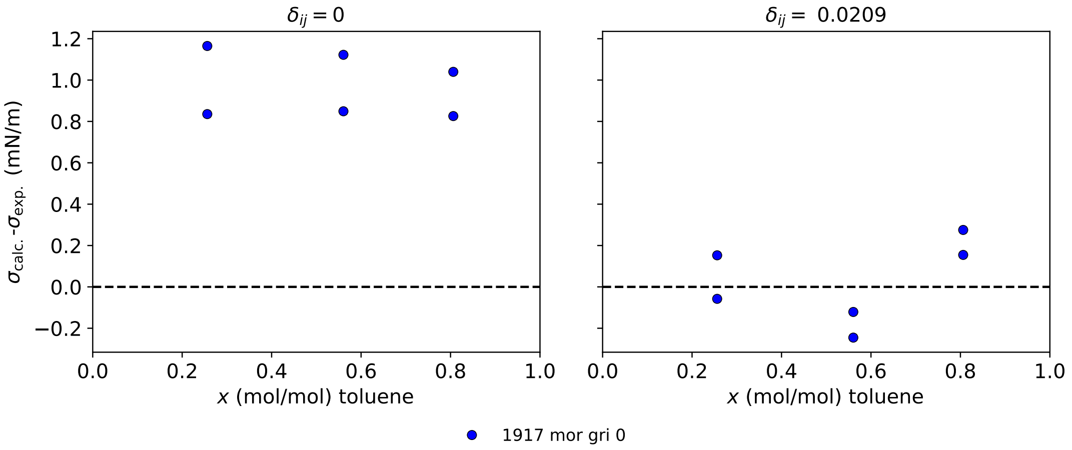

Figure 37: toluene/chlorobenzene

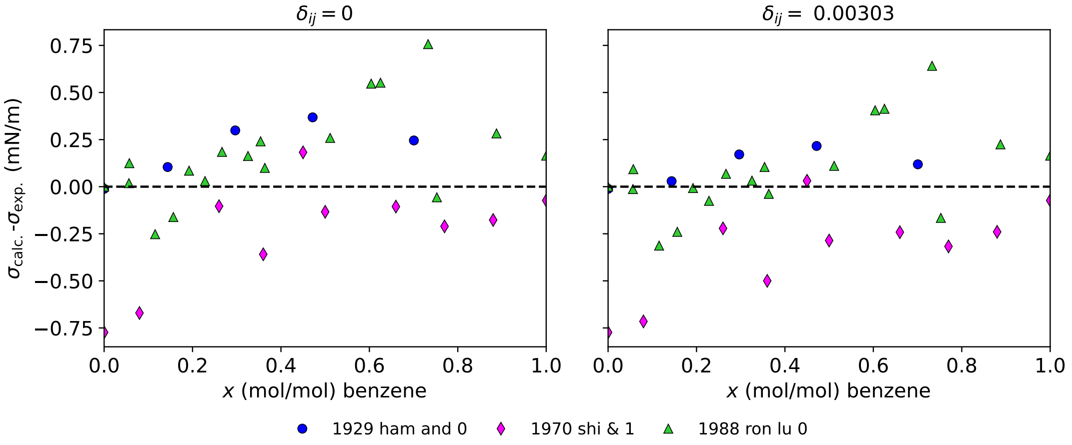

Figure 38: benzene/acetone

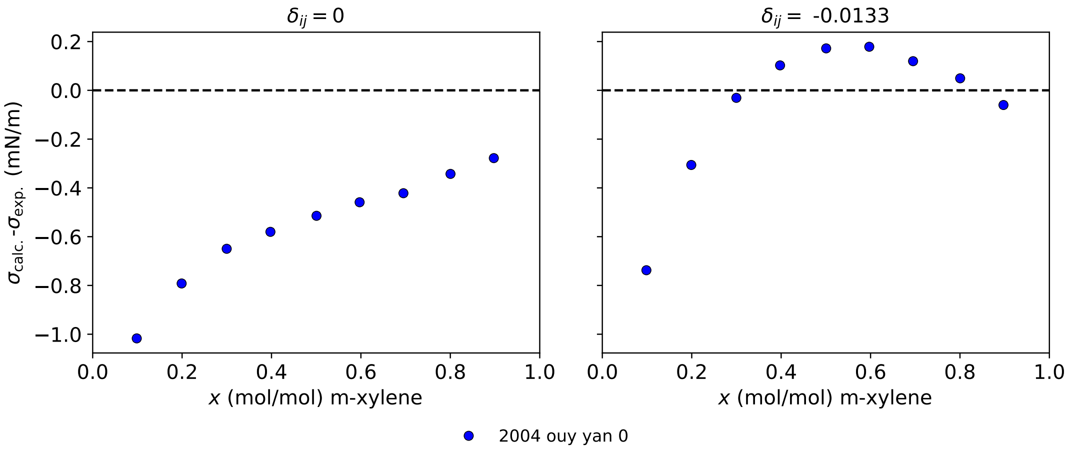

Figure 39: m-xylene/acetone

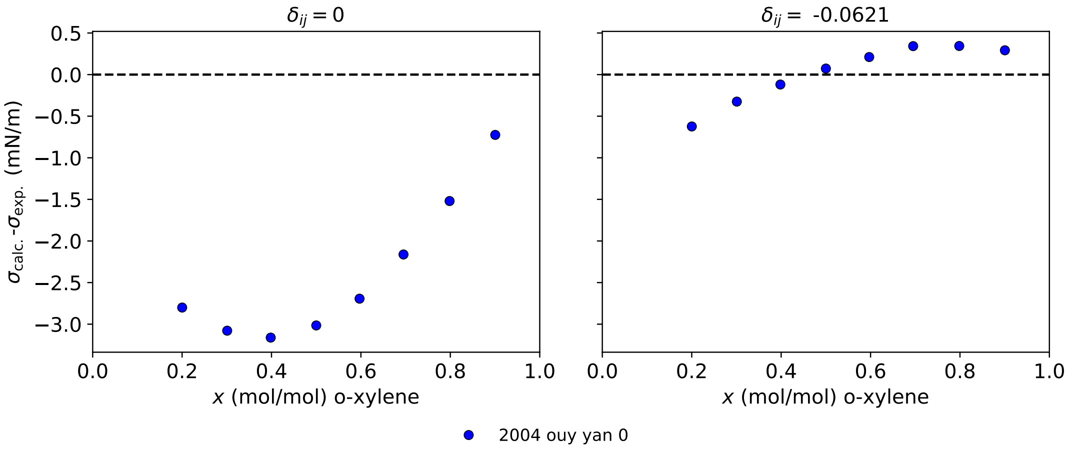

Figure 40: o-xylene/acetone

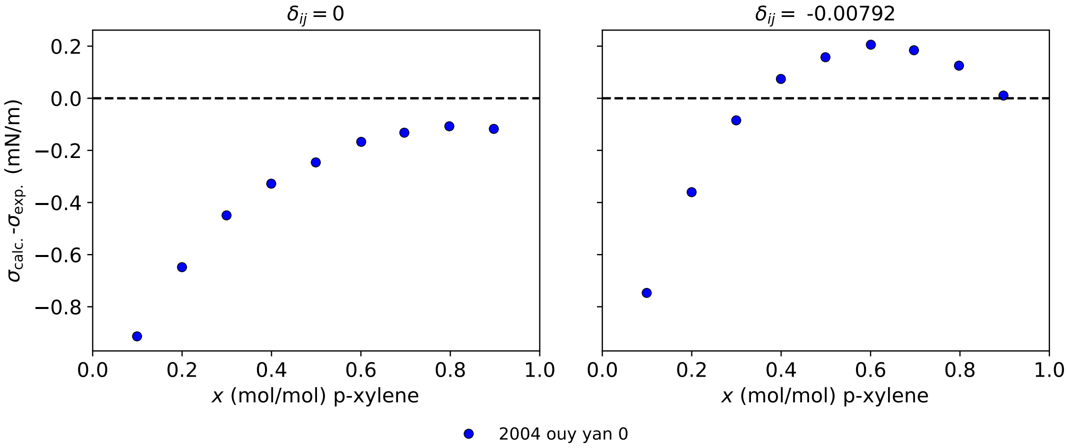

Figure 41: p-xylene/acetone

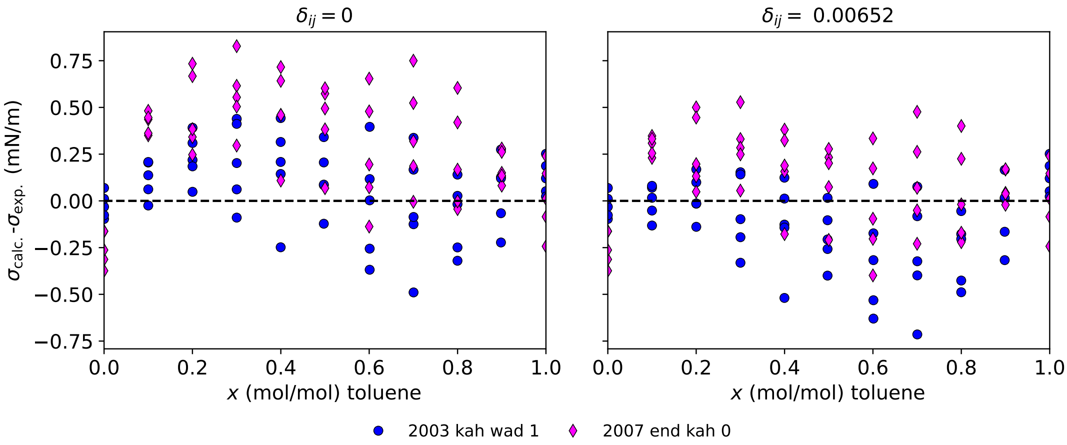

Figure 42: toluene/acetone

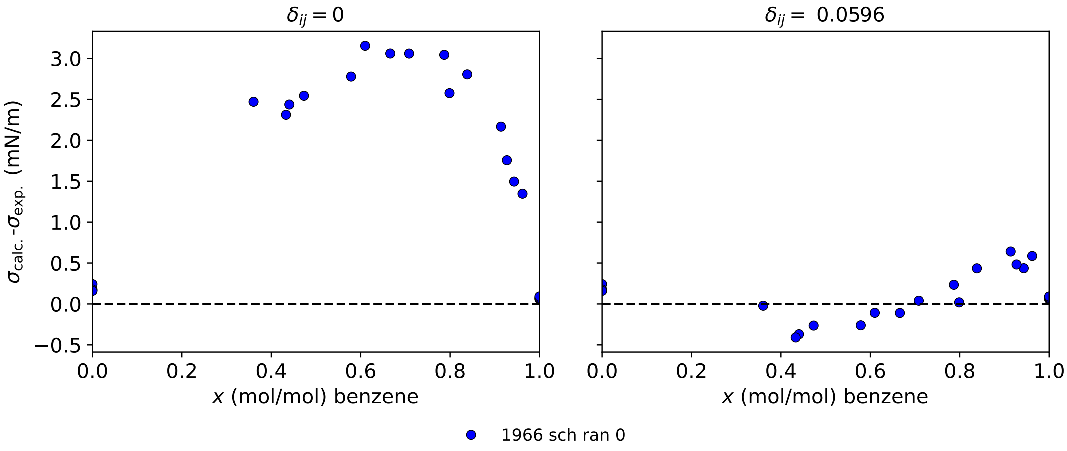

Figure 43: benzene/dodecane

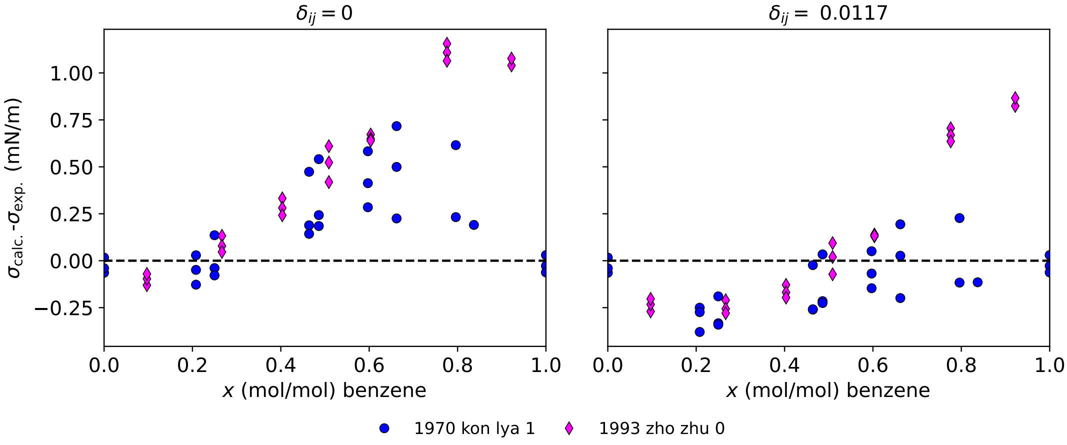

Figure 44: benzene/heptane

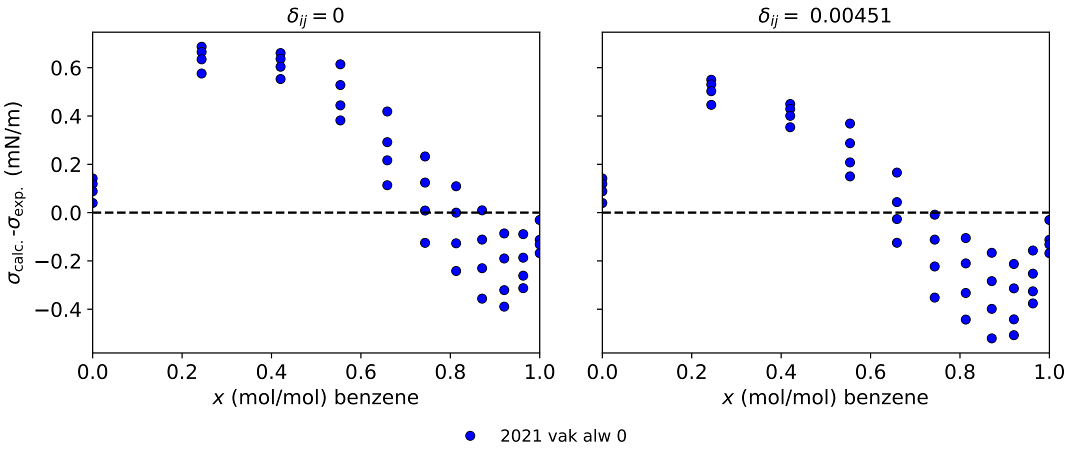

Figure 45: benzene/hexadecane

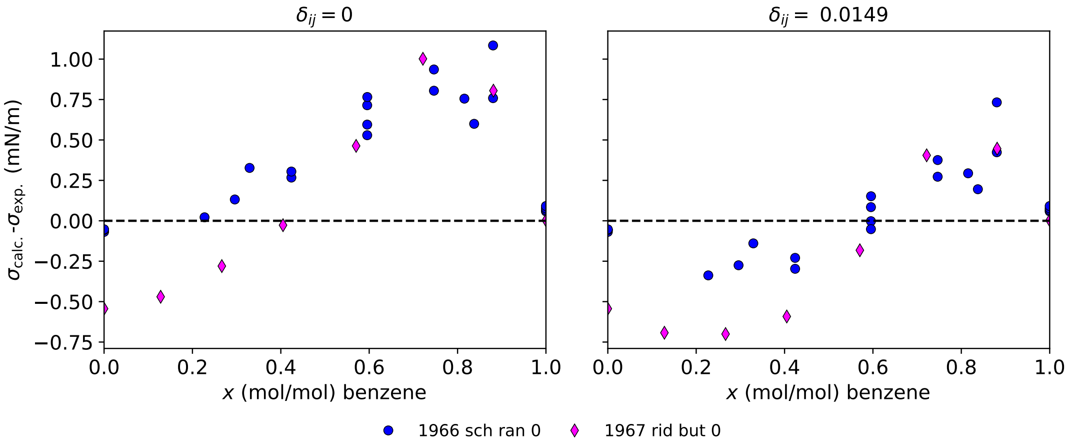

Figure 46: benzene/hexane

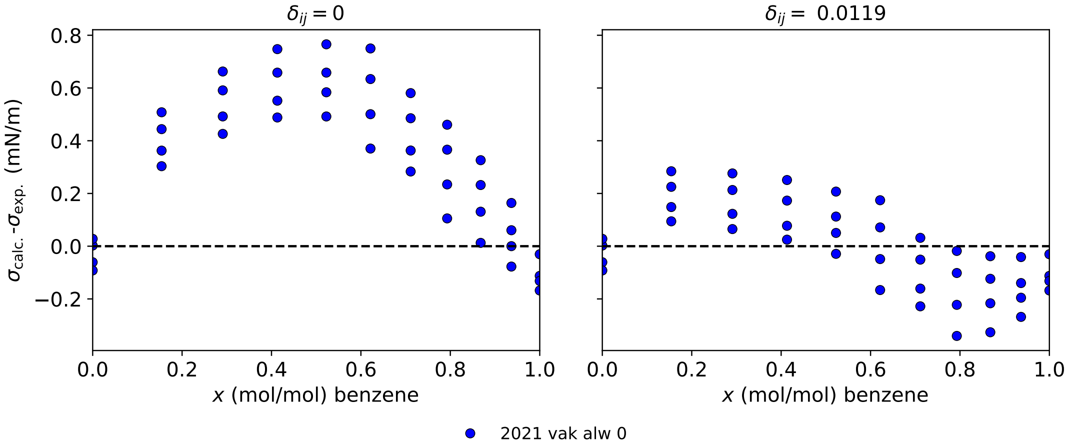

Figure 47: benzene/nonane

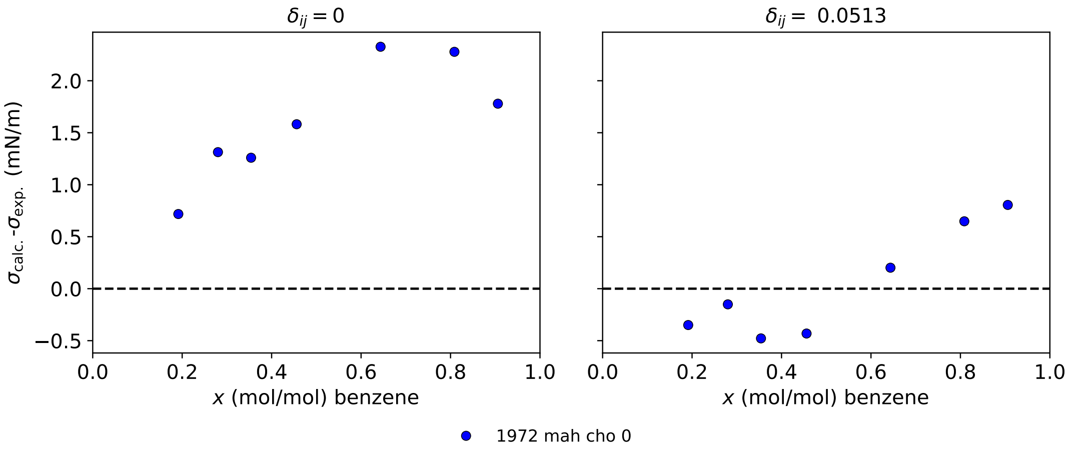

Figure 48: benzene/pentane

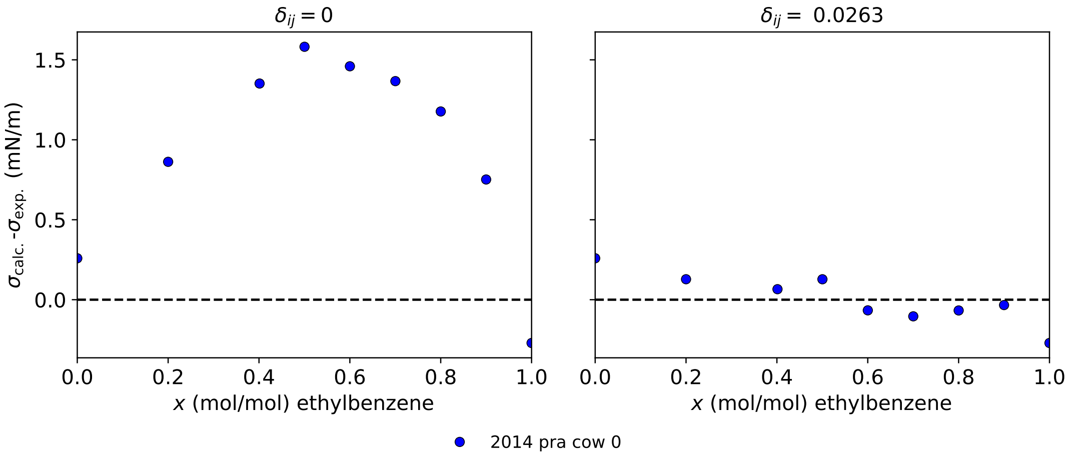

Figure 49: ethylbenzene/hexadecane

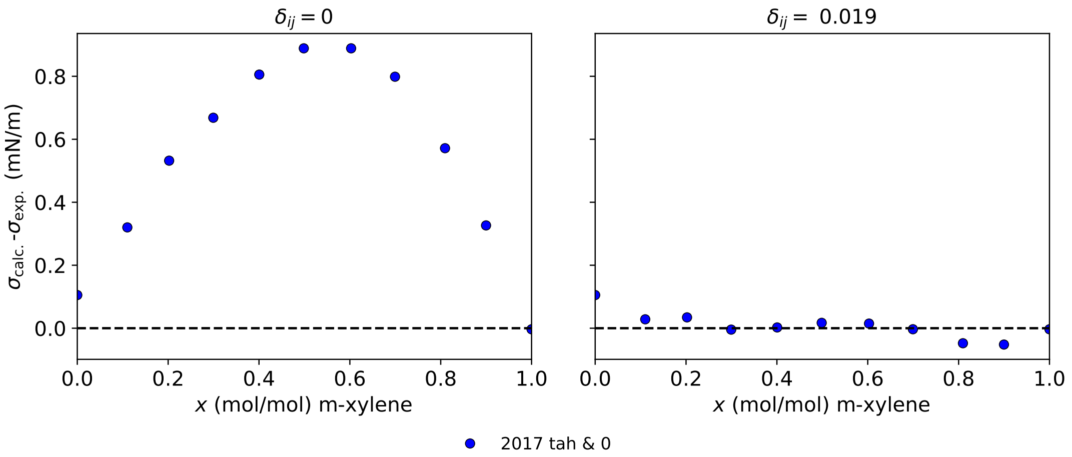

Figure 50: m-xylene/heptane

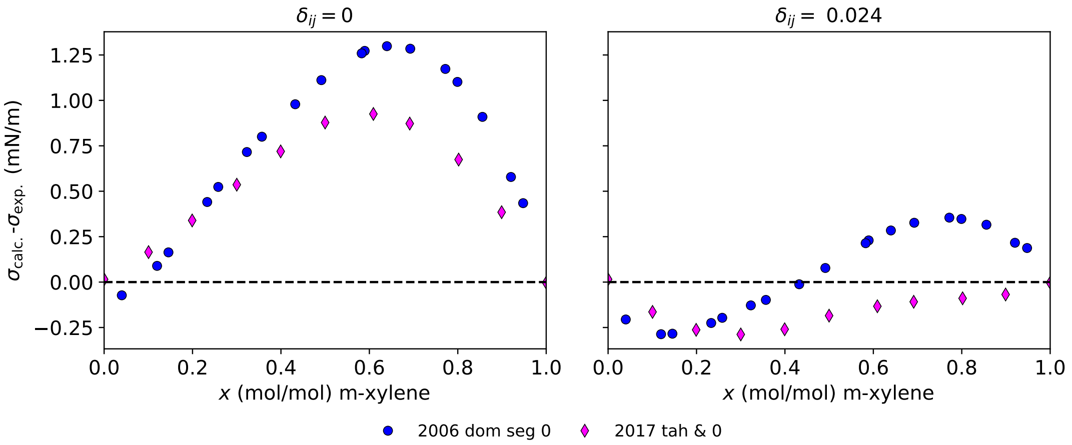

Figure 51: m-xylene/hexane

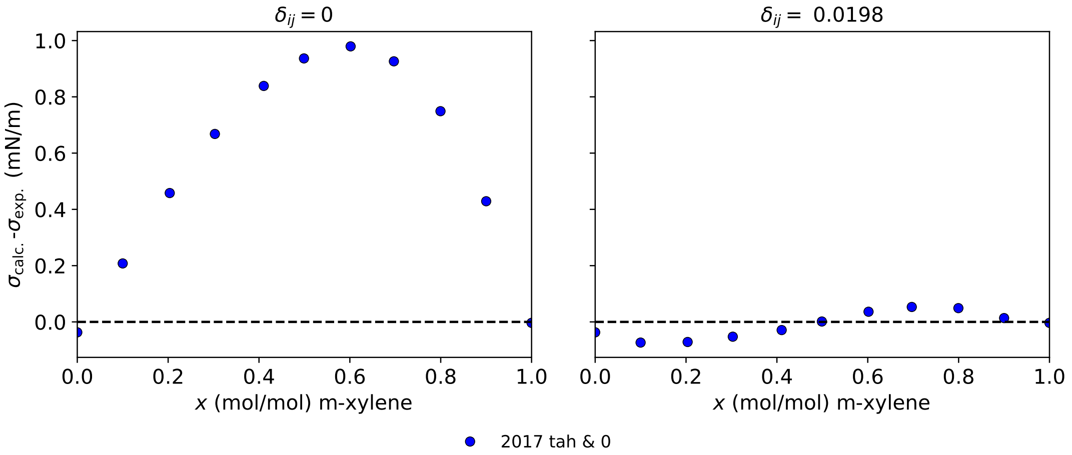

Figure 52: m-xylene/octane

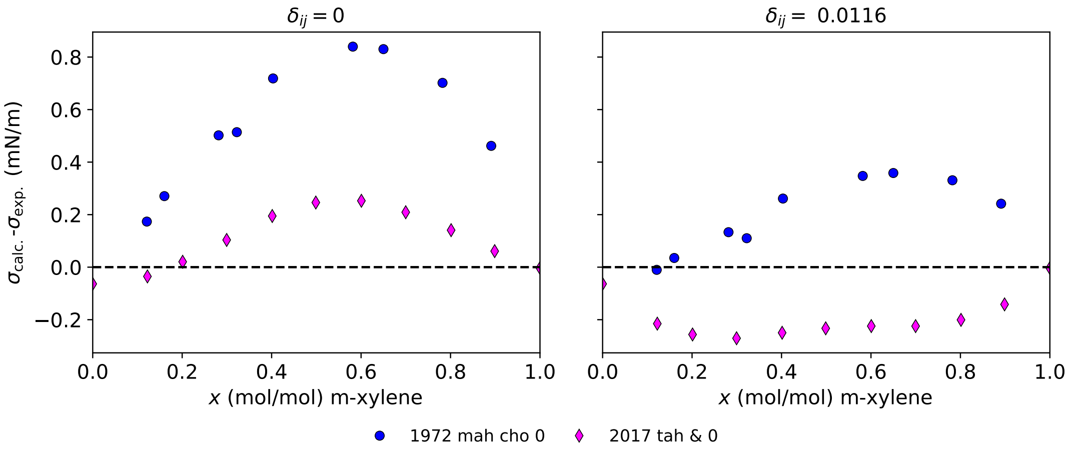

Figure 53: m-xylene/pentane

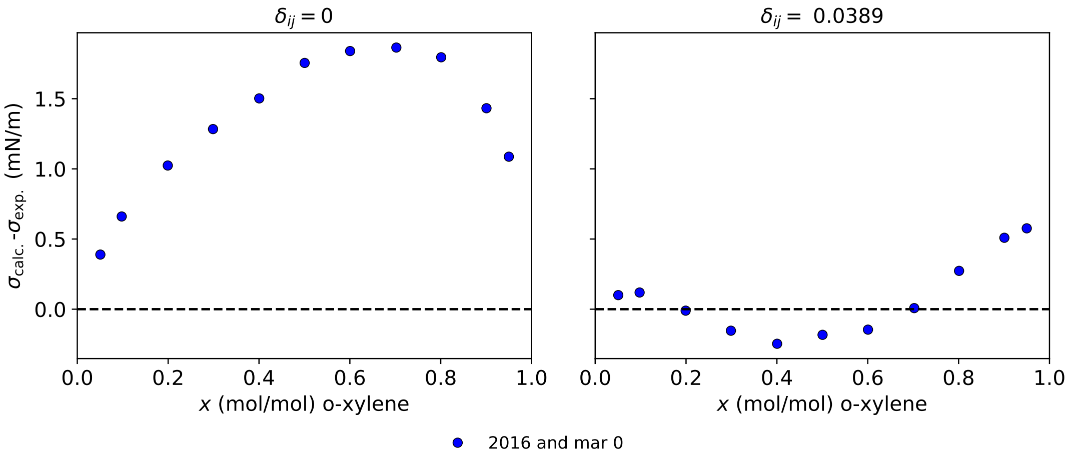

Figure 54: o-xylene/decane

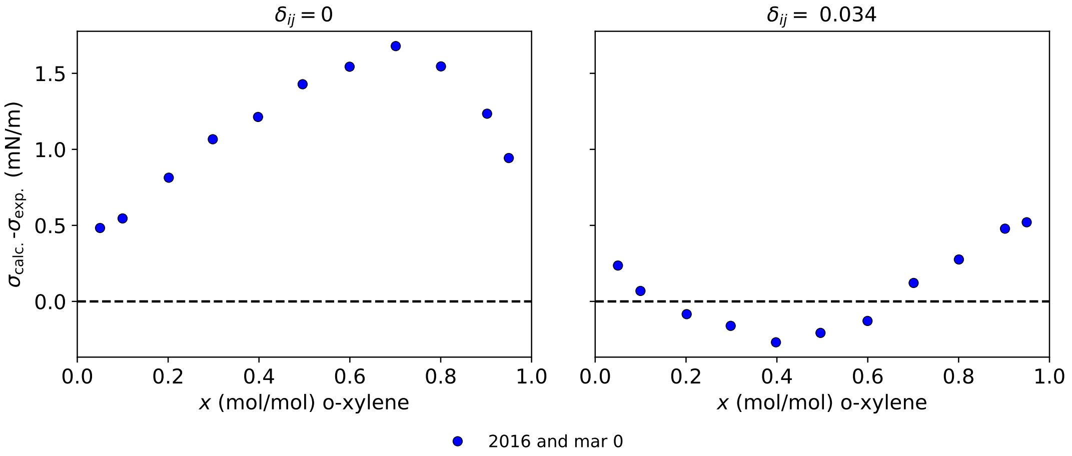

Figure 55: o-xylene/nonane

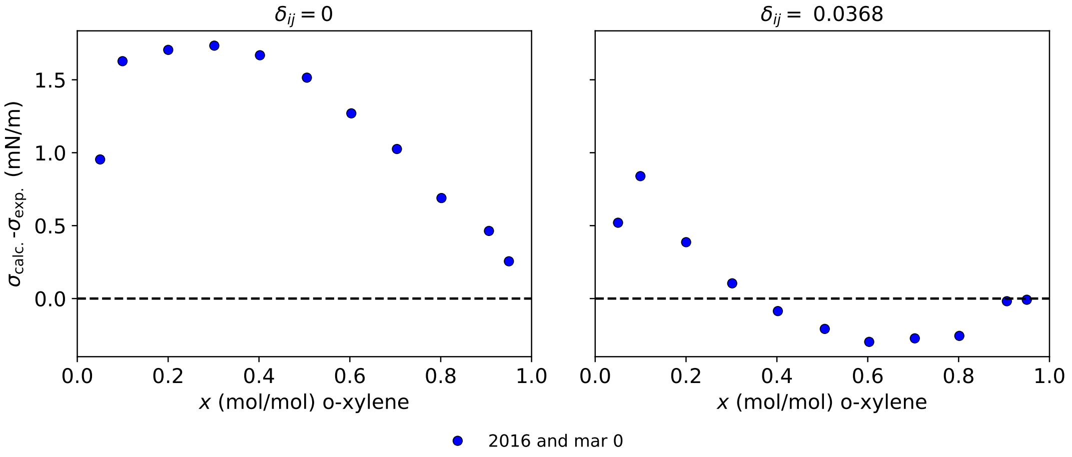

Figure 56: o-xylene/octane

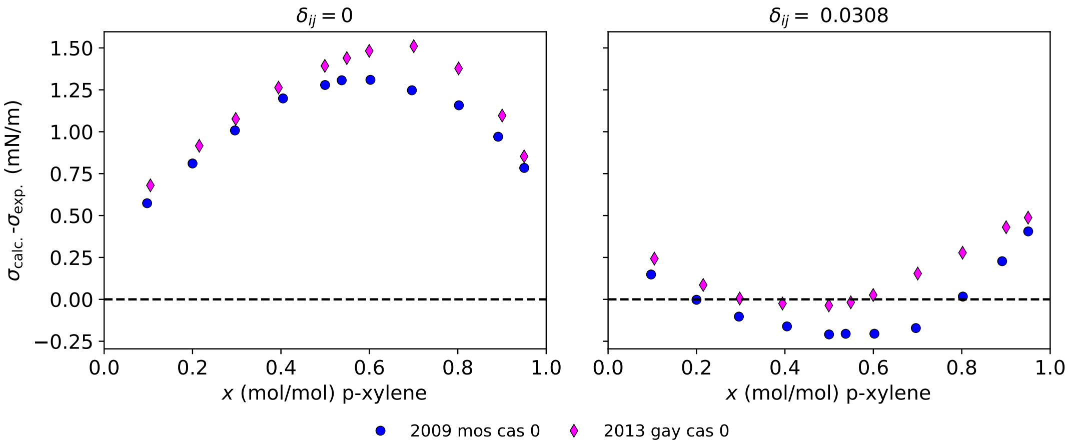

Figure 57: p-xylene/decane

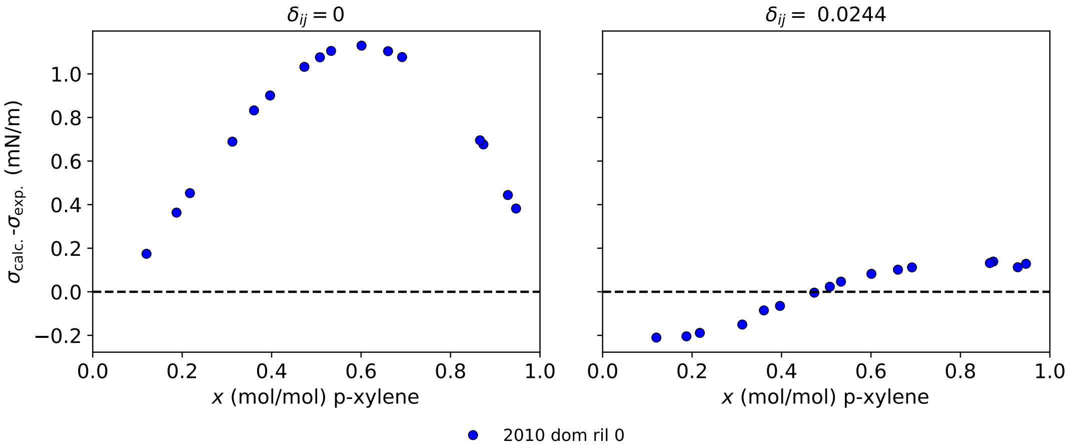

Figure 58: p-xylene/hexane

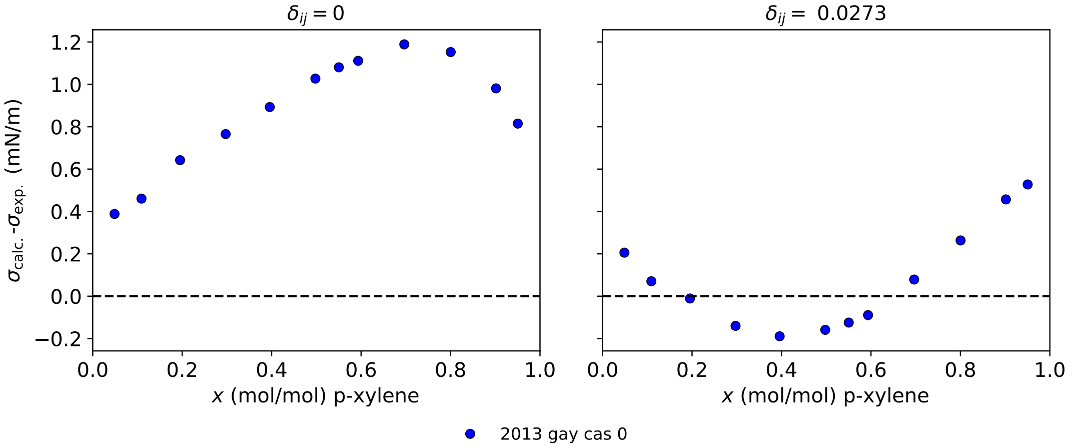

Figure 59: p-xylene/octane

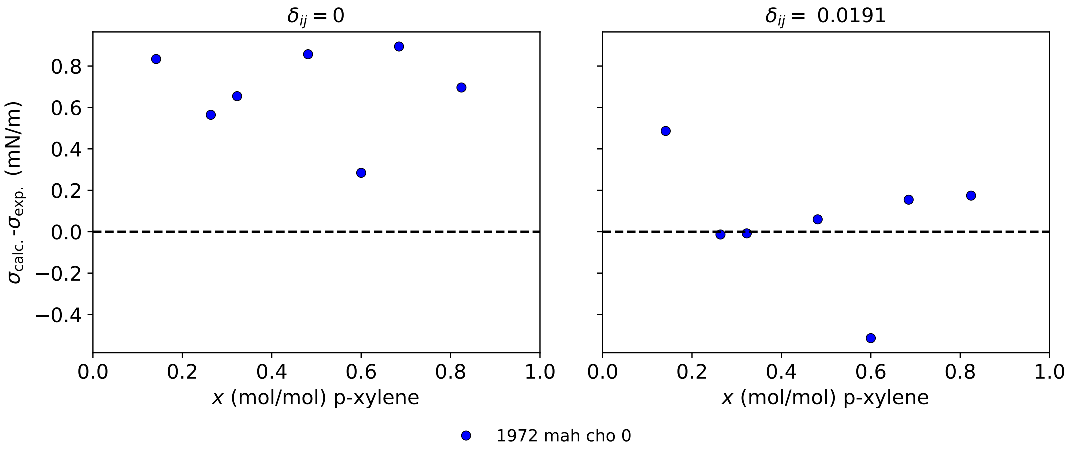

Figure 60: p-xylene/pentane

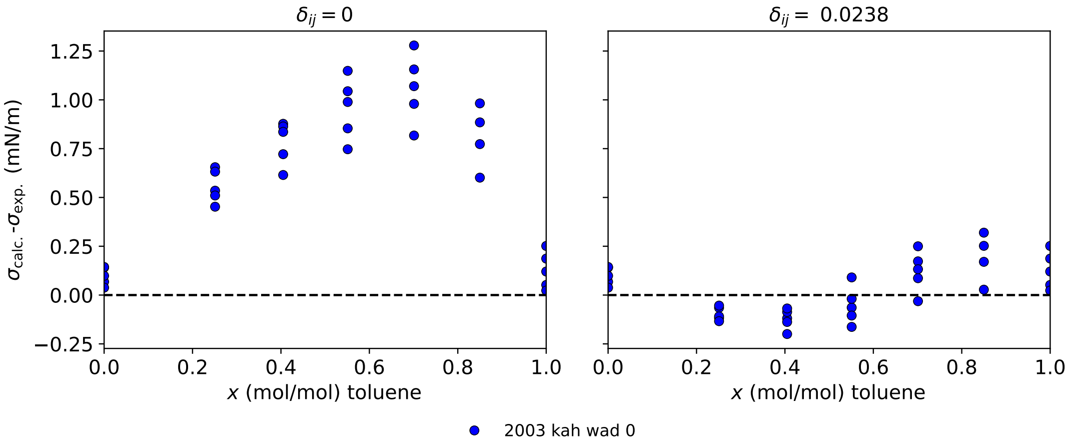

Figure 61: toluene/heptane

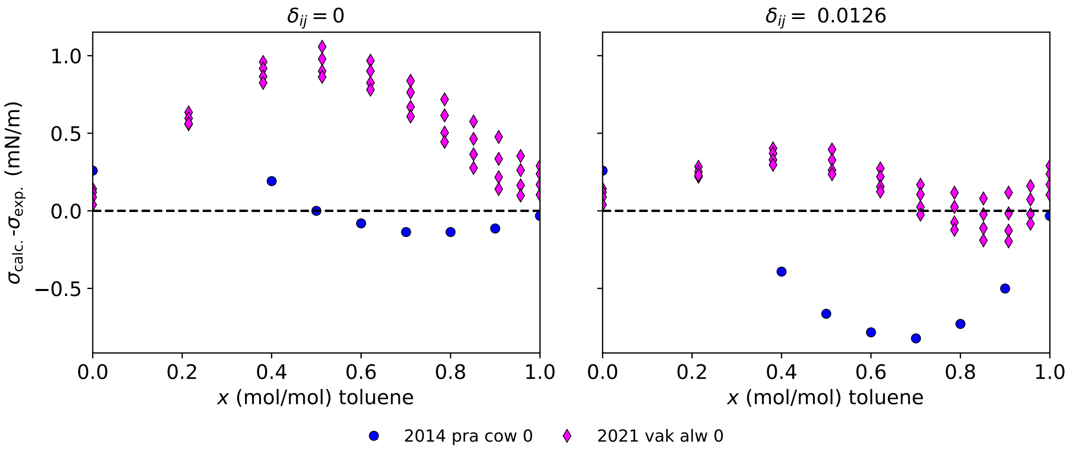

Figure 62: toluene/hexadecane

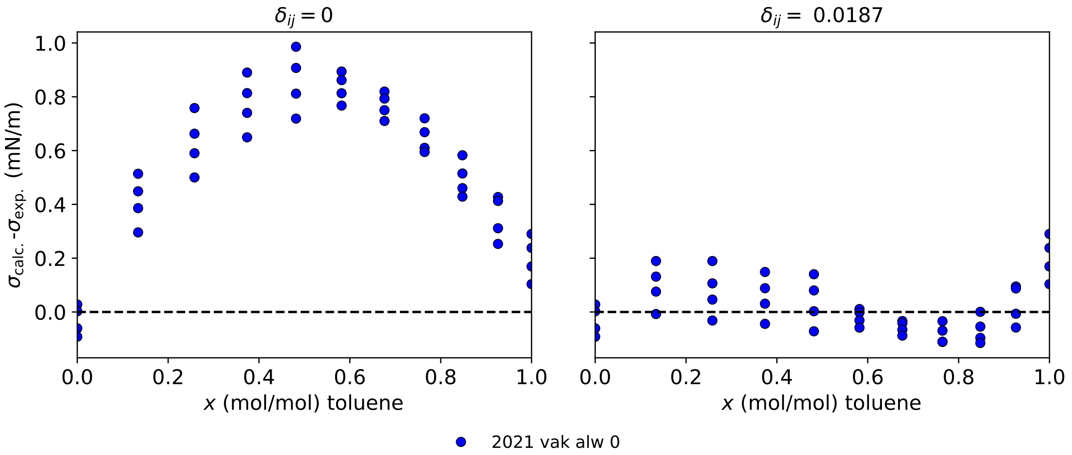

Figure 63: toluene/nonane

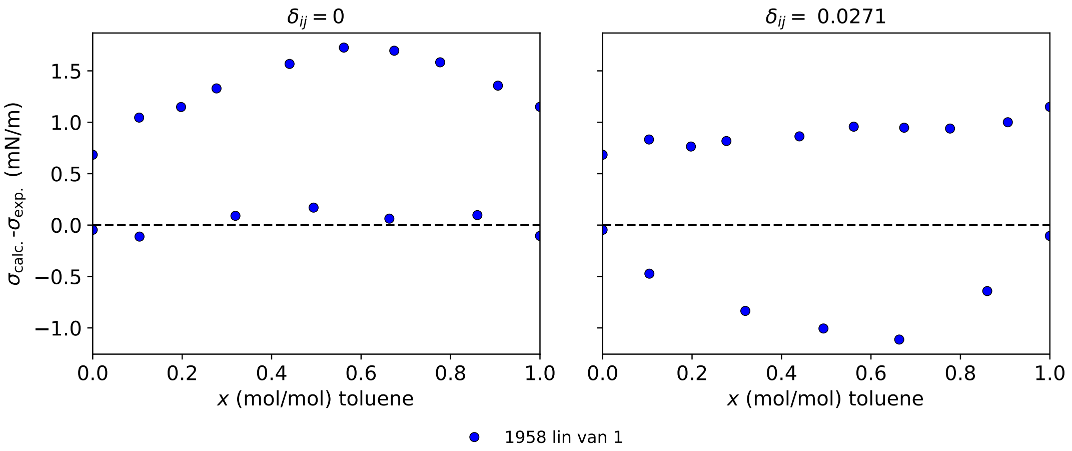

Figure 64: toluene/octane

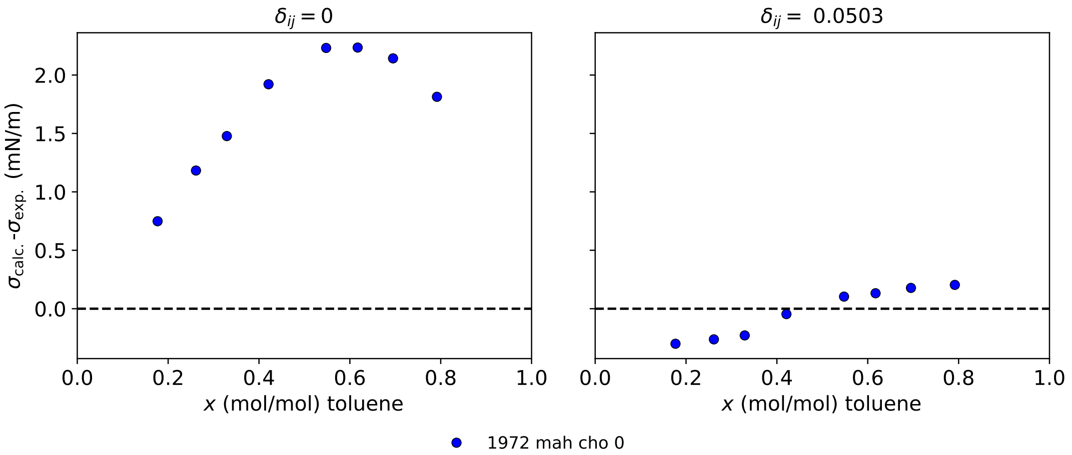

Figure 65: toluene/pentane

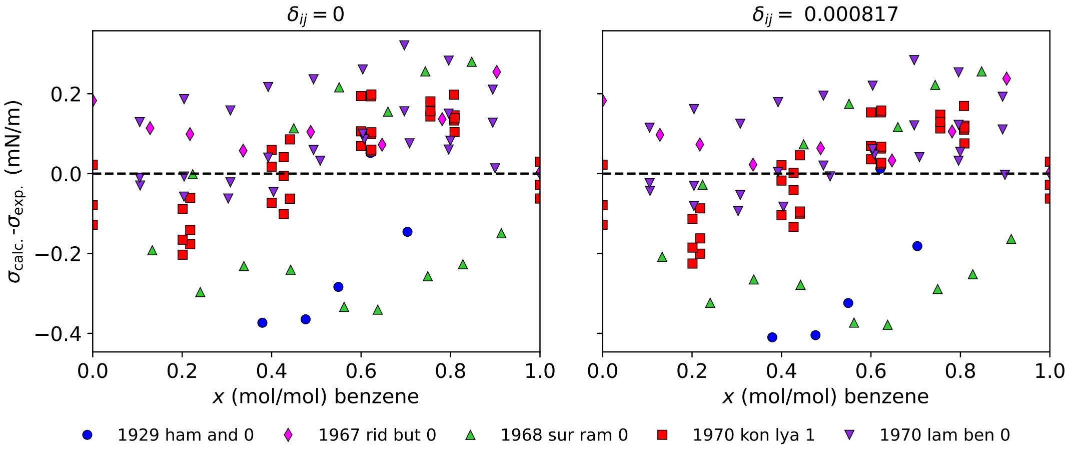

Figure 66: benzene/cyclohexane

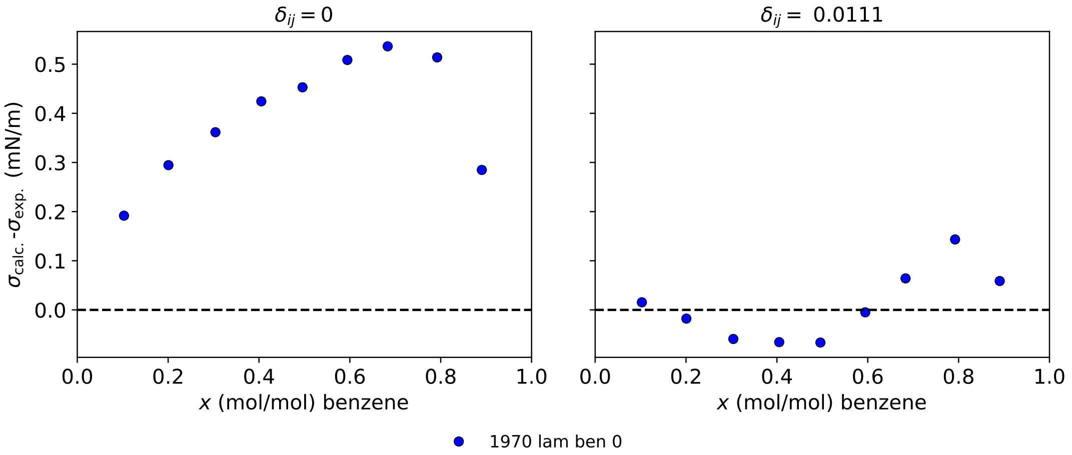

Figure 67: benzene/cyclopentane

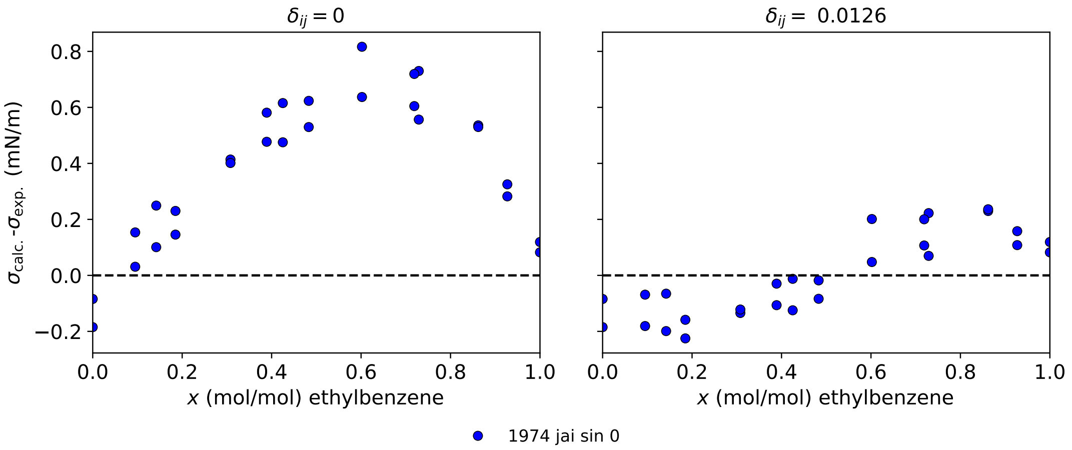

Figure 68: ethylbenzene/cyclohexane

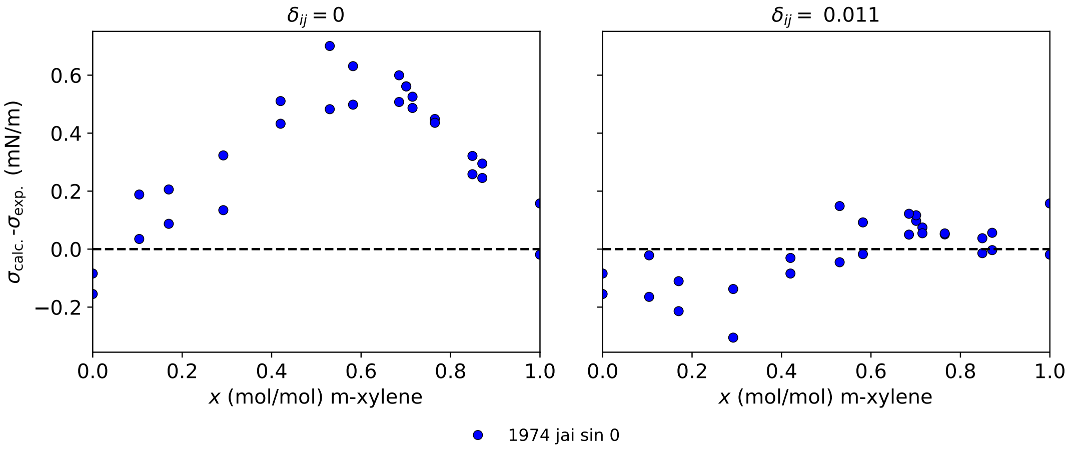

Figure 69: m-xylene/cyclohexane

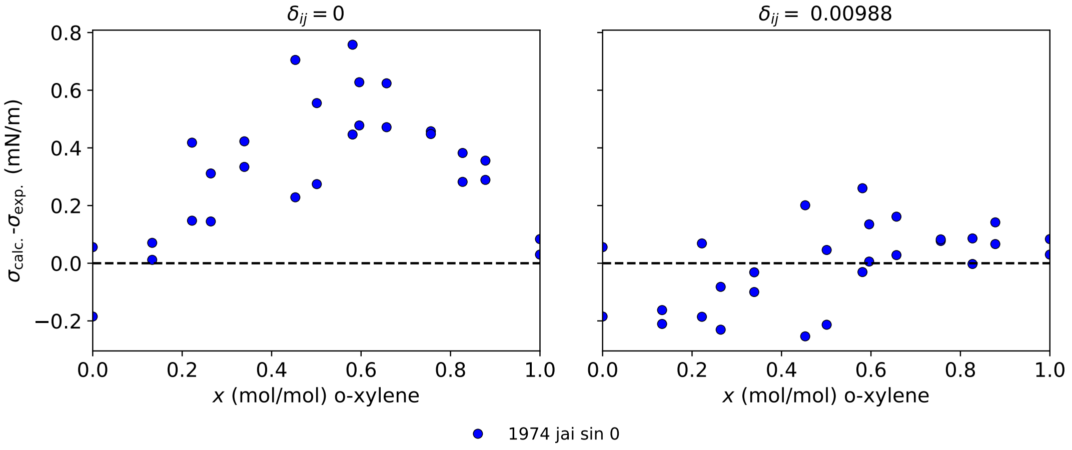

Figure 70: o-xylene/cyclohexane

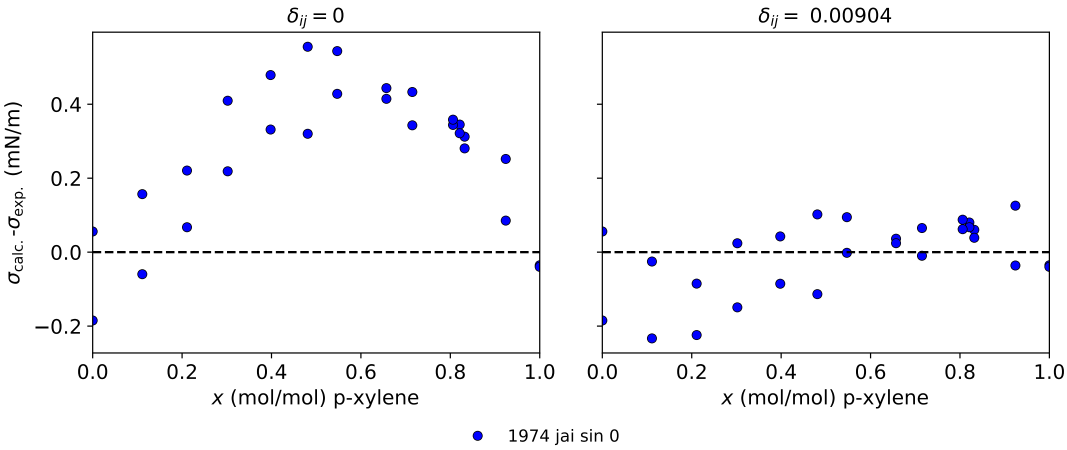

Figure 71: p-xylene/cyclohexane

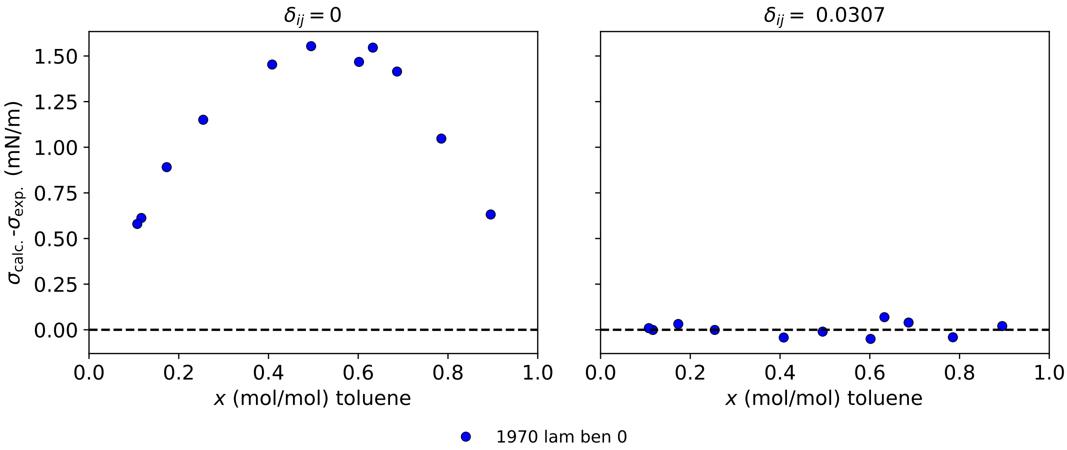

Figure 72: toluene/cyclohexane

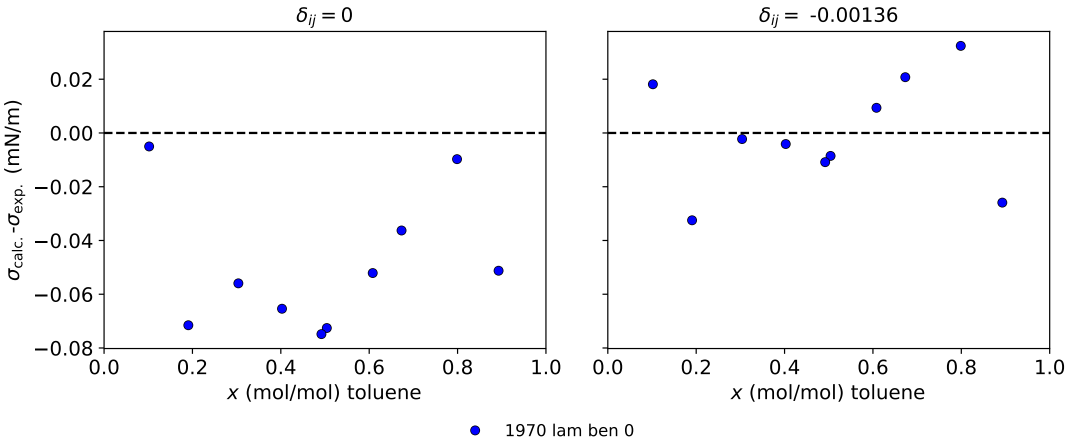

Figure 73: toluene/cyclopentane

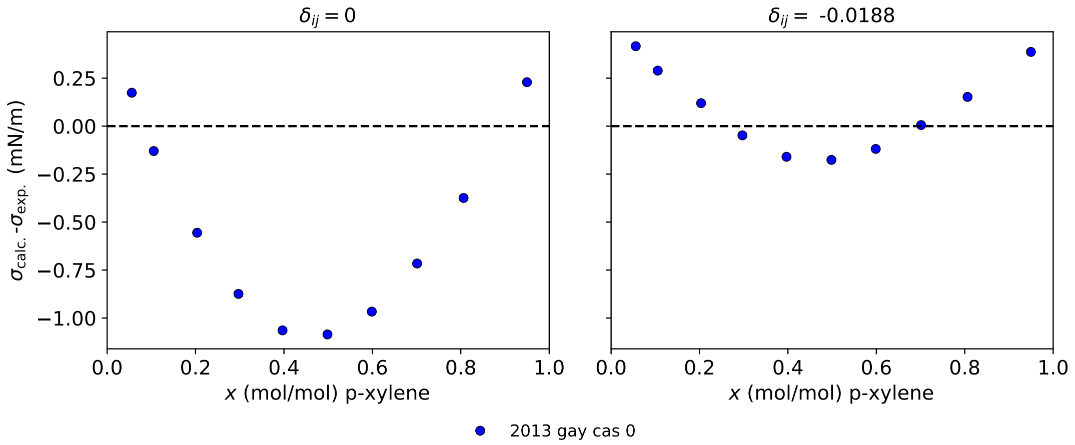

Figure 74: p-xylene/dimethyl carbonate

3 Halocarbon mixtures

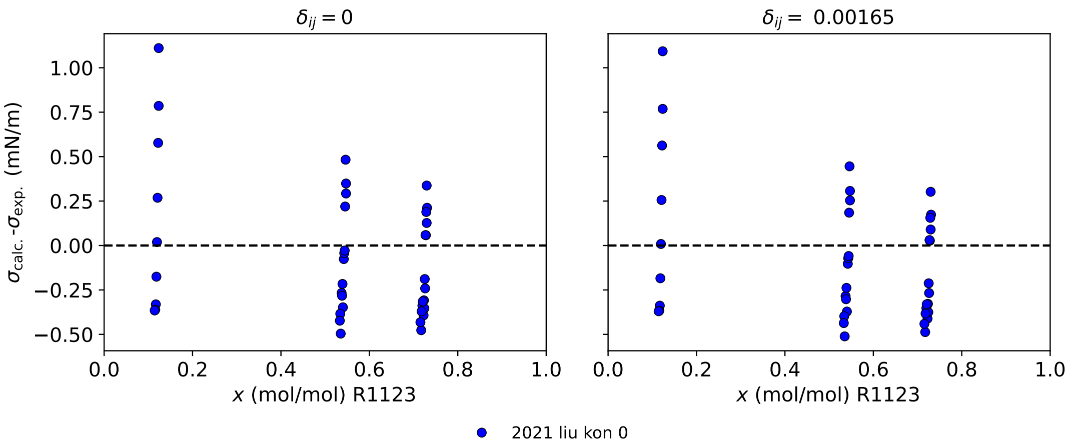

Figure 75: R1123/R1234yf

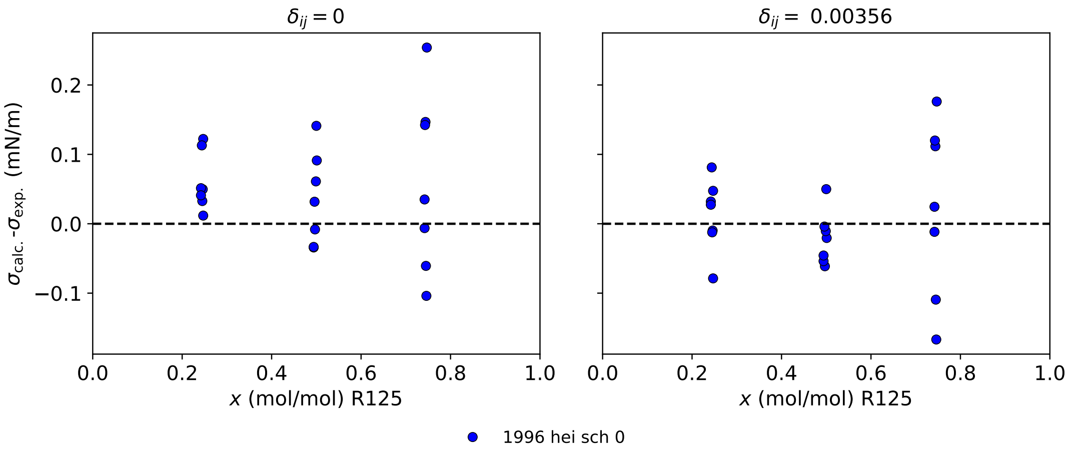

Figure 76: R125/R134a

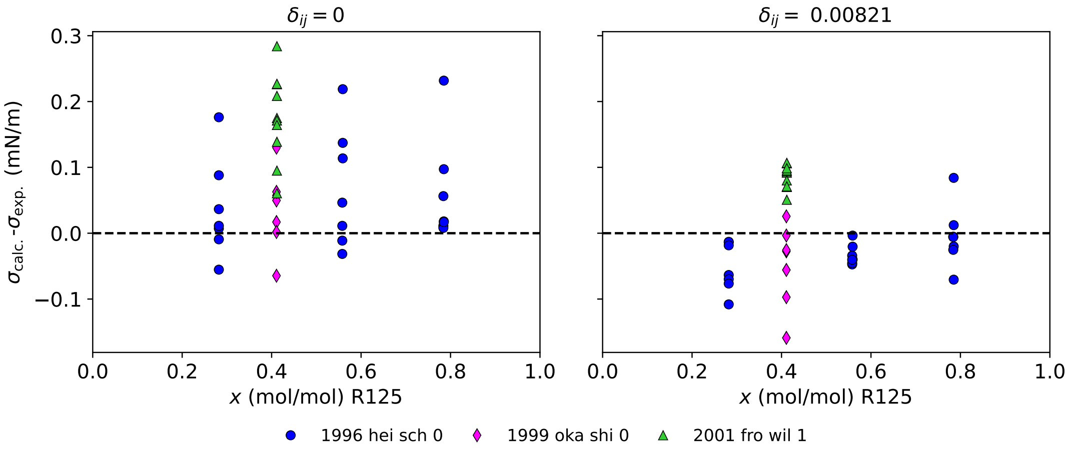

Figure 77: R125/R143a

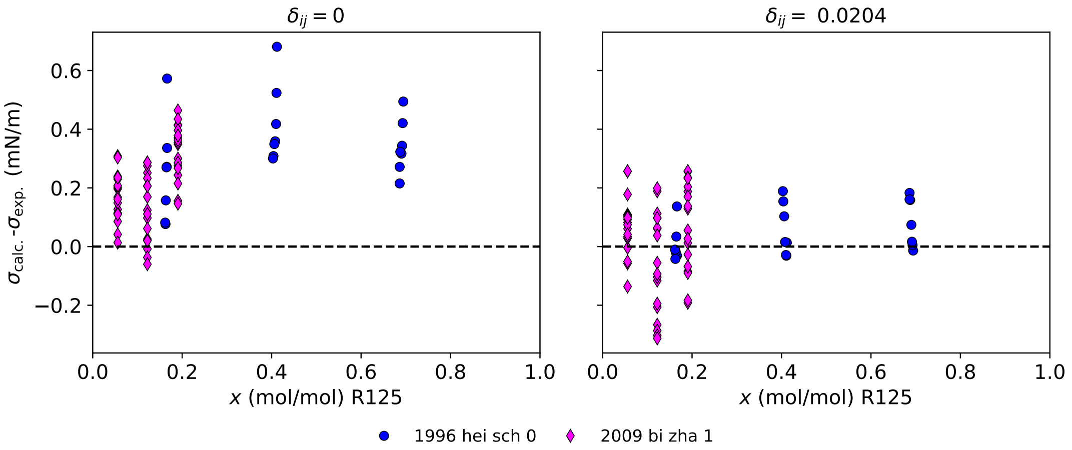

Figure 78: R125/R152a

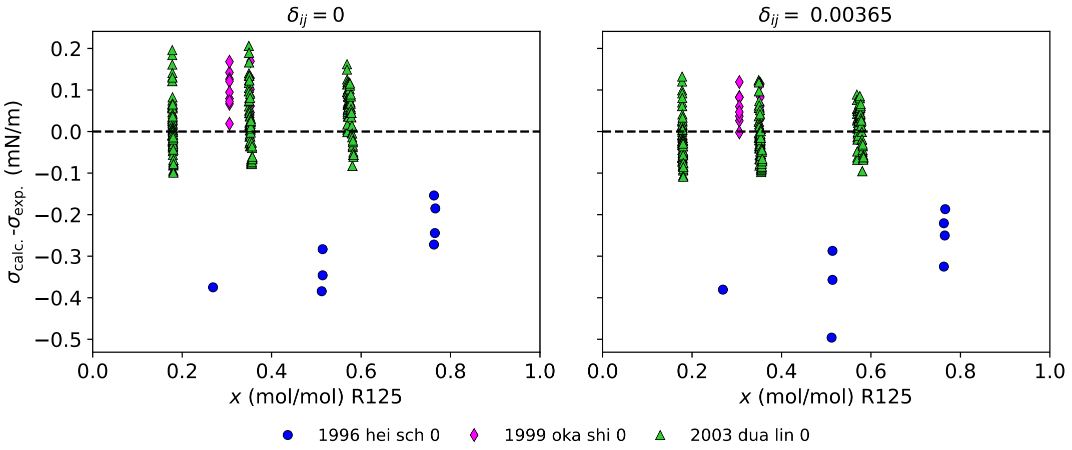

Figure 79: R125/R32

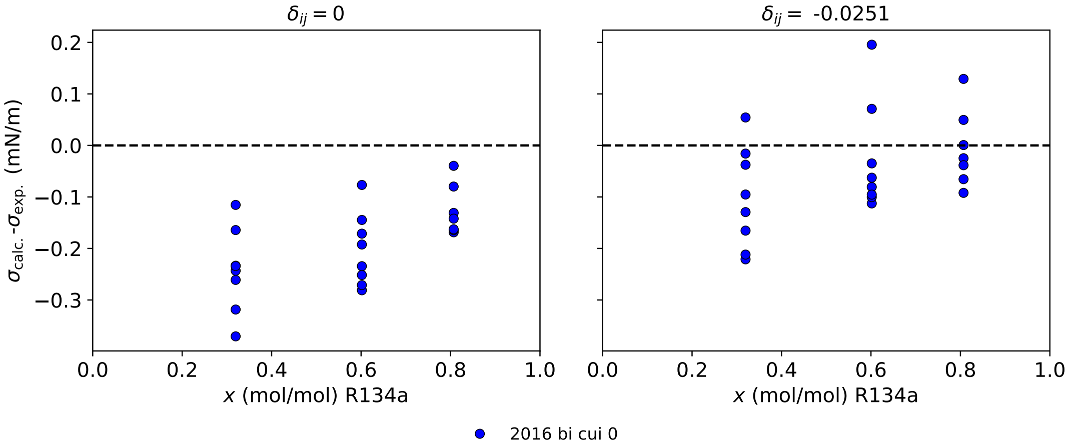

Figure 80: R134a/R1234yf

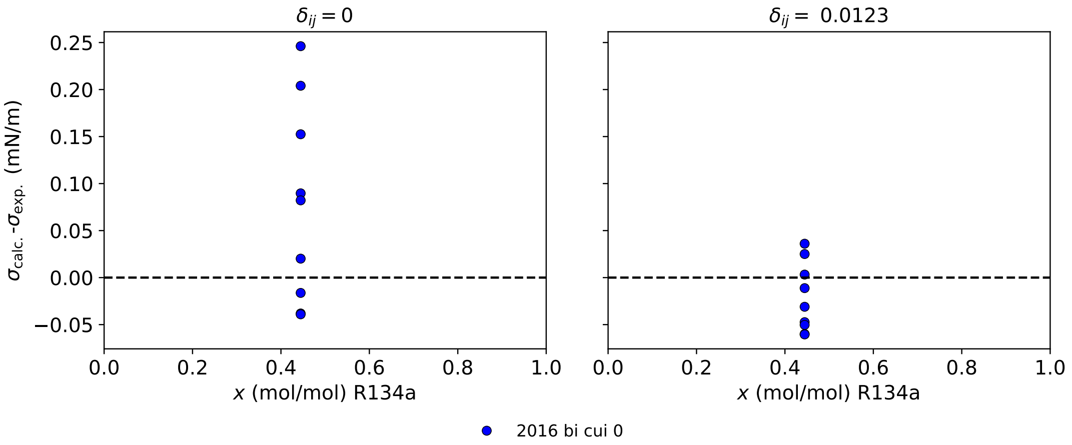

Figure 81: R134a/R1234ze(e)

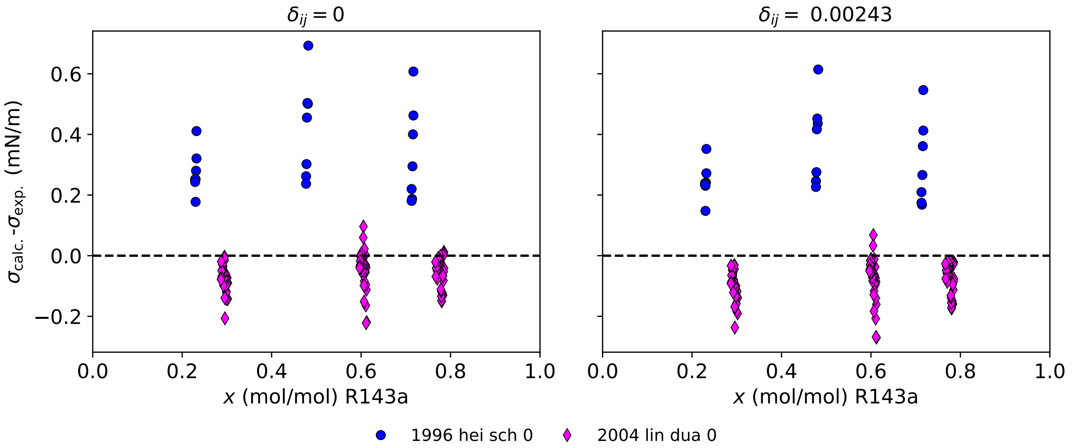

Figure 82: R143a/R134a

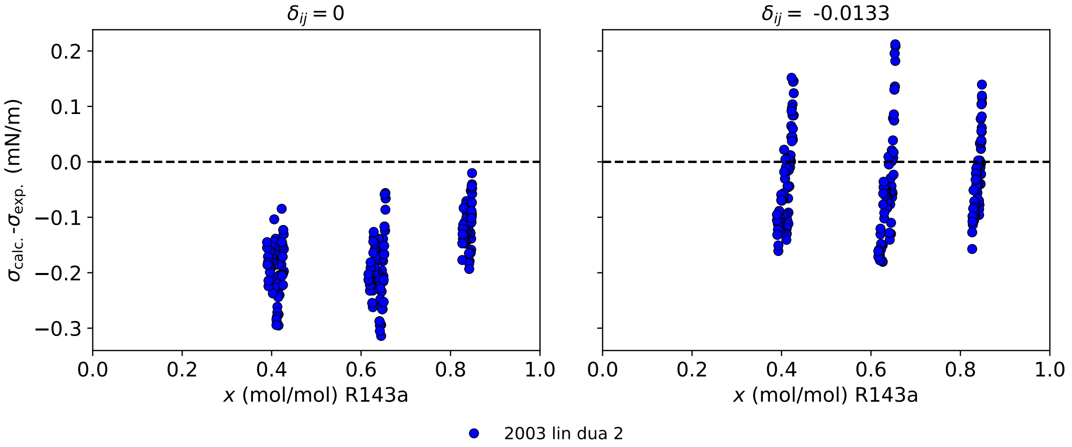

Figure 83: R143a/R227ea

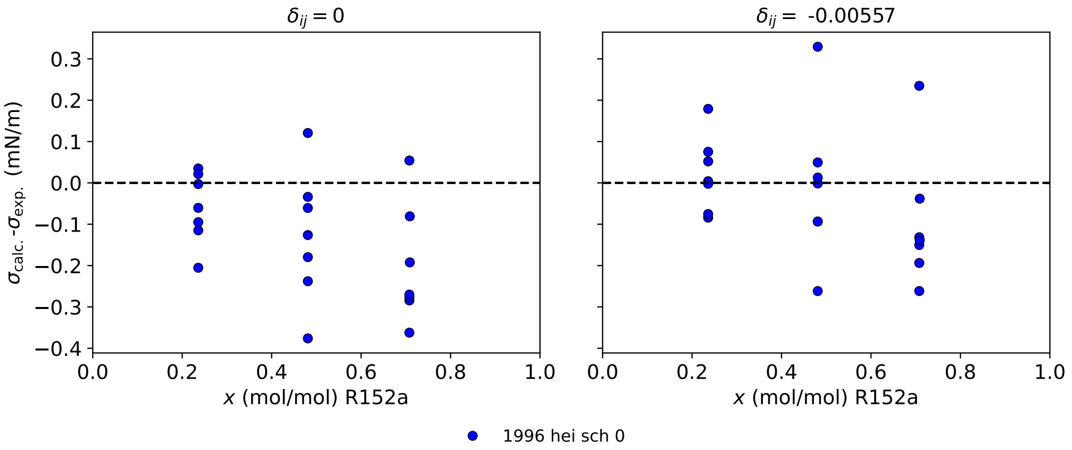

Figure 84: R152a/R134a

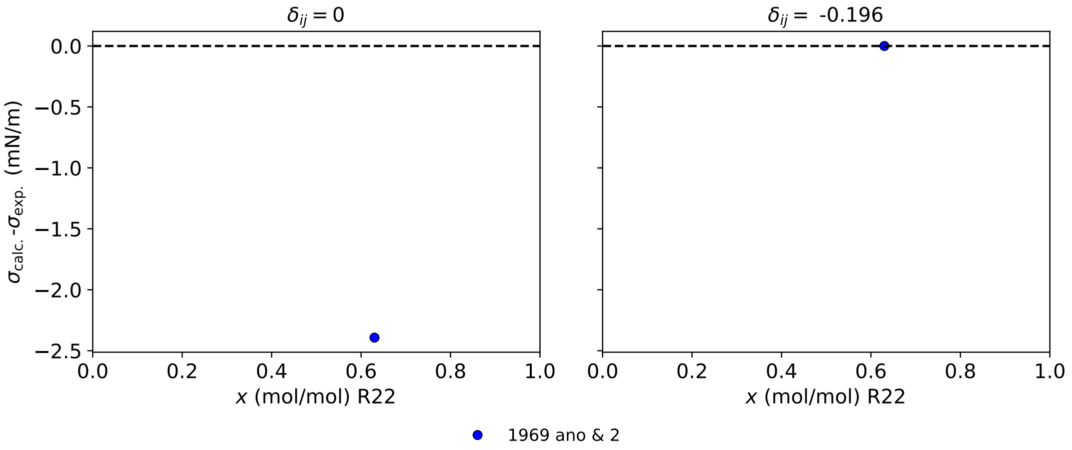

Figure 85: R22/R115

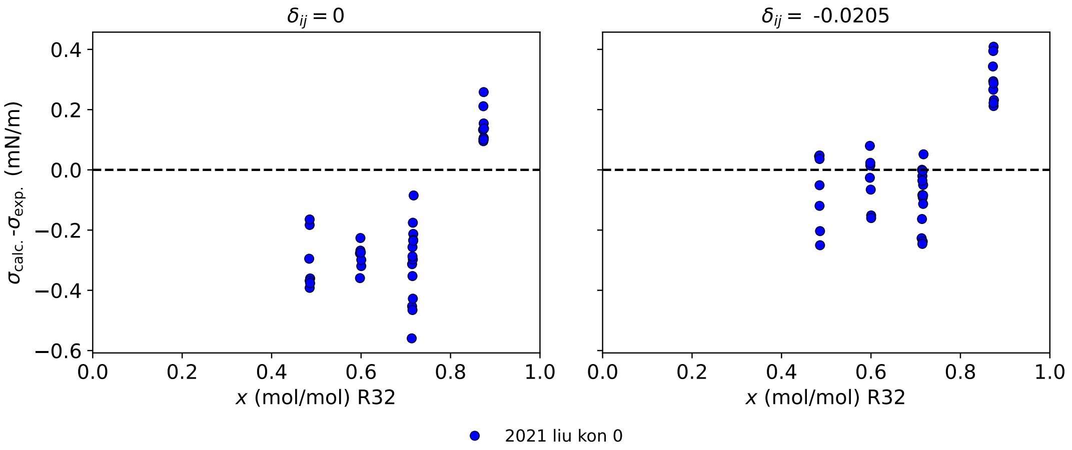

Figure 86: R32/R1123

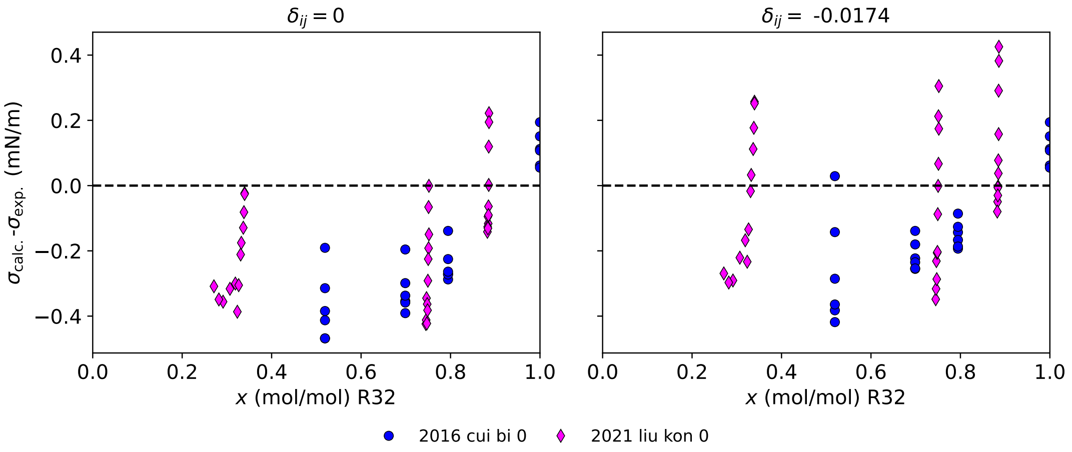

Figure 87: R32/R1234yf

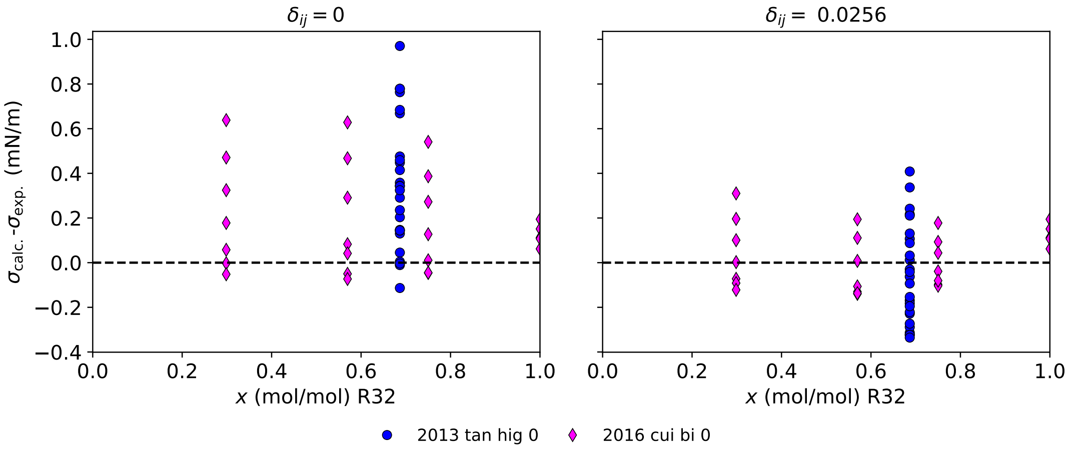

Figure 88: R32/R1234ze(e)

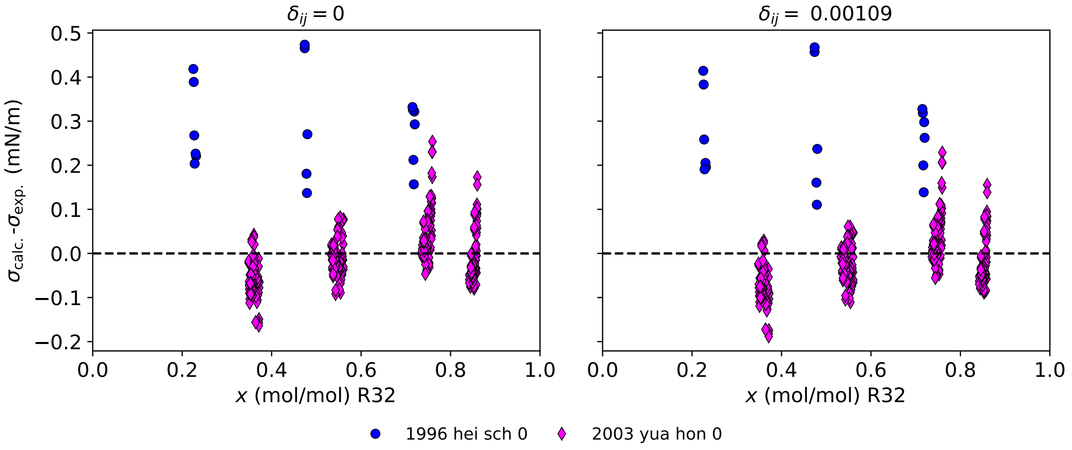

Figure 89: R32/R134a

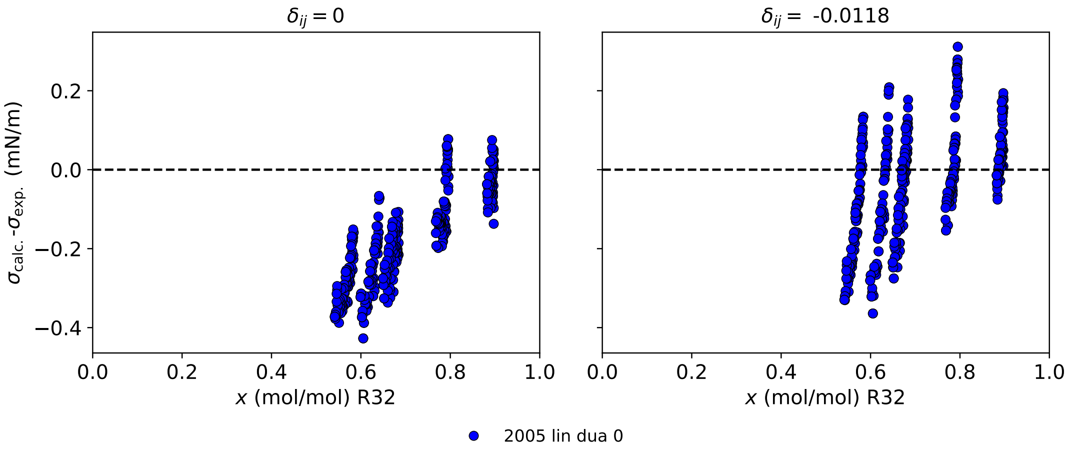

Figure 90: R32/R227ea

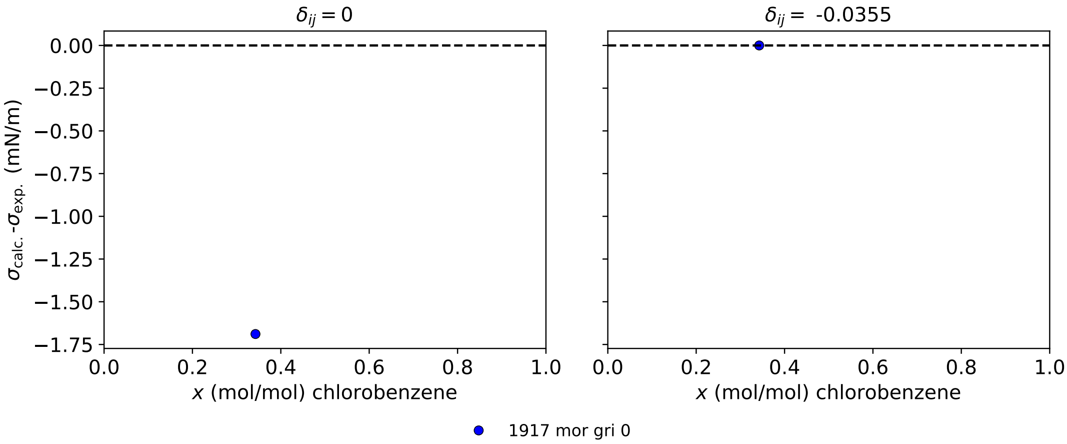

Figure 91: chlorobenzene/acetone

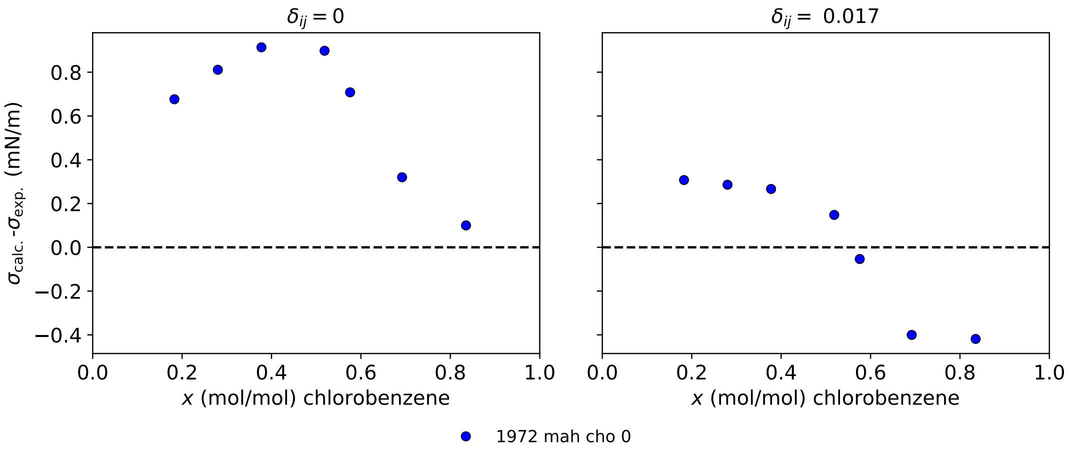

Figure 92: chlorobenzene/pentane

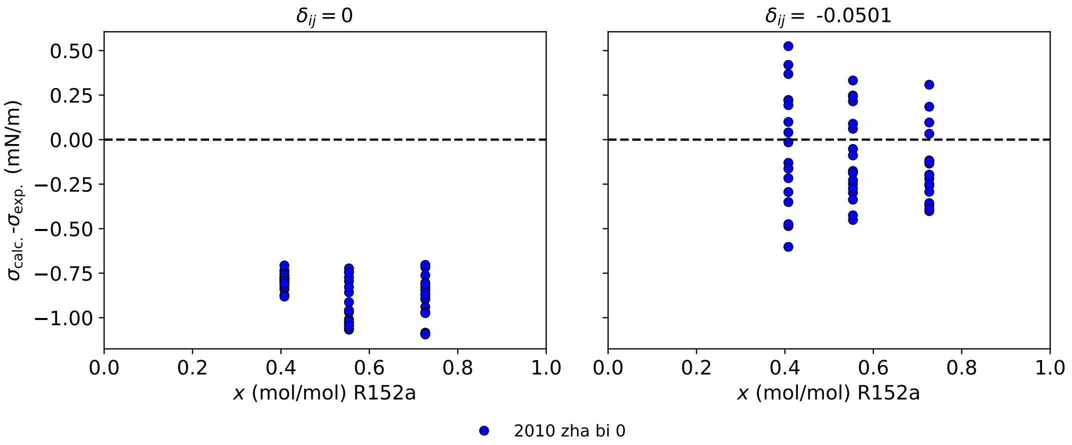

Figure 93: R152a/propane

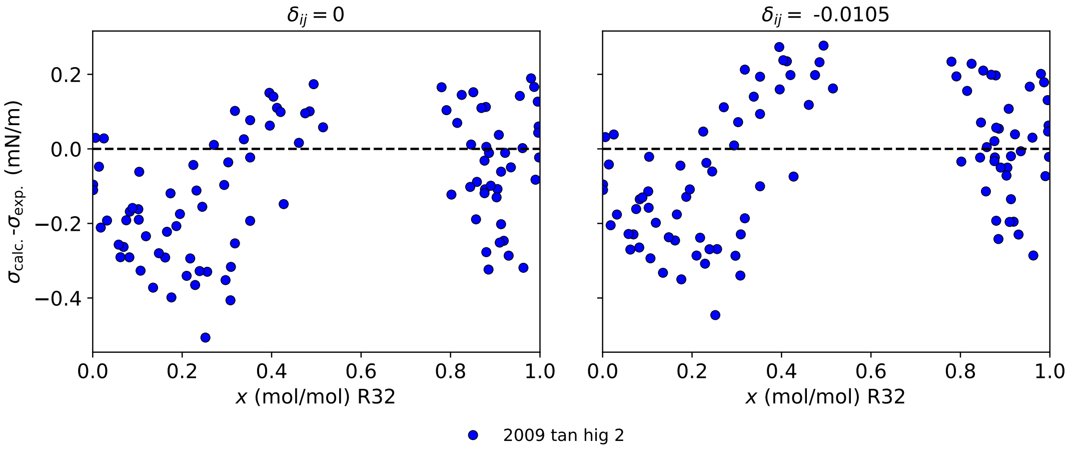

Figure 94: R32/propane

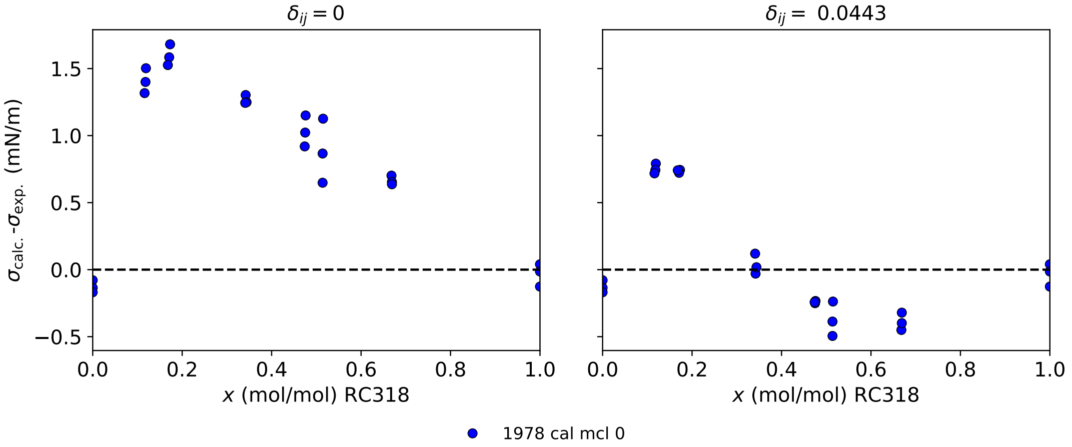

Figure 95: RC318/butane

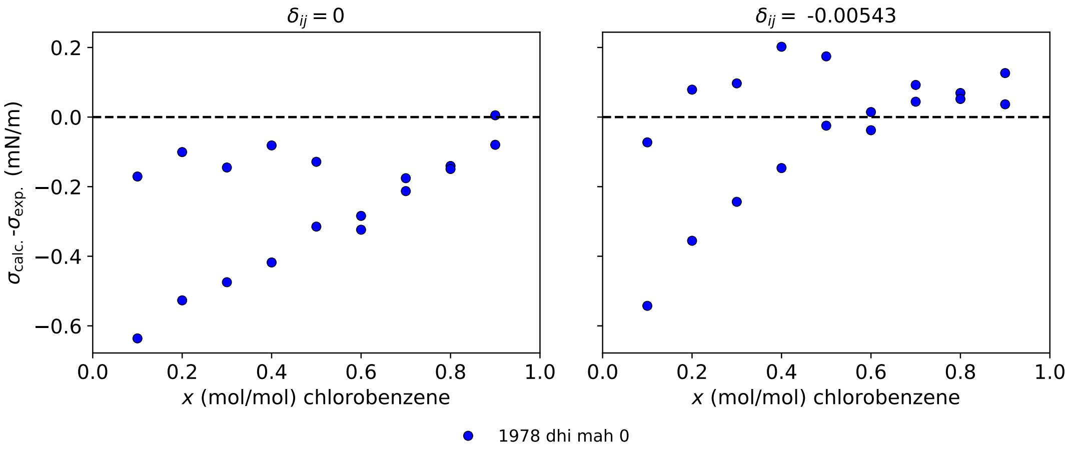

Figure 96: chlorobenzene/cyclohexane

4 Alkane mixtures

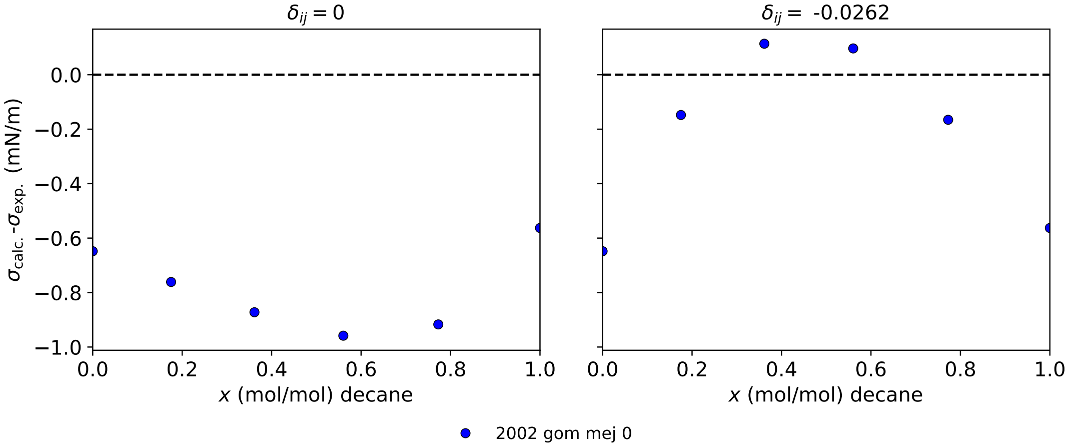

Figure 97: decane/isooctane

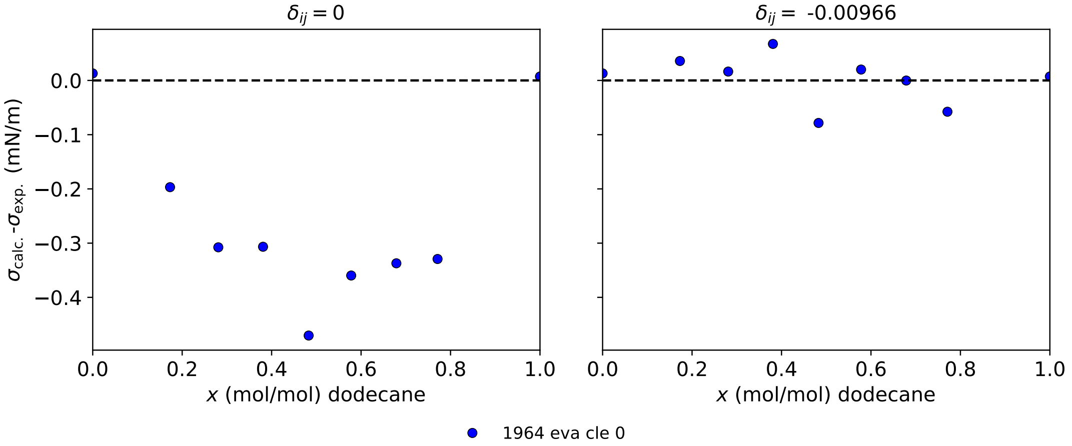

Figure 98: dodecane/isooctane

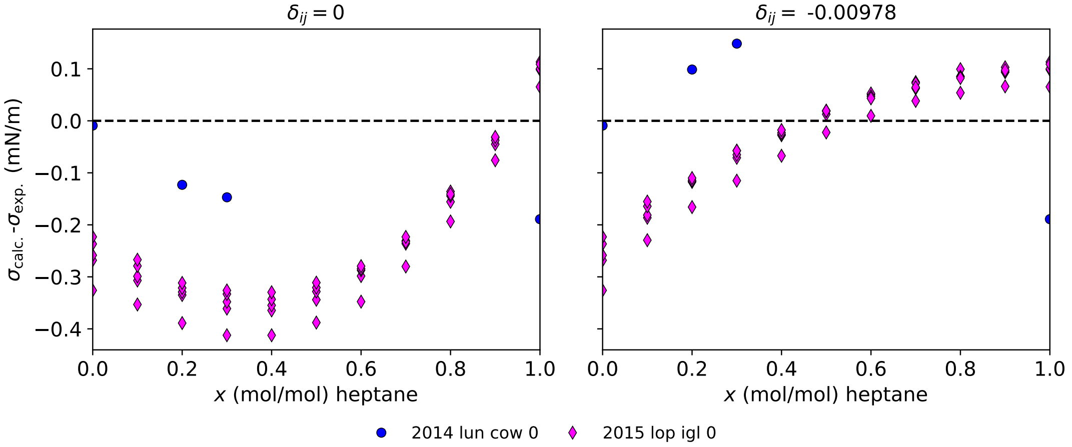

Figure 99: heptane/isooctane

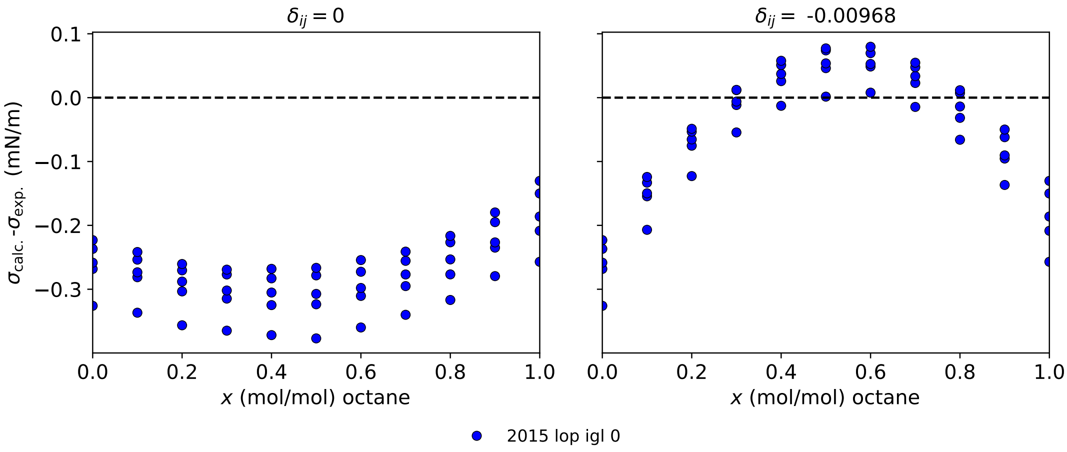

Figure 100: octane/isooctane

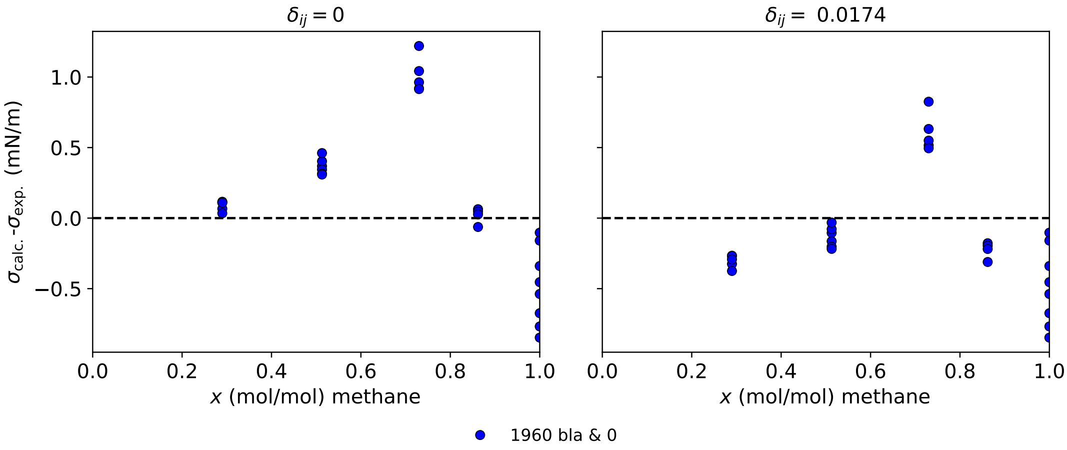

Figure 101: methane/argon

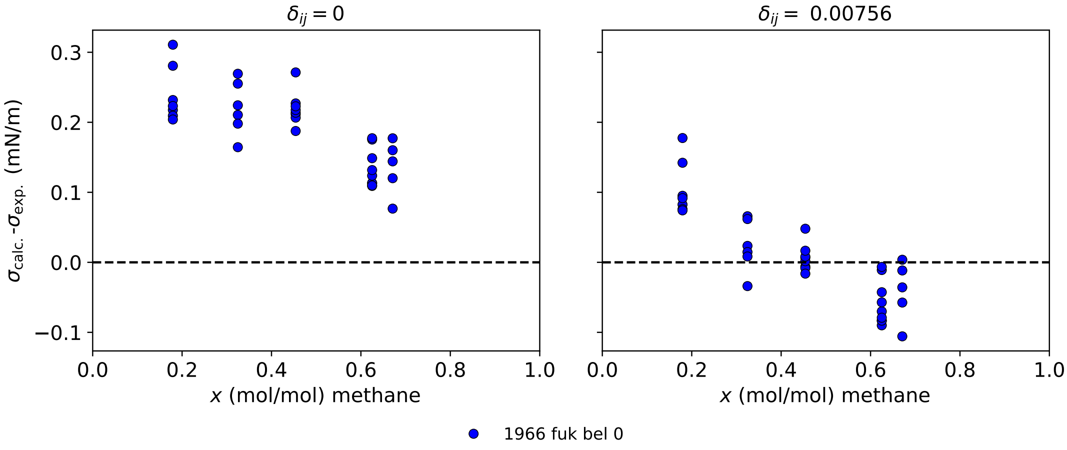

Figure 102: methane/krypton

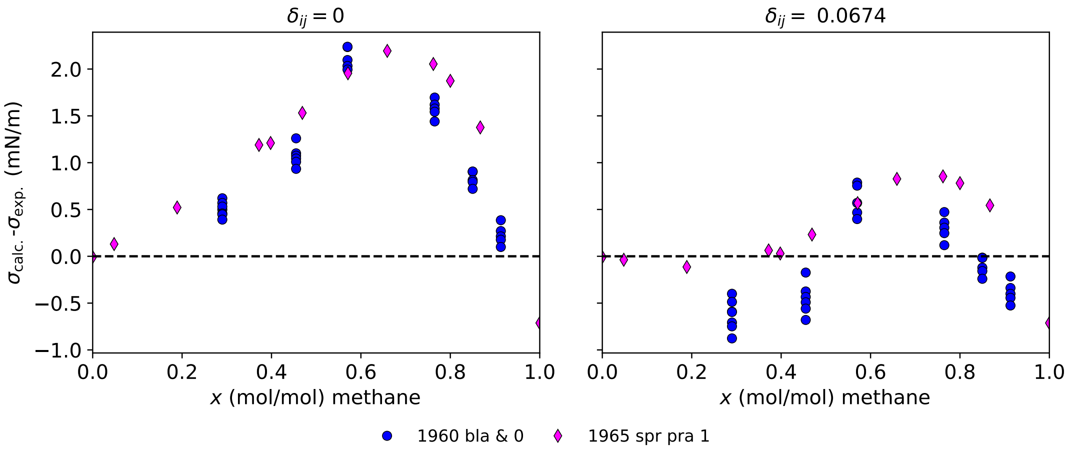

Figure 103: methane/nitrogen

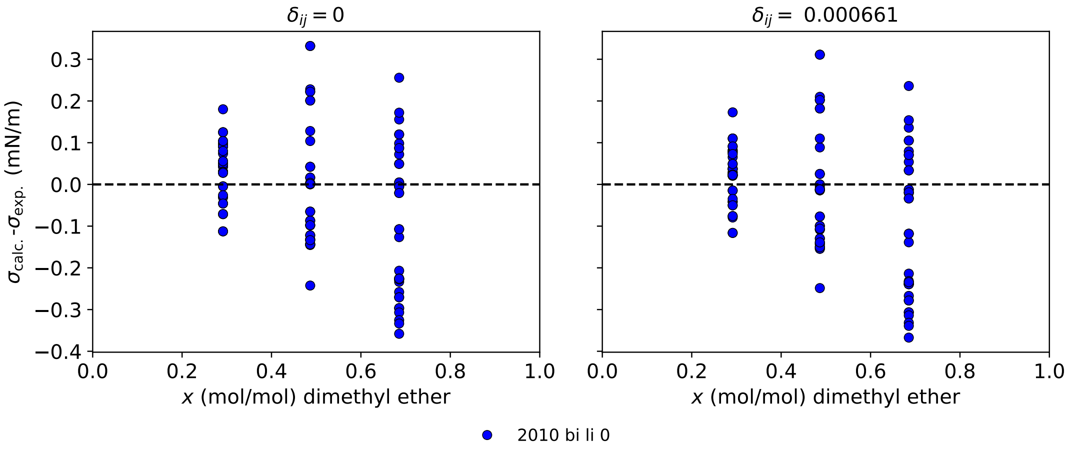

Figure 104: dimethyl ether/propane

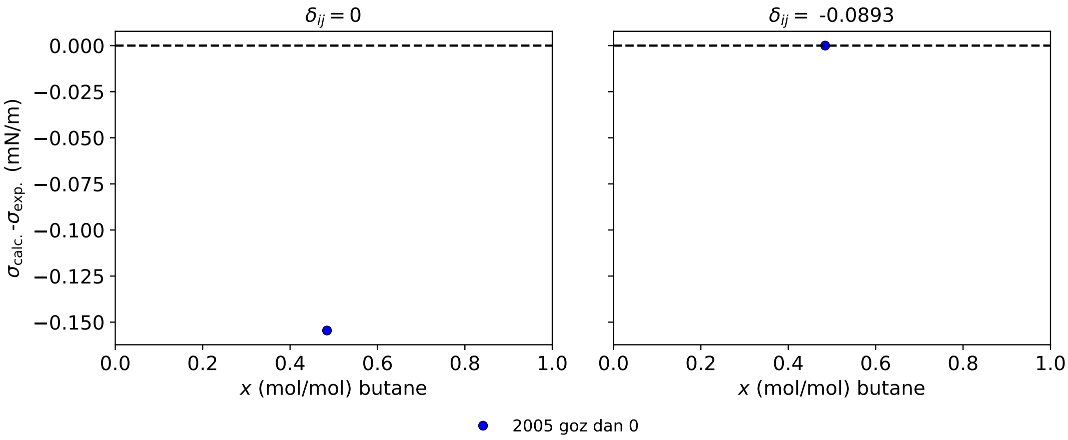

Figure 105: butane/methane

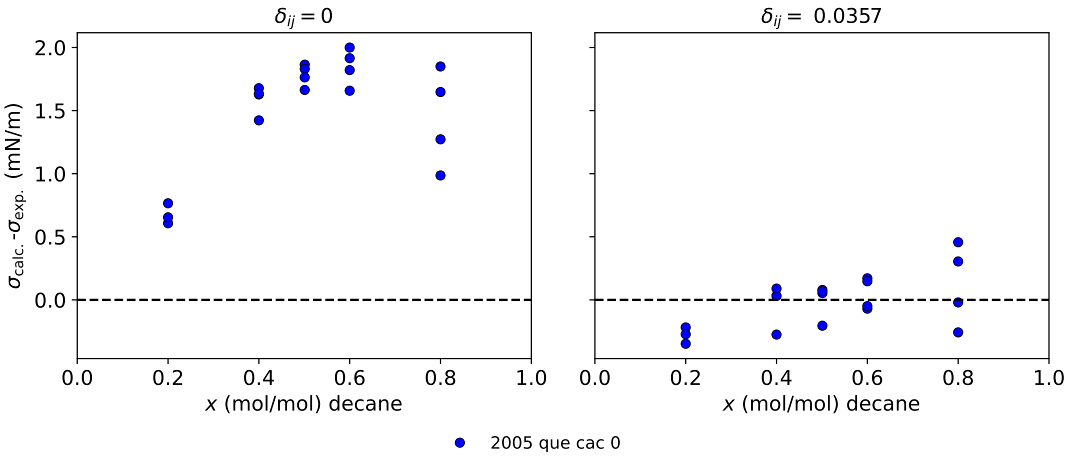

Figure 106: decane/docosane

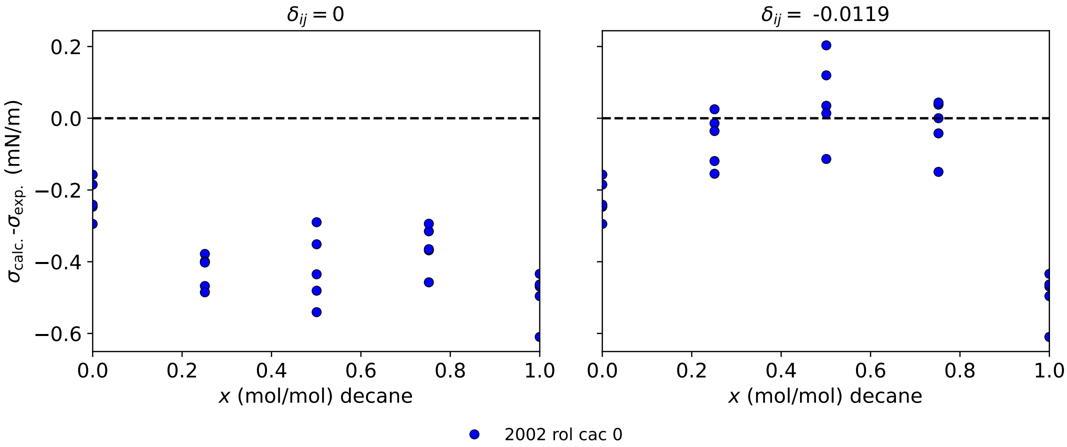

Figure 107: decane/heptane

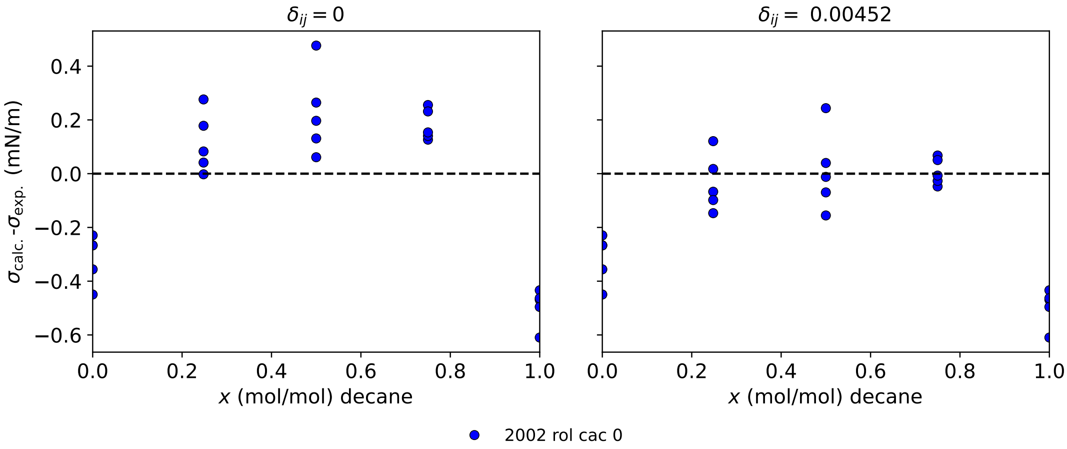

Figure 108: decane/hexadecane

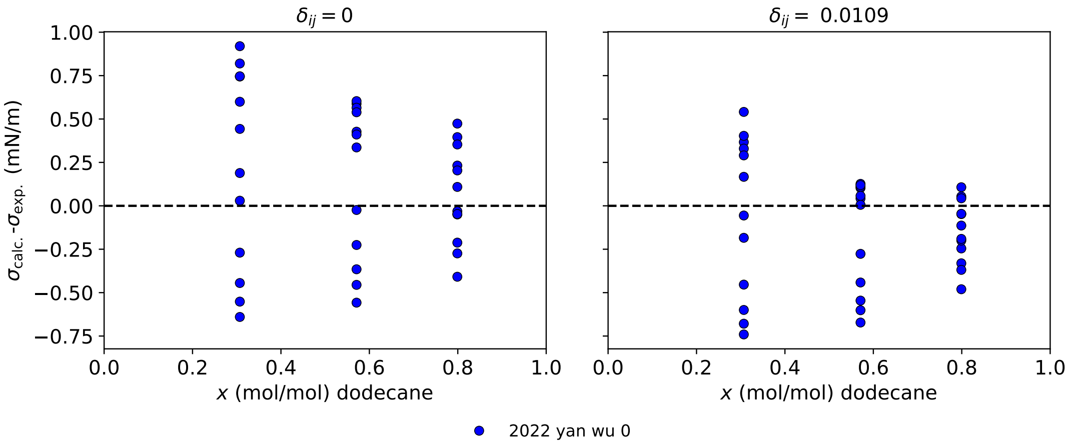

Figure 109: dodecane/hexadecane

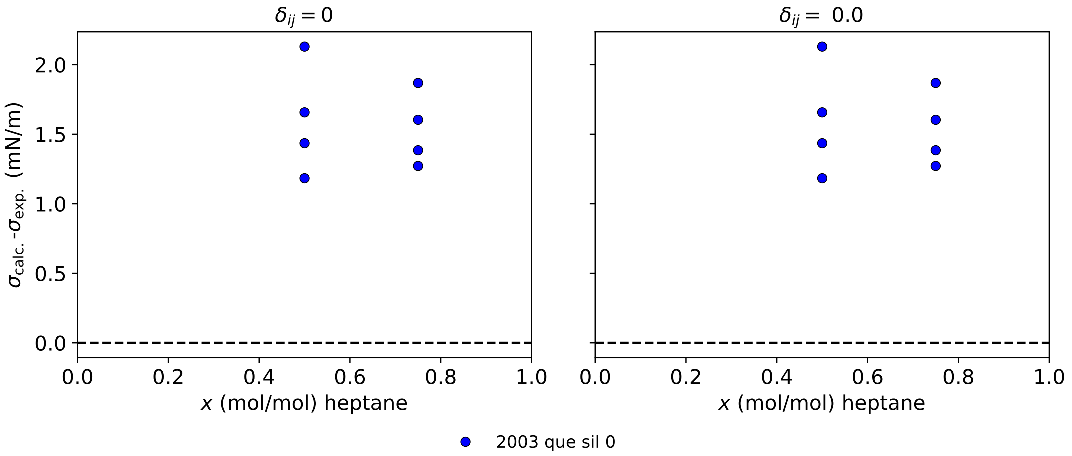

Figure 110: heptane/docosane

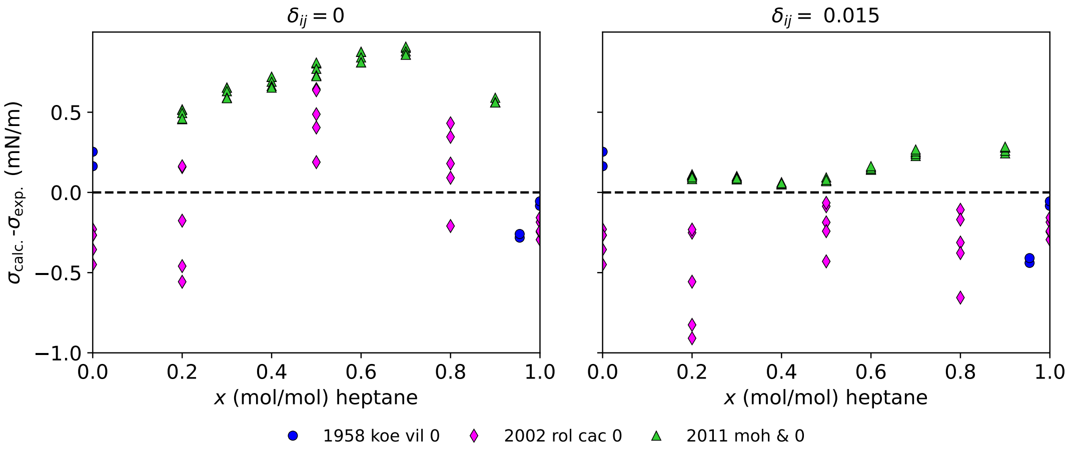

Figure 111: heptane/hexadecane

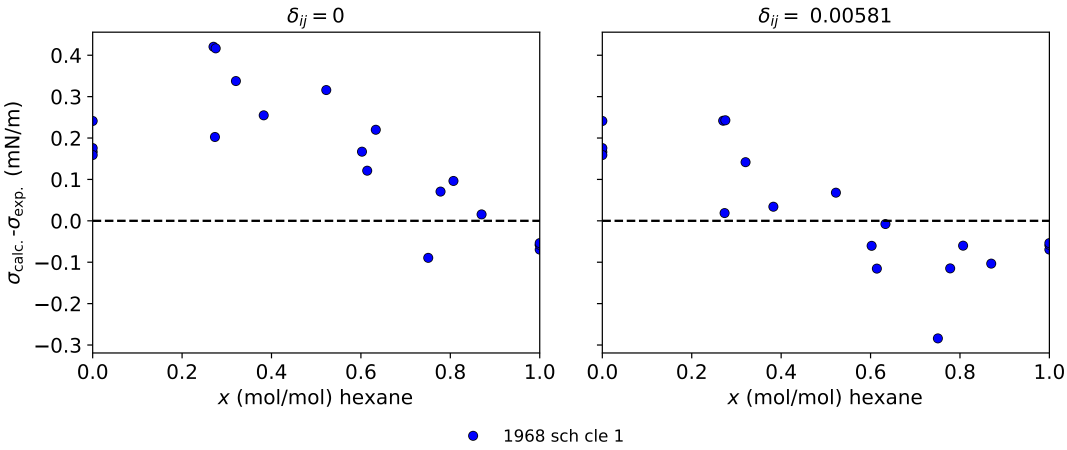

Figure 112: hexane/dodecane

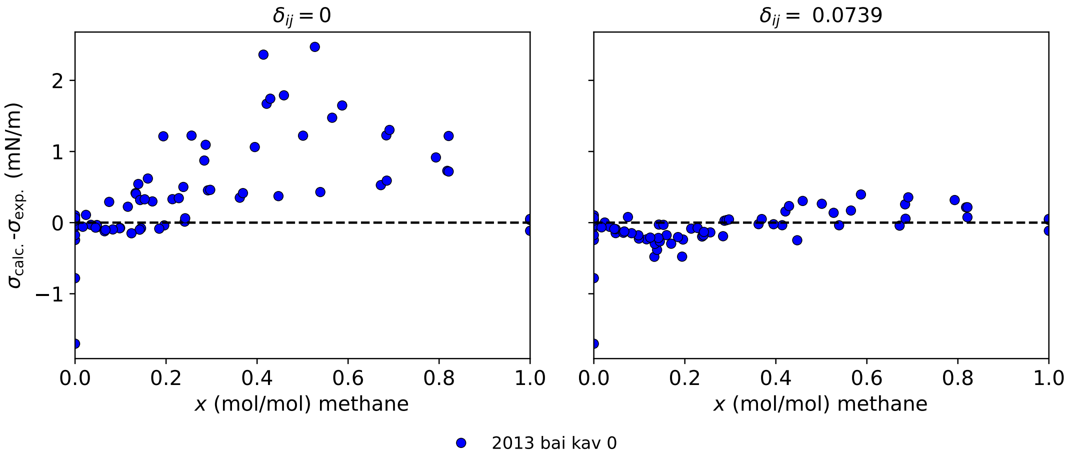

Figure 113: methane/ethane

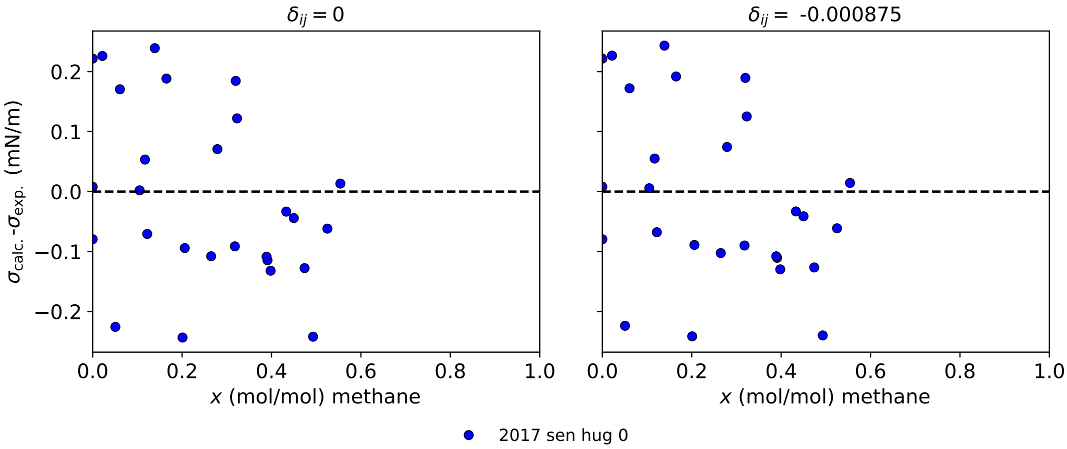

Figure 114: methane/propane

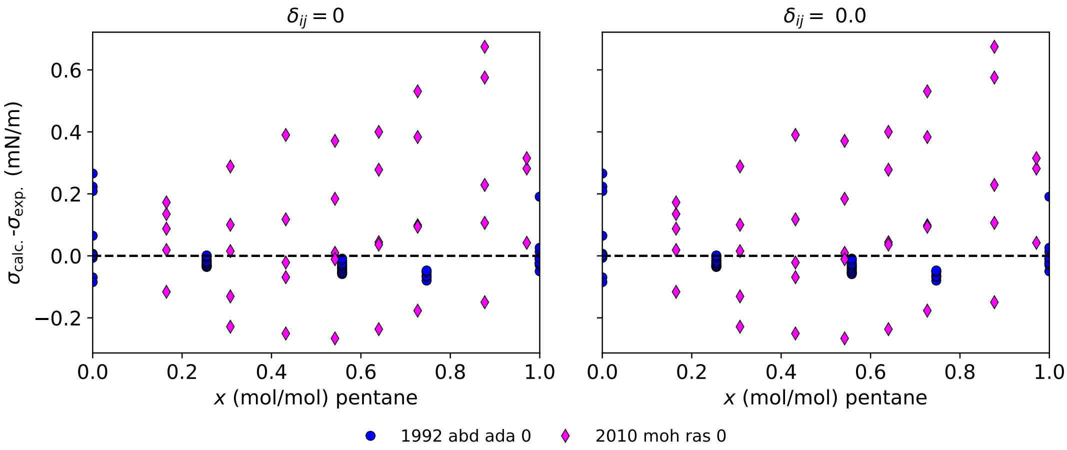

Figure 115: pentane/heptane

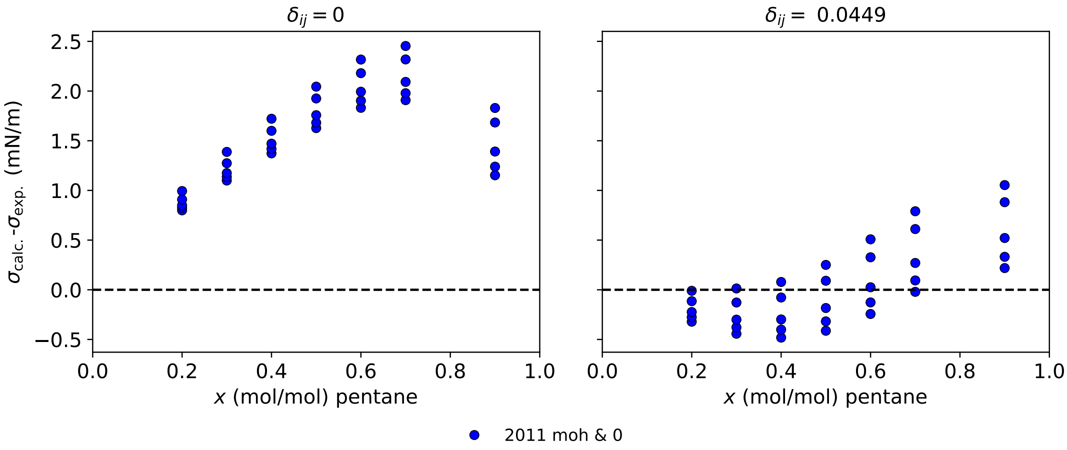

Figure 116: pentane/hexadecane

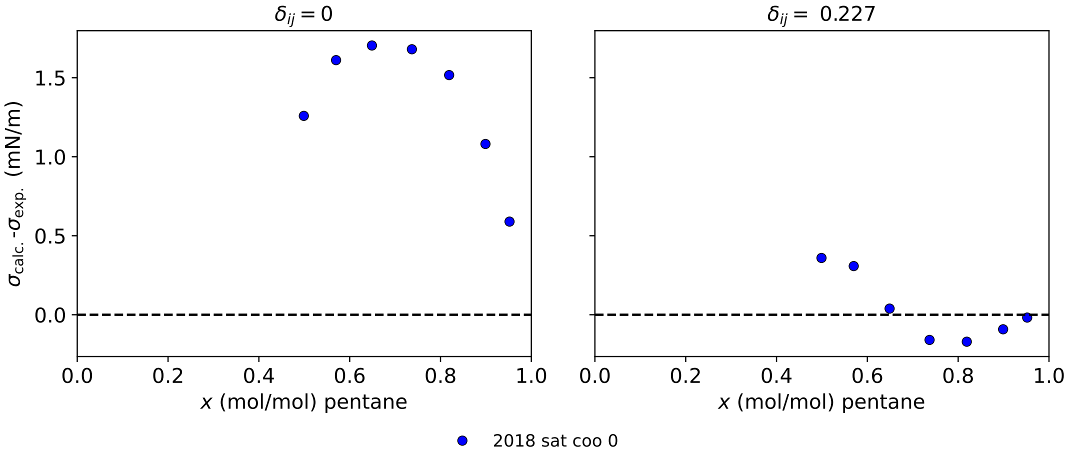

Figure 117: pentane/methane

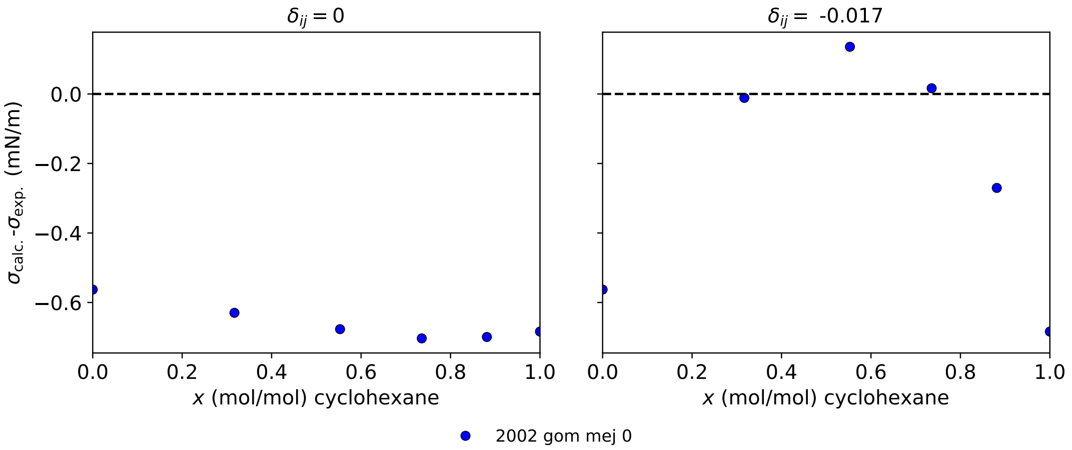

Figure 118: cyclohexane/decane

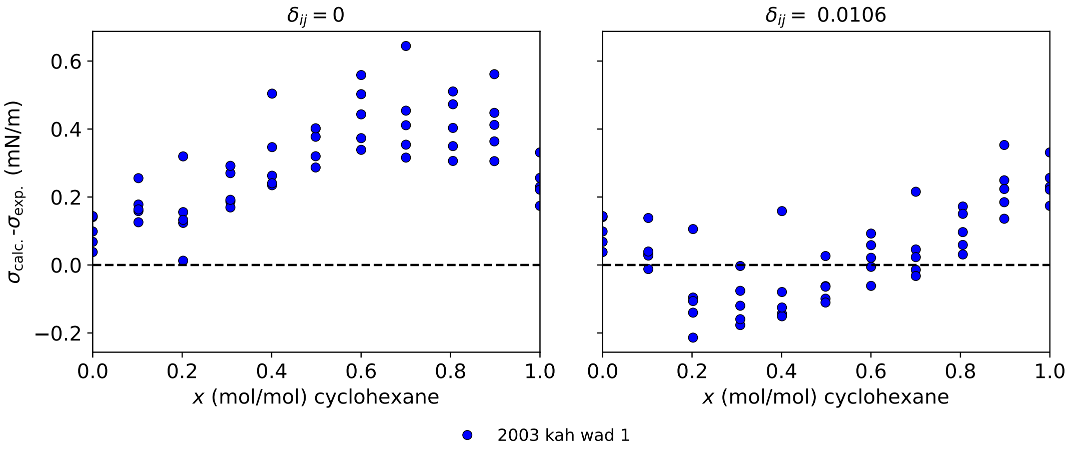

Figure 119: cyclohexane/heptane

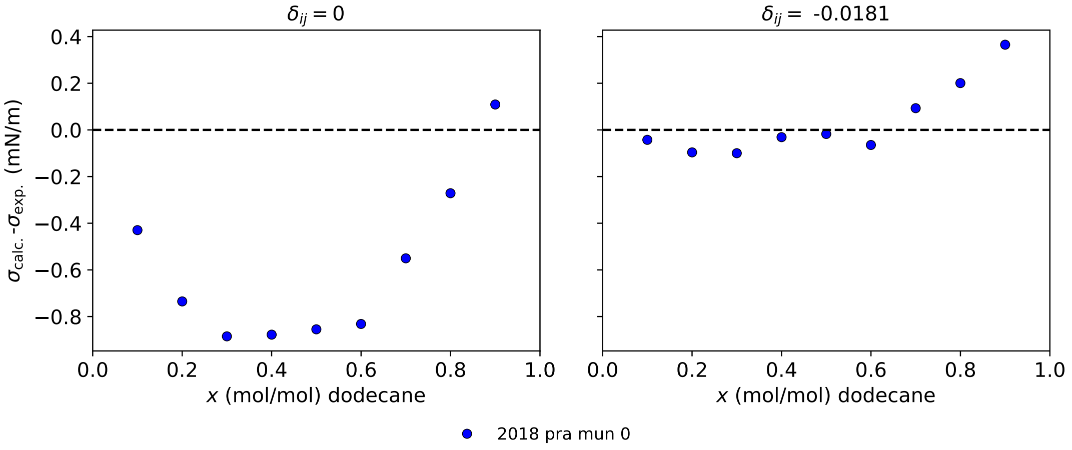

Figure 120: dodecane/methylcyclohexane

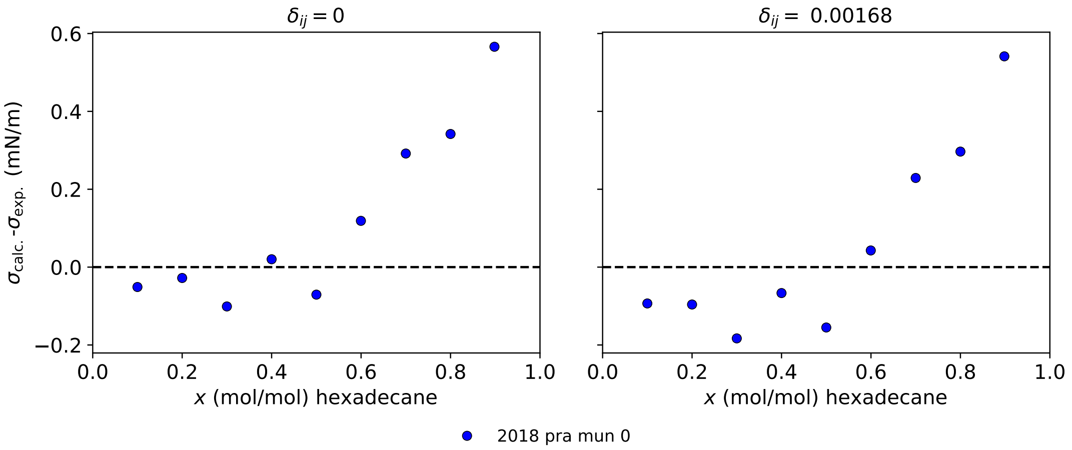

Figure 121: hexadecane/methylcyclohexane

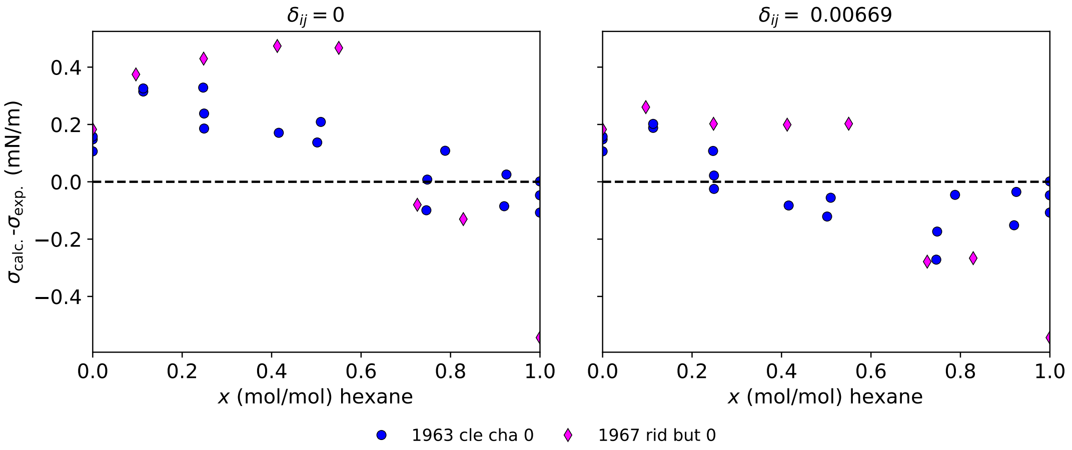

Figure 122: hexane/cyclohexane

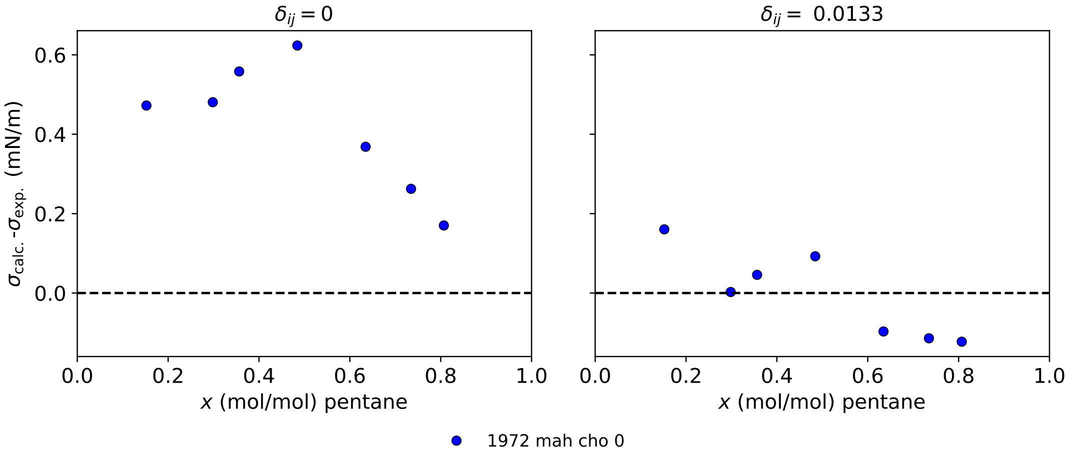

Figure 123: pentane/cyclohexane

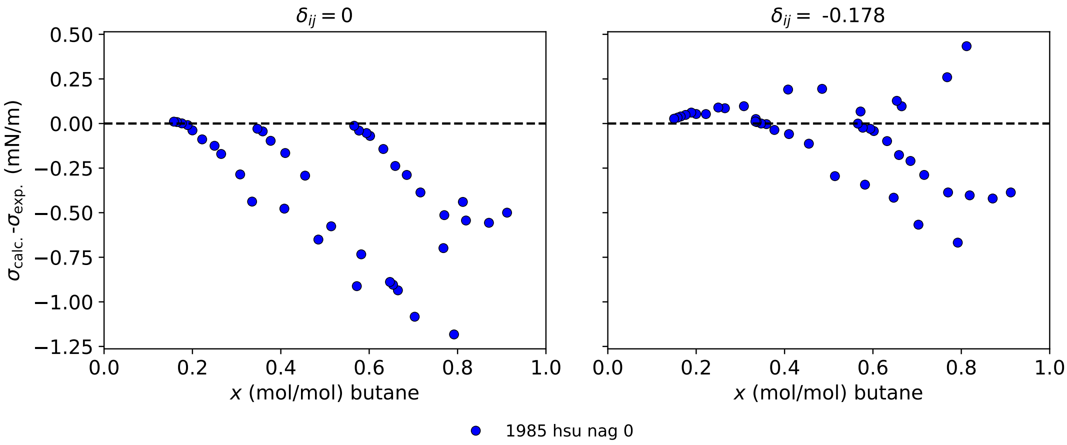

Figure 124: butane/carbon dioxide

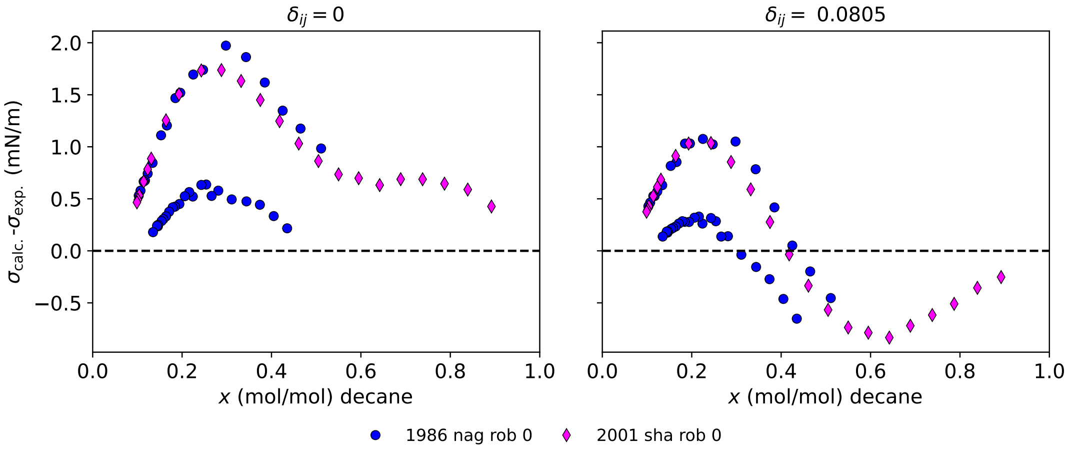

Figure 125: decane/carbon dioxide

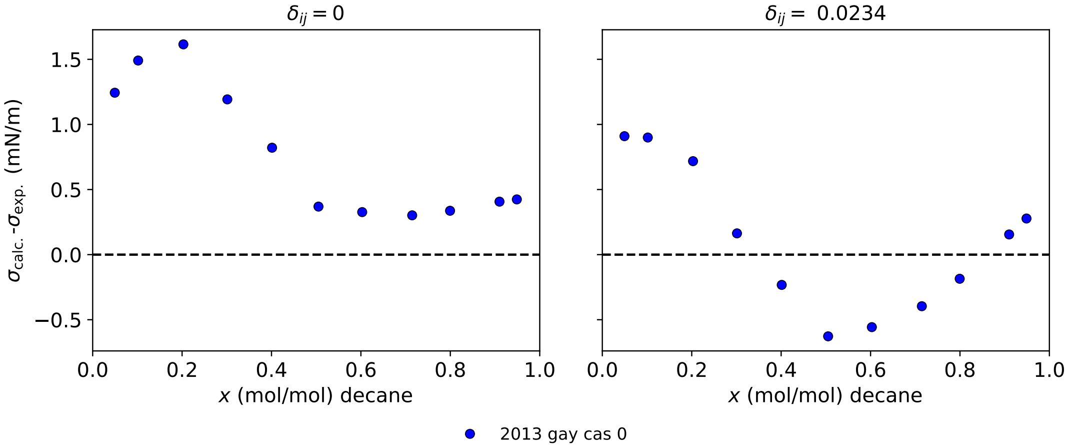

Figure 126: decane/DMC

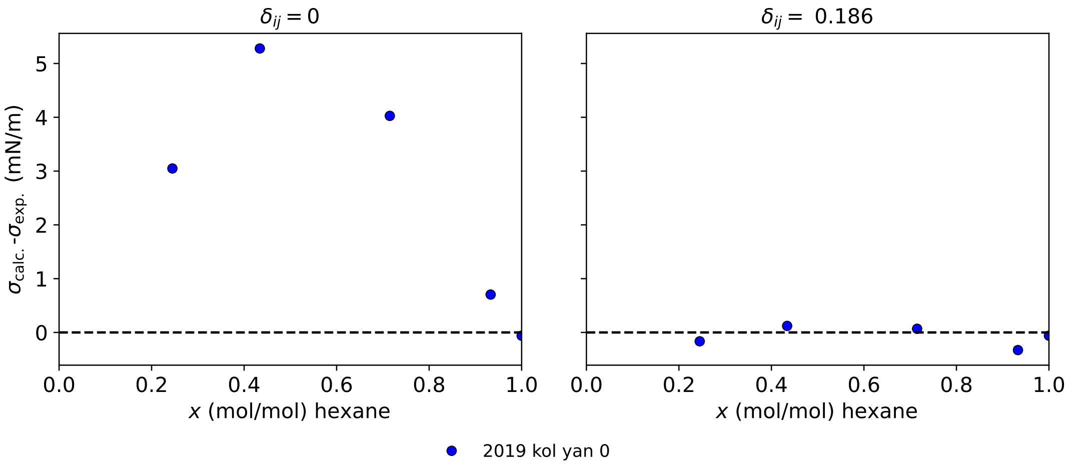

Figure 127: hexane/carbon dioxide

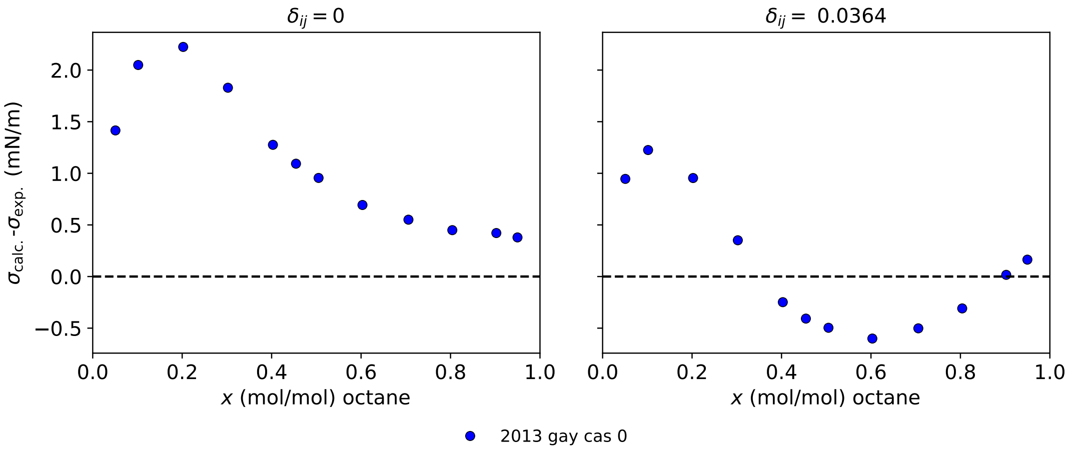

Figure 128: octane/DMC

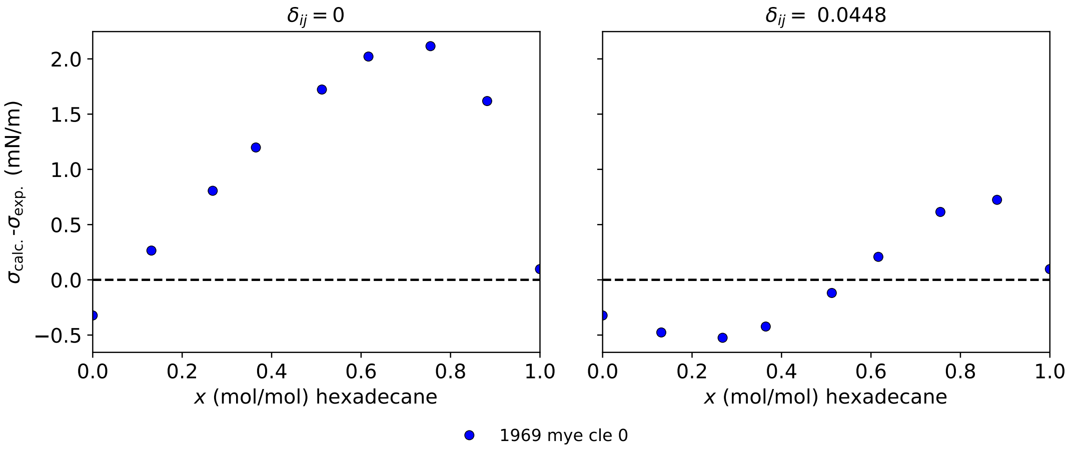

Figure 129: hexadecane/D4

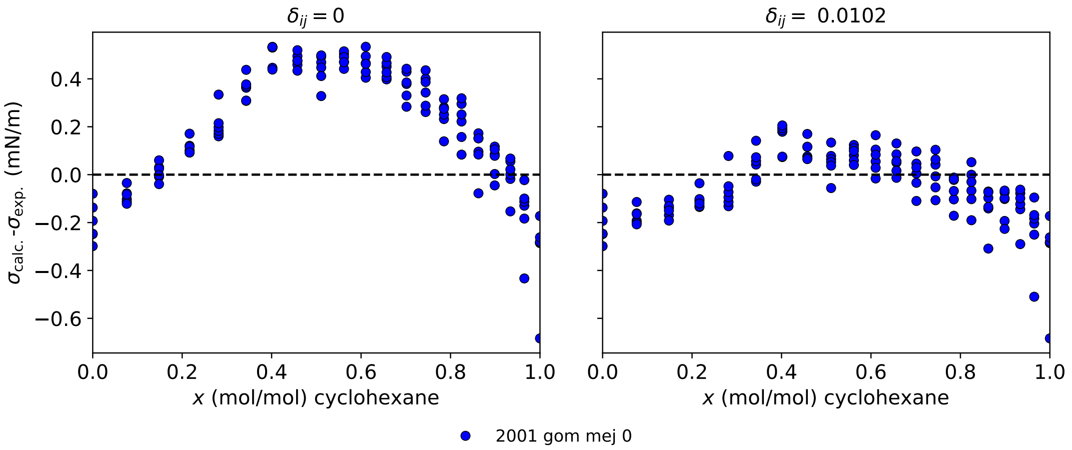

Figure 130: cyclohexane/isooctane

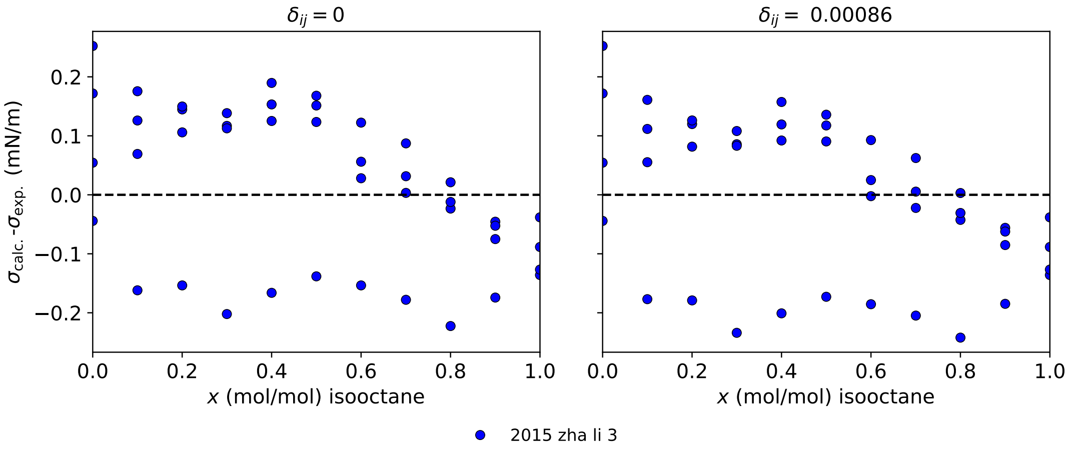

Figure 131: isooctane/methylcyclohexane

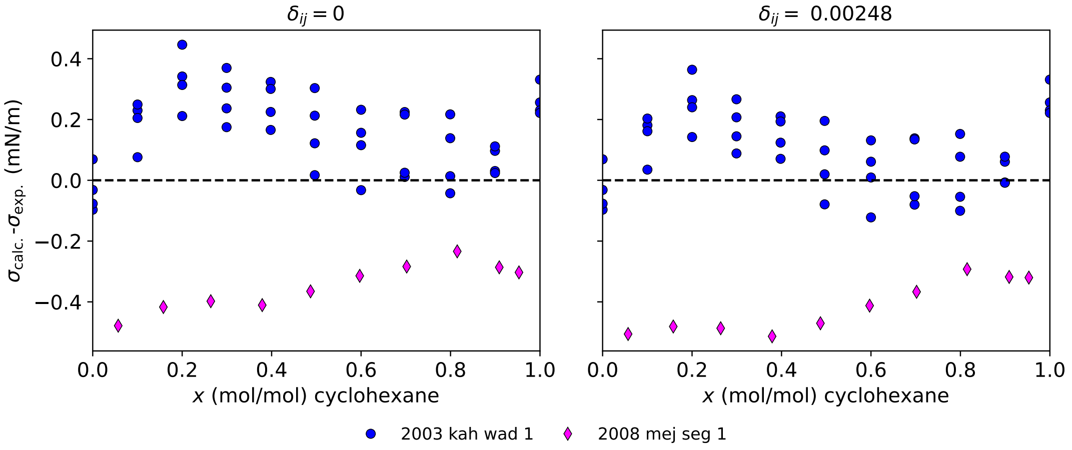

Figure 132: cyclohexane/acetone

5 Aqueous mixtures

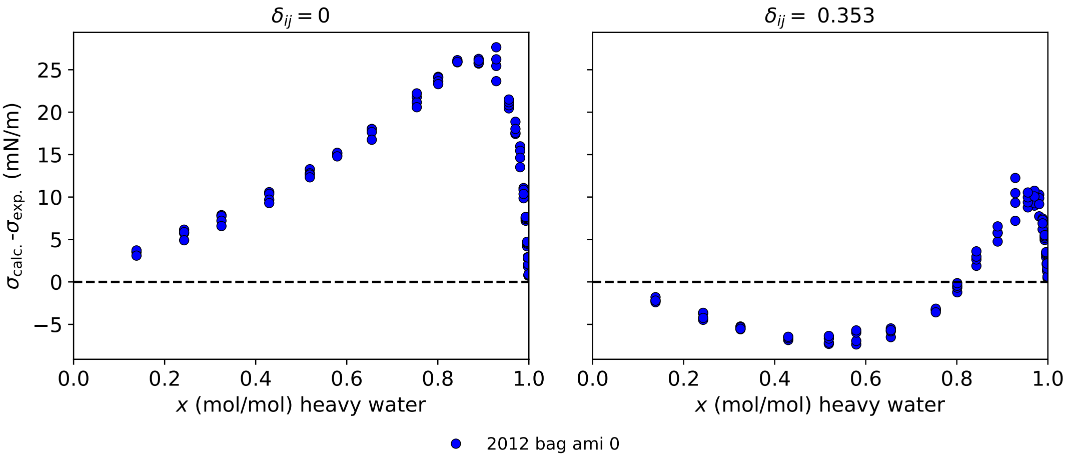

Figure 133: heavy water/ethanol

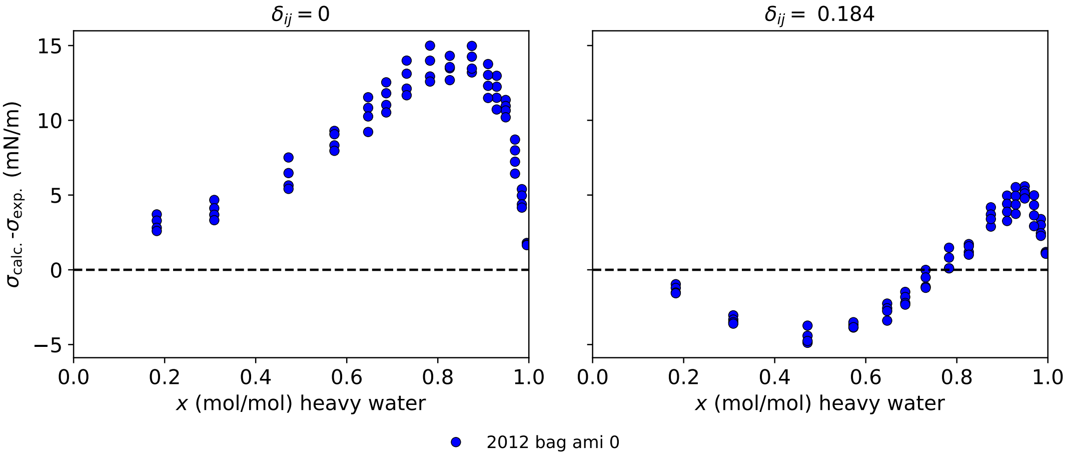

Figure 134: heavy water/methanol

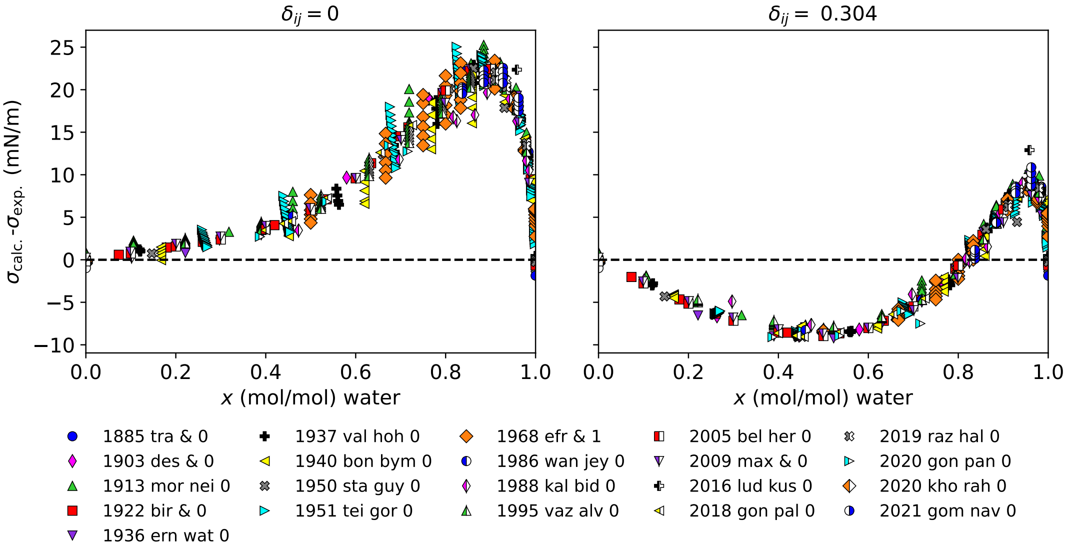

Figure 135: water/ethanol

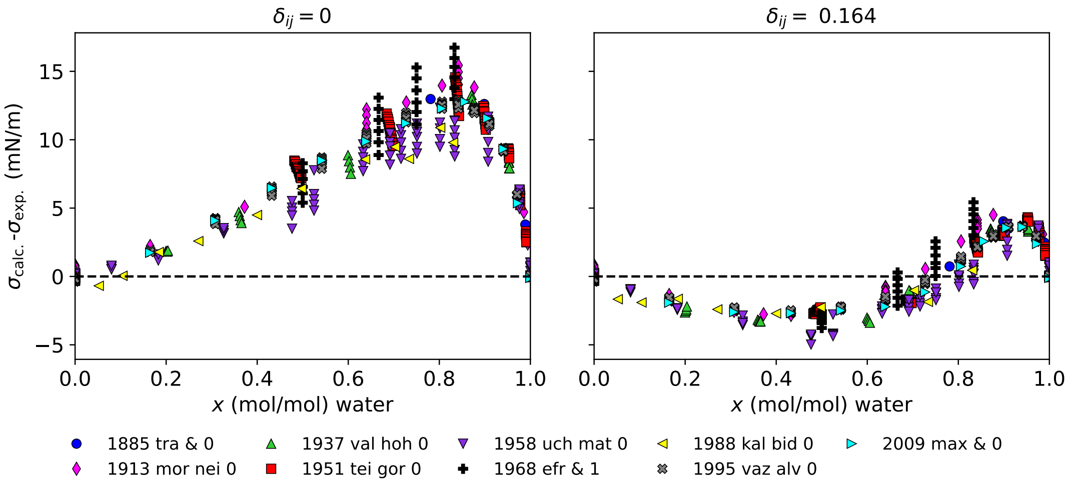

Figure 136: water/methanol

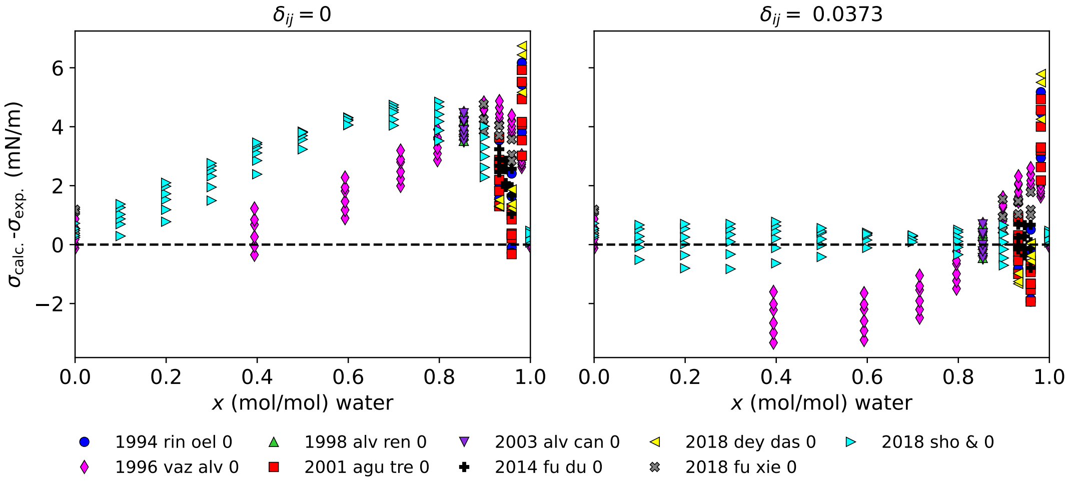

Figure 137: water/DEA

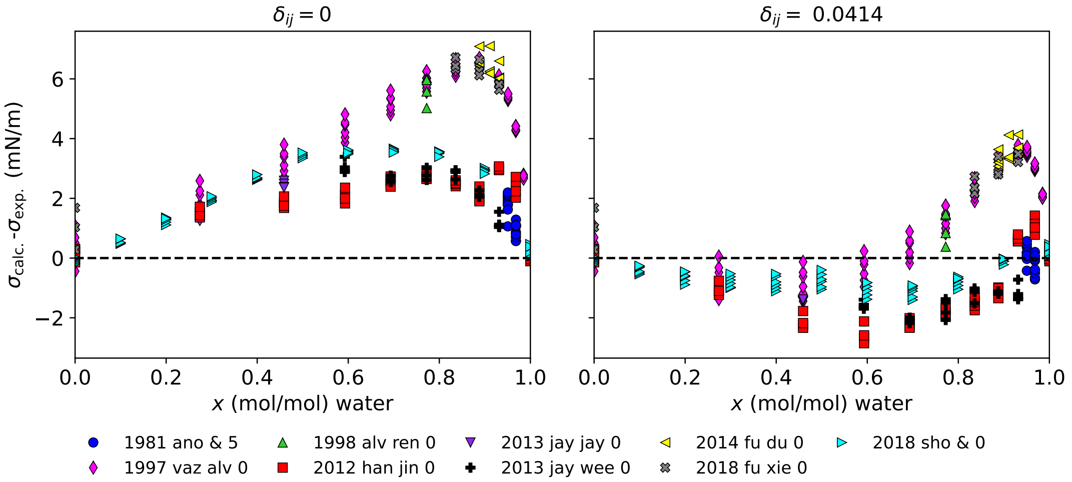

Figure 138: water/MEA

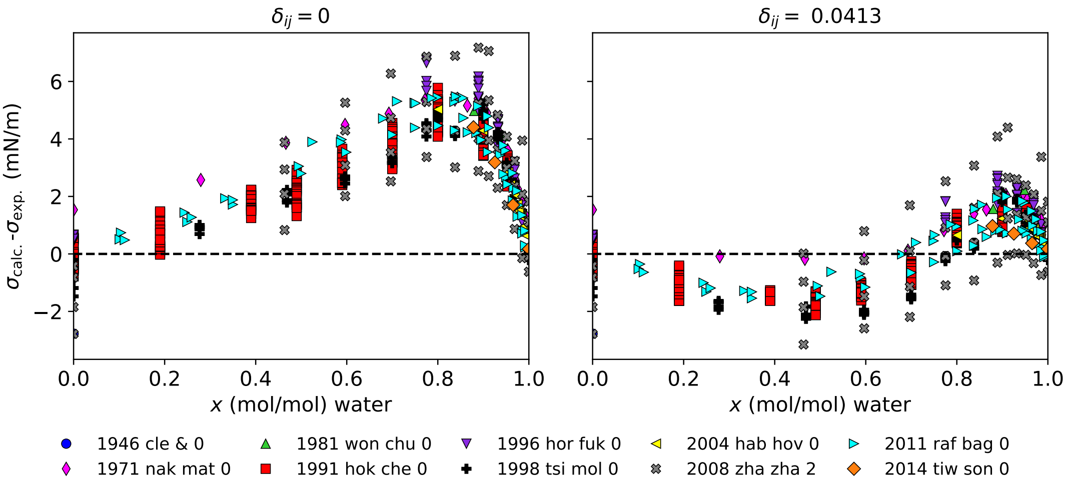

Figure 139: water/ethylene glycol

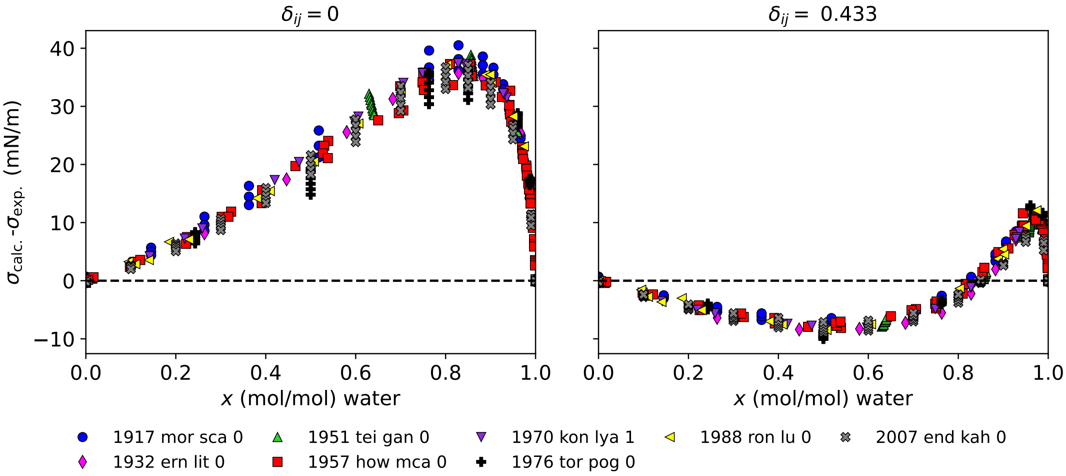

Figure 140: water/acetone

6 Misc. mixtures

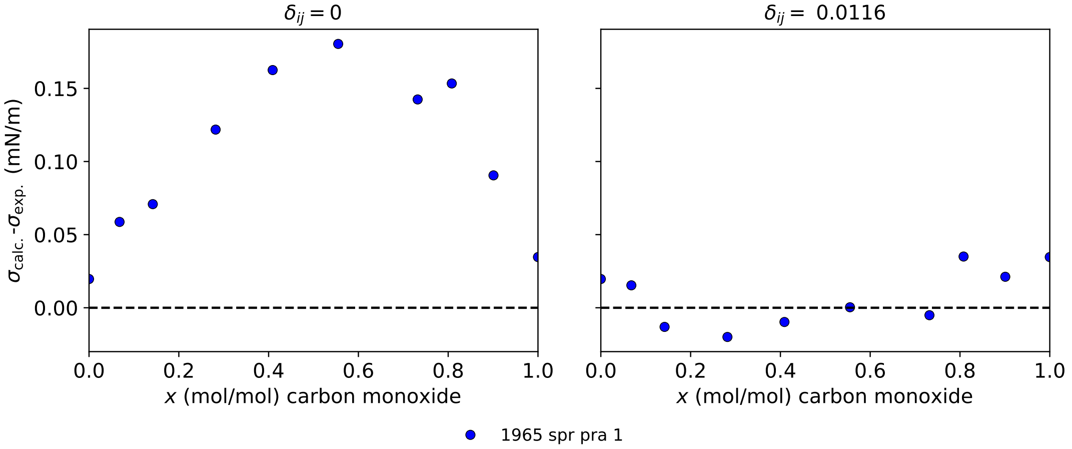

Figure 141: carbon monoxide/nitrogen

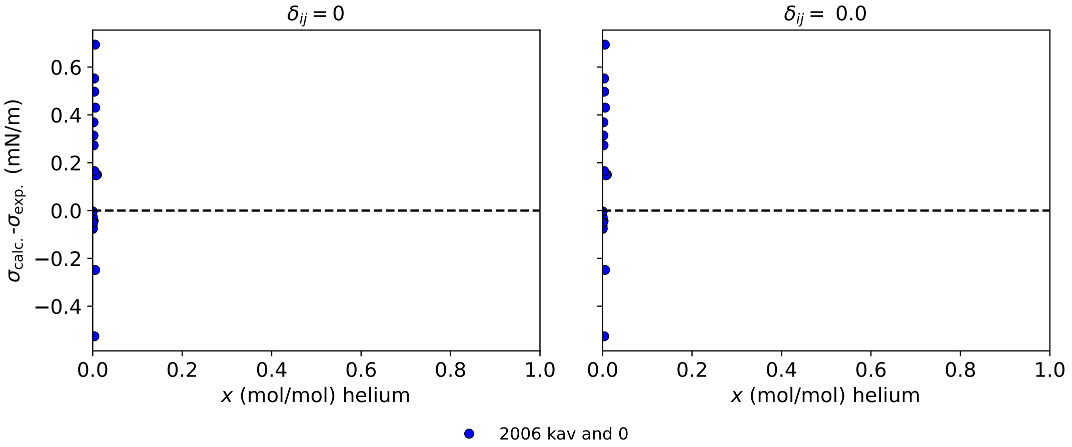

Figure 142: helium/argon

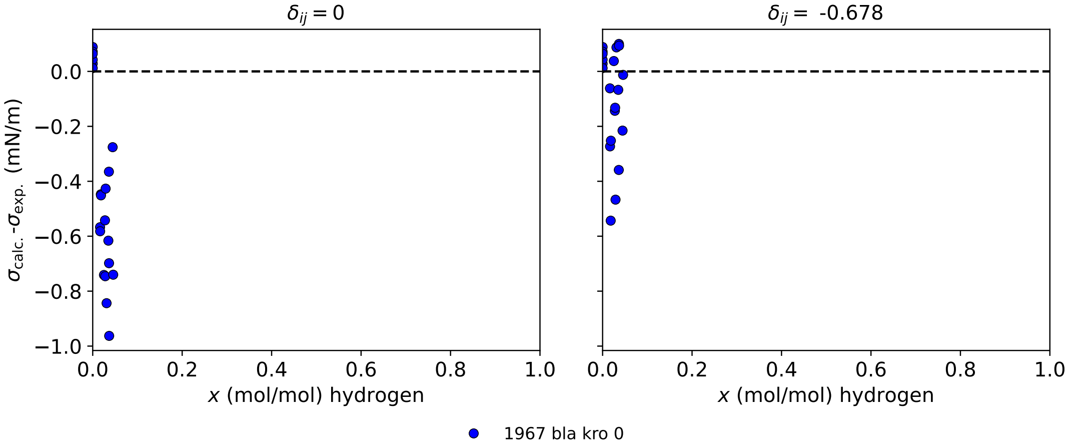

Figure 143: hydrogen/argon

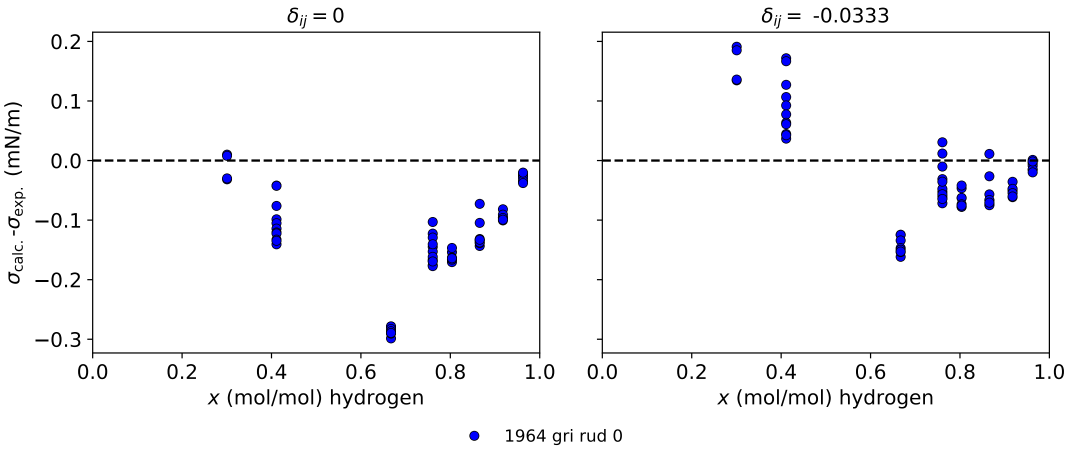

Figure 144: hydrogen/deuterium

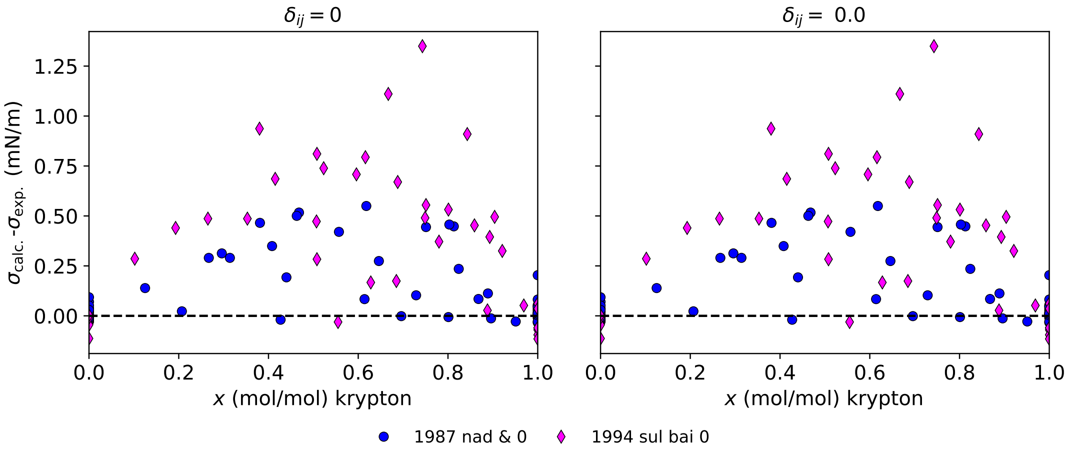

Figure 145: krypton/argon

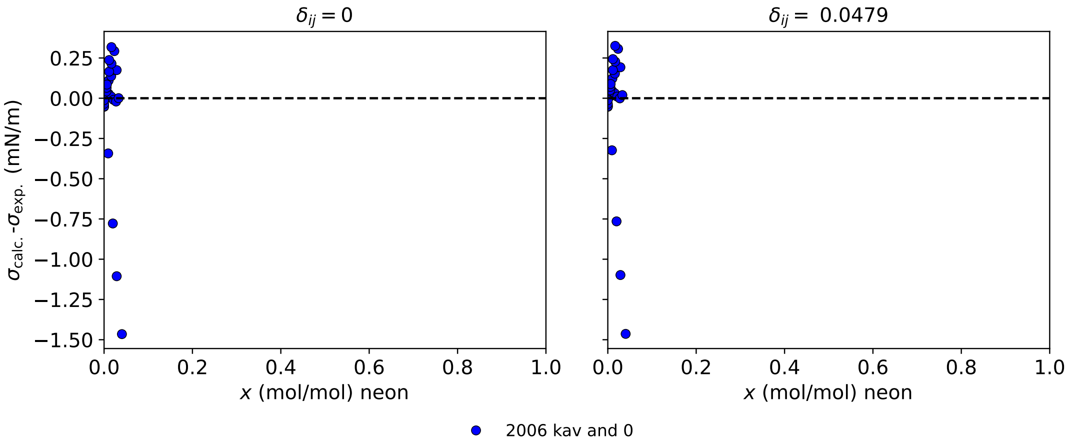

Figure 146: neon/argon

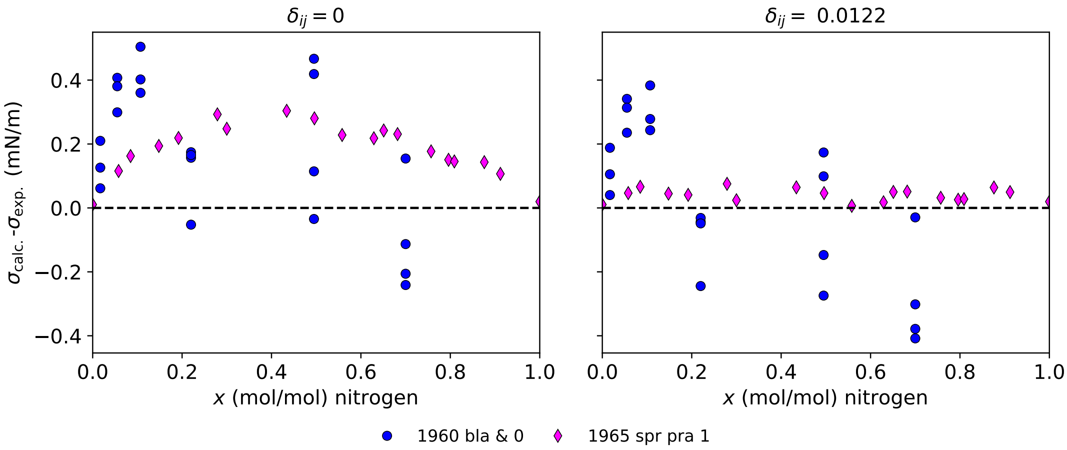

Figure 147: nitrogen/argon

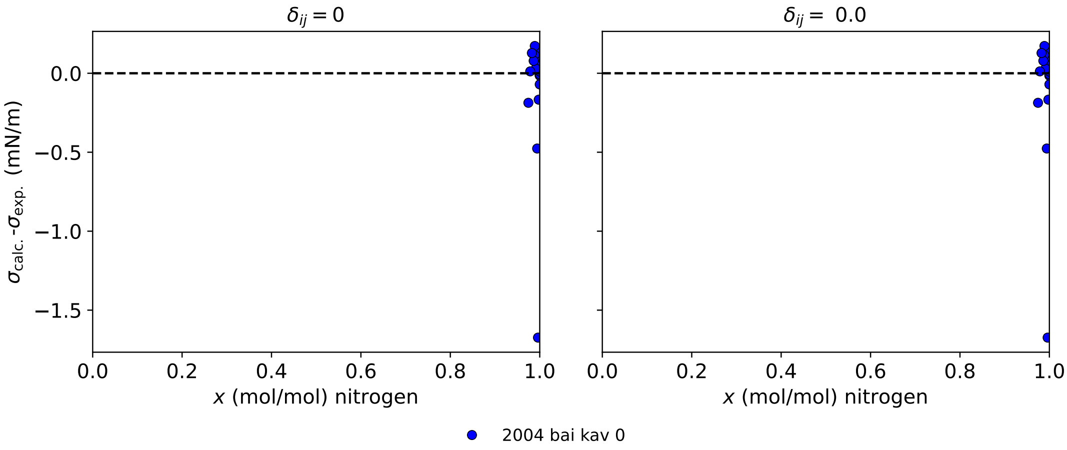

Figure 148: nitrogen/helium

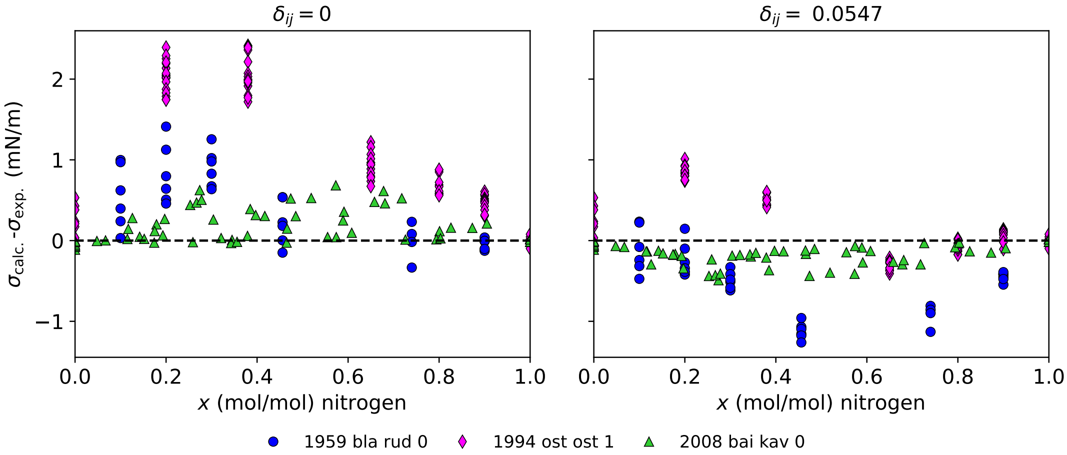

Figure 149: nitrogen/oxygen

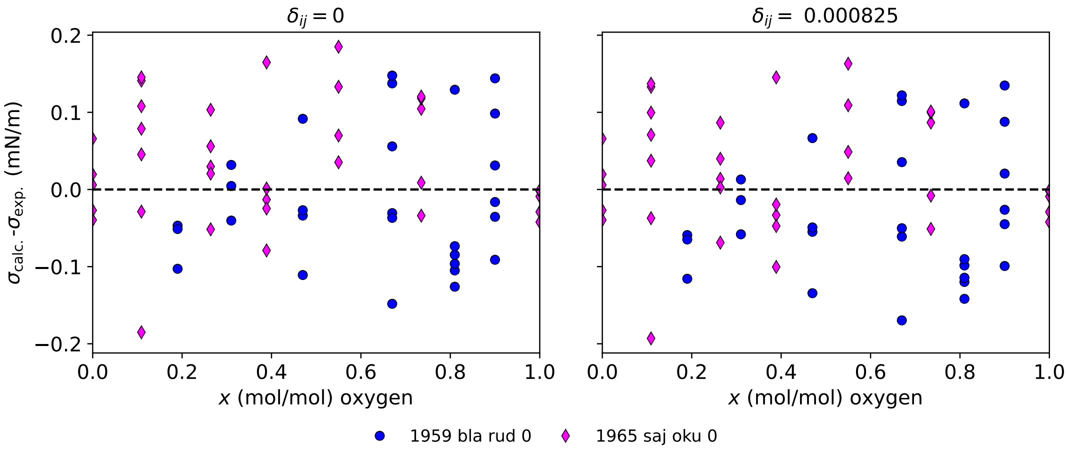

Figure 150: oxygen/argon

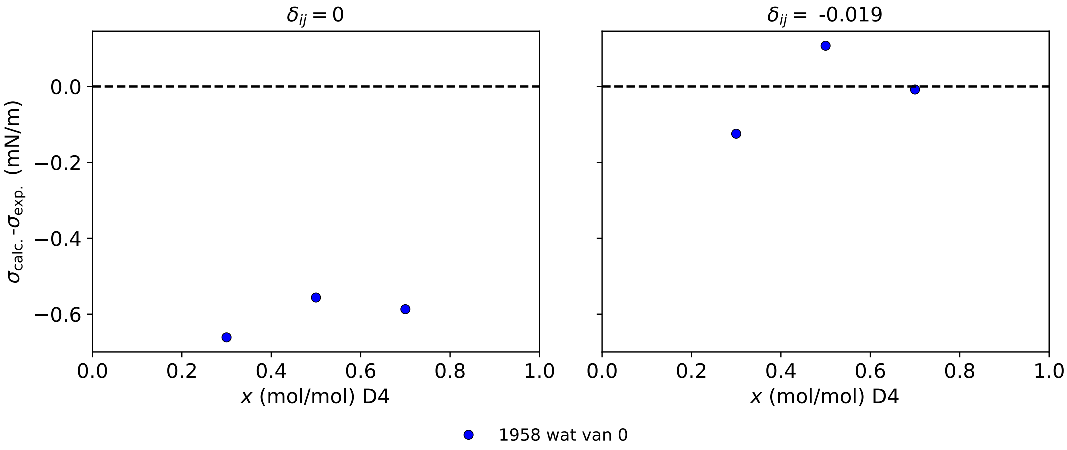

Figure 151: D4/MD2M

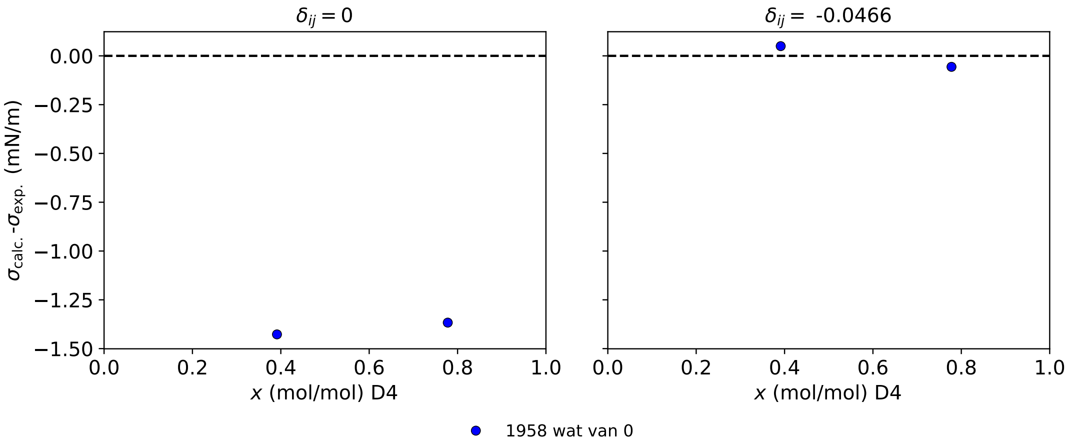

Figure 152: D4/MD4M

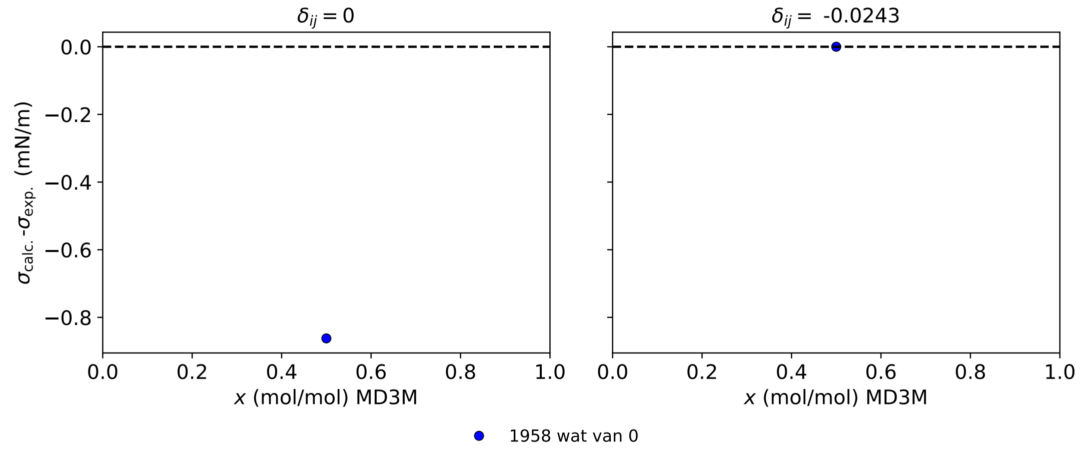

Figure 153: MD3M/D5

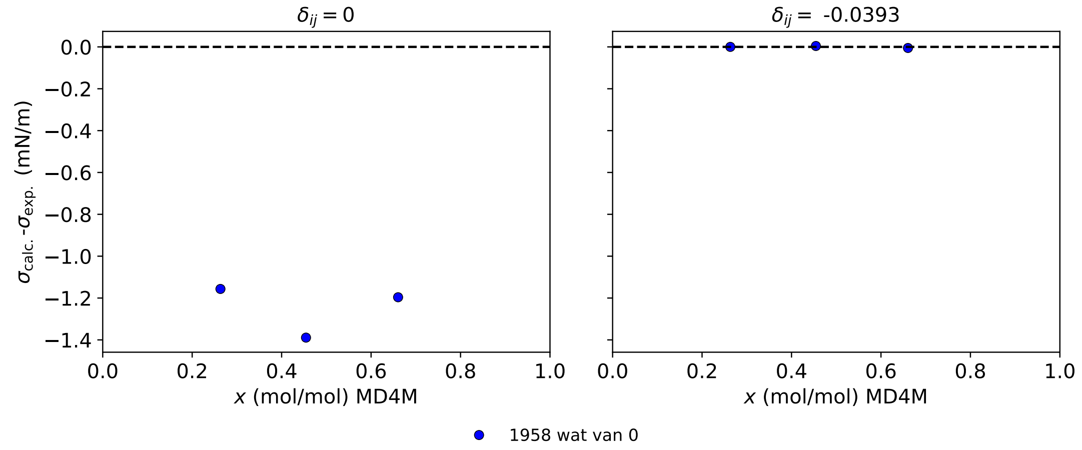

Figure 154: MD4M/D5
